# Supplementary material for: Compliance of smokeless tobacco supply chain actors and products with tobacco control laws in Bangladesh, India and Pakistan: protocol for a multicentre sequential mixed-methods study
Source: BMJ Open. 2020 Jun 17;10(6):e036468. doi: 10.1136/bmjopen-2019-036468 (PMC7304837; doi:10.1136/bmjopen-2019-036468)
Supplement: Supplementary data [file bmjopen-2019-036468supp001.pdf]

Appendices

1. Field Protocol for Data Collection
2. Product Inventory Form
3. Product In-house assessment tool
4. Point of sale observation tool
5. Point of sale Interview tool
6. Details of the in-country ethical approvals

A.

Appendix 1

ASTRA Sellers Study Field Protocol

After selection of villages and enumeration blocks in rural and urban areas respectively through ASTRA sellers study sampling strategy, data collectors will be sent to these villages and enumeration blocks for mapping of the POS and collection of samples for In-House assessment. Following the is the field protocol for Mapping and Sample collection.

Inclusion criteria for POS

A POS will be eligible for inclusion in the study if it sells any form of Smokeless Tobacco. The common types of tobacco POS that are eligible for our study include:

a. General/departmental stores
b. Petrol pump/gas station stores
c. Beer or liquor stores
d. *Paan* (betel-quid) shops
e. Grocery stores
f. Exclusive tobacco shops
g. Discount shops
h. Mobile vendors/carts
i. Stationary carts
j. Any Other _______________. (Country teams can add specific POS not in the list)

The data collectors will ascertain the eligibility of an establishment by first inquiring about the availability of ST at that establishment and then proceeding for procurement of ST samples for the "In-house analysis" component of the study. Once they have established the POS as eligible, they will drop a pin on the Google Map as per the method used for "Mapping of POS".

Walking Protocol

The data collector will observe the following guidelines to map the POS in villages and enumeration blocks.

A. *When Map of the Roads is available*

1. Obtain the map of the specific area (Village/enumeration block) with all the primary roads marked on it.
2. Mark the geographic boundaries of that village/enumeration block on the map.
3. Number all the roads for identification and ease e.g. 1,2,….n
4. Identify the start point of each road as **"A"** and end point as **"B"**.
5. Start from point **A** of the road No.1, and map all the POS available on both sides of the road until you reach point **B** of the road.
6. Repeat the process until all the roads are covered.

7. While mapping the POS at each road ask the shopkeepers for POS away from the roads. Visit those POS and map them.

B. *When Map of the Roads is not available*

1. Identify the geographic boundaries of the village or enumeration block on the map.
2. Identify and mark all the primary roads on the map with the help of key informants.
3. Number all the roads for identification and ease e.g. 1,2,….n
4. Identify the start point of each road as **"A"** and end point as **"B"**.
5. Start from point **A** of the road No.1, and map all the POS available on both sides of the road until you reach point **B** of the road.
6. Repeat the process until all the roads are covered.
7. While mapping the POS at each road ask the shopkeepers for POS away from the roads. Visit those POS and map them.

Notes:

- In case if the shop is closed, and you suspect that it as a ST POS, map it by standing at the door of the shop. Mention in the comments as "closed".
- In case of mobile vendor/carts, map at the location where you find them.

Mapping of POS

Since this component uses Images from **Google Maps**, it cannot be published. The authors are willing to share the detailed method upon request.

Purchase of Products

Data collectors will be sent in pairs to map the POS. Arriving at the first shop one data collector will purchase the products and another one will map the shop;

1. The purchaser will visit the counter and ask for all the available brands of following smokeless tobacco (ST) products.
   a) Naswar
   b) Paan/ Betel Quid
   c) Gutka
   d) Tobacco Gum
   e) Gul
   f) Snus
   g) Zarda
   h) Nasal Snuff
   i) Khaini
   j) Others__________(Country Specific)

Complete **unique packs sample** by purchasing all unique smokeless tobacco packs available.
Unique Pack Definition: any pack with at least one difference in an exterior feature of the pack including: size, brand name, presentation, colors, packaging material (i.e. hard, soft, tin), and inclusion of a promotional item.

a. Face the counter and request to purchase one of each unique ST pack.
b. Capture the price of each pack. Place a sticker on the back of the pack and record the price on the sticker.
   i. For retailers where the price is not given, do not request nor accept a reduced price if the vendor offers a discount due to the large quantity being purchased.
c. Ask the shopkeeper to confirm that one of each ST pack they sell has been acquired and purchase any additional packs identified.
d. Also acquire one of the most commonly sold cigarette pack. Put a price tag on the pack. If cigarette packs is not available with the shop, continue with the process.
e. Place the packs in a zip lock bag. Pay for the entire purchase and retain a receipt to submit for reimbursement.
   i. Record on the receipt the ID of the area and shop, number of packs purchased, total price of the purchase, and date.
   ii. If the retailer does not provide receipts use a page and record the information listed above. Put the page along with the packs in a zip lock bag and write the area and the shop ID on the bag with a permanent marker.
f. Move to the next shop and ask for ST products other than purchased at previous shop.
g. Put stickers, write price, put in zip lock bag and put the IDs.
   i. If unique packs, other than previously purchased, are not available then move to the next shop.

As **two or more teams** will be collecting data simultaneously at **different locations**. Each team will purchase the available ST products. During **In-house assessment** of the products **duplication** will be removed.

Coding of each unique product will be done during In-House assessment.

In case if the shop is closed, no consent is given, do not sell STor do not sell ST to strangers, move to the next shop. Ask each shop for new products. If you find a new product, purchase and label it. Otherwise, don’t buy duplicate product brands.

Equipment needed for field collection

Shoulder Bags (For carrying products)	Rubber bands
Zip Lock Bags	Stickers
Pens	Cash
Writing Pads	Umbrella

Data entry

An excel sheet will be given to the Data collectors. This sheet will contain information regarding the mapping of POS. It will be filled by Data collectors accordingly and will be submitted to the field supervisors on daily basis.

Appendix 2

Data fields for the digital ASTRA SLT product inventory form*

a) Study site________ (P1- P2)(B1-B2) (I1-I2)

b) Product Type_______(01-18)

c) Product unique ID________________ (Study site/Product Type/product number)

d) Name of brand__________________
E.g. Bacha Naswar, JM Gutka …………..

e) Packaged ____________________ (Yes/No)

f) Hard/soft pack/sachet _________________ (coding 1-4, 4 for other)

g) Country of make_______________ (coding 0 -195) (0 = Unknown)

h) Price (in local currency)_________________ (number)

i) Type of product ______________(coding 1-18, 18 for other)

j) Analyses status ____________ (Yes/No)

k) Price mentioned on pack_______________(Yes/No)

l) Tax detail mentioned on pack_______________(Yes/No)

If yes

m) How much amount has been mentioned ___________

*Adapted from the TPackSS online product inventory/registration form, available at https://globaltobaccocontrol.org/tpackss/

Appendix 3
In-house assessment of SLT products tool* (Adapted from TAPS guidelines)

•

**1. PACKAGING & LABELLING** (Yes =1, No = 0)	Cigarettes	Cigar/ Cigarillo	Beedi	Hukaah/shees	Naswar	Gutka	Paan/ Betel	Tobacco Gum	Mawah	Zarda	Gul	Snus	Nasal Snuff	Khaini	Others
a) Brand Name															
b) Name of the manufacturer only															
c) Address of the manufacturer only															
d) Name & Address of the manufacturer															
e) Contents listed (Yes/No)															
f) Weight per content is given (Yes/No)															
g) Country of Origin															
h) Statements of health claims															
i) Statements of harm e.g. harm to babies in the womb.															
j) Written health warnings (Yes/No)															
k) The language of health claims, Harm, Warnings (if No skip to k)															
I. Regional language															
II. National language															
III. English															
IV. Another foreign language															
l) Pictorial Health warning (Yes/No)(if No then skip to “m”)															
m) The proportion of package does it cover															
I. 25%															

II. 50%															
III. 75%															
IV. 100%															
n) Provision of quit information on packaging (Yes/No)															
o) Use of culturally specific references (such as special images, symbols, or colours, etc.) (see your law or speak to local advocates for details)															
p) Presence of double packs															
q) Use of words that suggest added flavour (Yes/no)															
r) Use of words that suggest reduced strength (Yes/no)															

**2. PRICE AND TAXATION** (Yes =1, No = 0)	Cigarettes	Cigar/ Cigarillo	Beedhi	Hukaah/sheesha	Naswar	Gutka	Paan/ Betel Quid [illegible]	Tobacco Gum	Mawah	Zarda	Gul	Snus	Nasal Snuff	Khaini	Others
In what form is the product sold?															
a) Loosely sold?															
b) Loose, Mixed with other products (custom made)															
c) Packaged or wrapped by the retailer															
d) Pre-packaged by manufacturer															
e) If pre-packed then in the form of															
I. Packet															
II. Tin															
III. Bottle															
IV. Can															
V. Sachet															
VI. Box															

VII. If other please specify															
VIII. With branding															
f) The minimum unit of sale															
I. By quantity															
II. By weight															
g) Price of Unit customer pays															
h) Price of Unit retailer pays															

*this is the general tool. Countries are allowed to add any country specific provisions into the items.

Appendix 4
POS observation tool

ASTRA - POINT OF SALE OBSERVATION TOOL (Adapted from STARS)

1. Date of visit:__/__/____ Start Time: __:__ End Time: __:__
2. Coder Name/ID:________________________
3. Point of Sale ID:_____/ _______ (Study site code/serial number)
4. Store Type: (Choose one)
   a) General/Departmental Store ☐
   b) Petrol pump/Gas station store ☐
   c) Beer or Liquor store ☐
   d) Grocery store ☐
   e) Exclusive Tobacco shop ☐
   f) Tea Stall ☐
   g) Mobile cart ☐
   h) Stationary cart ☐
   i) Other (specify e.g. Discount shop):____
5. Can you survey this store? [If not, then select an option below and STOP.]
   a) Yes, I can ☐
   b) No, store does not exist ☐
   c) No, store is closed ☐
   d) No, environment unsafe for me ☐
   e) No, asked to leave before completing the survey ☐
   f) Other (specify):____

SECTION 1: OBSERVATION (POS display for mobile or stationary cart should be considered exterior)

6. **EXTERIOR**

Are there any point-of-sale displays of the tobacco products **OUTSIDE** the shop? (Yes = 1, No =0)	Cigarettes	Cigar/ Cigarillo	Beedi	Hukaah/sheesha	Naswar	Gutka	Paan/ Betel Quid [illegible]	Tobacco Gum	Mawah	Zarda	Gul	Snus	Nasal Snuff	Khaini	Others
a) Shelf edging															
b) Dummy packs															
c) Display packs															
d) Display stands															
e) Any other types of advertising?															

7. Is the point of sale vendor within _________meter (average distance) vicinity of any educational institution?

8. Does store display a health warning sign (OUTSIDE) against tobacco use? (If No skip to 9) ☐

Products	Written	Graphical

a) Smoking tobacco		
b) Smokeless Tobacco		

INTERIOR

9. Does the store have a pharmacy counter? (Yes = 1, No =0) ☐

10. Does store display a health warning sign (INSIDE) against tobacco use? (If No skip to 11) ☐

Products	Written	Graphical
a) Smoking tobacco		
b) Smokeless Tobacco		

11.

Are there any point-of-sale displays of the tobacco products **INSIDE** the shop? (Yes = 1, No =0)	Cigarettes	Cigar/ Cigarillo	Beedi	Hukaah/sheesha	Naswar	Gutka	Paan/ Betel Quid	Tobacco Gum	Mawah	Zarda	Gul	Snus	Nasal Snuff	Khaini	Others
a) Shelf edging															
b) Dummy packs															
c) Display packs															
d) Display stands															
e) Any other types of advertising?															

12.

**PRODUCTS OBSERVATION** (Yes = 1, No = 0)	Cigarettes	Cigar/ Cigarillo	Beedi	Hukaah/sheesha	Naswar	Gutka	Paan/ Betel Quid	Tobacco Gum	Mawah	Zarda	Gul	Snus	Nasal Snuff	Khaini	Others
a) Sold here?															
b) Visible															
c) Loosely sold here?															
d) Loose, mixed with other products (custom made)															
e) Product within 1 feet range of confectionaries, toys, candy, gum, slushy/soda machines, or ice cream?															
f) Products within 3 feet range of floor?															
g) Any price promotions/Credit?															

h) Cross-product promotion with tobacco? (Give any other related product free on purchase of the mentioned product)															

13. SALES TO/BY MINORS*

(Yes =1, No = 0)	**Smoking tobacco**	**Smokeless tobacco**
a) Did you observe any sales assistants under the age of 18 years in the POS?		
b) If yes, what are their expected ages? Please list.		
c) Did you observe the vendor was selling any tobacco product to young people under the age of 18?		

If you observe sale of ST to/or by a potential minor please make an effort to ask their age for confirmation.

Appendix 5
POS vendor interview tool

(The products in the columns and their unit of sales will be revised after the inventory study)

**14. PRICES & Sale**		Cigarettes	Cigar/ Cigarillo	Beedhi	Hukaah/sheesha	Naswar	Gutka	Paan/ Betel Quid with tobacco	Tobacco Gum	Mawah	Zarda	Gul	Snus	Nasal Snuff	Khaini	Others
a) Unit price that customer pay (in local currency)																
b) Unit price that retailer pay																
c) Sales Tax (in local currency)																
d) Total number of units sold per day	By Quantity															
	By Weight															
e) f) How was the price obtained																
I. Cashier/ owner/ shop keeper																
II. Advertised price																

15. SUPPORT FOR VIABLE ALTERNATIVES

Why do you sell tobacco products? Tick all that apply. (Options should not be disclosed until the shopkeeper answer. Tick all that apply and then probe with the options remaining)	**Smoking tobacco products**	**Smokeless tobacco products**
a) Good profit margin		
b) Products are in demand		
c) Family business		
d) No other viable alternative		
e) Brings people into my shop to buy other things		
f) Other reasons (please state)		
Would you ever consider stop selling SLT? (Yes = 1, N0 = 2)		
If yes, for which of the following reasons? Tick all that apply (if No skip to 16)<br>(Options should not be disclosed until the shopkeeper answer. Tick all that apply and then probe with the options remaining)		
I. A reduction in profit margin		
II. A reduction in demand		
III. Stricter regulations and inspections		
IV. Limited supply		
V. A viable alternative		
VI. Awareness about its harms		
VII. Other reasons (please state)		

16. SUPPLIES

Where do you get your supplies? (Tick all that apply)	Cigarettes	Cigar/ Cigarillo	Beedhi	Hukaah/sheesh[illegible]	Naswar	Gutka	Paan/ Betel [illegible]	Tobacco Gum	Zarda	Mawah	Gul	Snus	Nasal Snuff	Khaini	Others
a) Wholesalers (Cash and carry)															
b) Manufacturers															
c) Importers															
d) Unknown source															
e) Another shop/ store															
f) Others (Please state)															

Why do you buy this product from a particular supplier? (Tick all that apply)	Cigarettes	Cigar/ Cigarillo	Beedhi	Hukaah/sheesha	Naswar	Gutka	Paan/ Betel Quid [illegible]	Tobacco Gum	Mawah	Zarda	Gul	Snus	Nasal Snuff	Khaini	Others
a) They are the only supplier															
b) They can supply the required amount															
c) They are cheap															
d) They are nearby															
e) They are a friend or family member															
f) They sell products or brands my customers want															
g) They give me incentives															
h) Another reason (please state)															

What is the nature of the incentive by a particular supplier? (Tick all that apply)	Cigarettes	Cigar/ Cigarillo	Beedhi	Hukaah/sheesha	Naswar	Gutka	Paan/ Betel Quid [illegible]	Tobacco Gum	Mawah	Zarda	Gul	Snus	Nasal Snuff	Khaini	Others
I. Discount															

II. Promotional Goods															
III. Point-of-sale displays															
IV. Free sample															
V. Others (please state)															

Where are your suppliers based (Tick all that apply)	Cigarettes	Cigar/ Cigarillo	Beedhi	Hukaah/sheesha	Naswar	Gutka	Paan/ Betel Quid with tobacco	Tobacco Gum	Mawah	Zarda	Gul	Snus	Nasal Snuff	Khaini	Others
a) Local															
b) Abroad															
c) If Abroad, Where?															
d) Do you have contact details of the supplier?															
e) If "d" Yes, Can you Provide the contact details															
f) If "e" Yes, Contact Details of suppliers	**(Type of Product)**														
	**(Type of Product)**														
	**(Type of Product)**														
	**(Type of Product)**														

17. TOBACCO ADVERTISING, PROMOTION, AND SPONSORSHIP

(Yes =1, No = 0)

How do you (retailer) try to retain your tobacco customers? Tick all that apply.	**Smoking tobacco**	**Smokeless tobacco**
a) Offer discounts		
b) Offer promotional goods		
c) Offer credit lines		
d) Good customer relationships		
e) Other ways (please state)		

18. SALES TO/BY MINORS

(Yes =1, No = 0)	**Smoking tobacco**	**Smokeless tobacco**
d) Do you have any sales assistants under the age of 18 years?		
e) If yes, what are their ages? Please list.		
f) Are they allowed to sell tobacco products?		
g) Do you ever sell any tobacco products to young people under the age of 18?		
h) If no, why not?		

**PROFIT**	Amount in local currency
a) Roughly what amount of profit do you make from daily sales (Overall)	
b) What amount of profit comes from daily cigarettes sale	
c) What amount of profit comes from daily smokeless tobacco sale	

19. EDUCATION AND PUBLIC AWARENESS

(Yes =1, No = 0)

Are you aware of any laws that impact your business of selling tobacco products?	**Smoking tobacco**	**Smokeless tobacco**
a) If yes, please state which law(s) and how it affects your business		
Are you visited by any inspector or regulator to inspect and/or test your tobacco products?	**Smoking tobacco**	**Smokeless tobacco**
a) Inspect products		
b) Test products		
c) Check your license or registration		
d) How many visits did you have in the last 3 years?		
e) What happens after the inspection?		
I. If found not in compliance, nothing happens		
II. If found not in compliance, there is a penalty		
III. If found in compliance, nothing happens		
IV. If found in compliance, you are given a certificate		
V. Any other consequences?		
Do you have to disclose the contents of smokeless tobacco products to any statutory body?		
Do you have to send the products to a laboratory, for testing after Inspection?		

Signature of the interviewer_________________________

Appendix 6

Details of in-country ethical approvals

S.No	Country	Name of the Ethics Committee	Date of approval	Reference of approval
1	Bangladesh	The Bangladesh Medical Research Council's National Research Ethics Committee	03/01/2019	BMCRC/NREC/2016-2019/961
2	India	Health Ministry's Screening Committee (HMSC)	13/03/2019	HMSC/2018-2675
3	Pakistan	National Bioethics Committee of Pakistan	28/10/2018	4-87/NBC-355/19/1695
