# Supplementary material for: Compliance of smokeless tobacco supply chain actors and products with tobacco control laws in Bangladesh, India and Pakistan: protocol for a multicentre sequential mixed-methods study
Source: BMJ Open. 2020 Jun 17;10(6):e036468. doi: 10.1136/bmjopen-2019-036468 (PMC7304837; doi:10.1136/bmjopen-2019-036468)
Supplement: Author's manuscript [file bmjopen-2019-036468.draft_revisions.pdf]

BMJ Open is committed to open peer review. As part of this commitment we make the peer review history of every article we publish publicly available.

When an article is published we post the peer reviewers' comments and the authors' responses online. We also post the versions of the paper that were used during peer review. These are the versions that the peer review comments apply to.

The versions of the paper that follow are the versions that were submitted during the peer review process. They are not the versions of record or the final published versions. They should not be cited or distributed as the published version of this manuscript.

BMJ Open is an open access journal and the full, final, typeset and author-corrected version of record of the manuscript is available on our site with no access controls, subscription charges or pay-per-view fees (http://bmjopen.bmj.com).

If you have any questions on BMJ Open's open peer review process please email info.bmjopen@bmj.com

BMJ Open

Compliance of smokeless tobacco vendors and supply chain actors with Tobacco Control laws: Protocol for a multi-centre sequential mixed-methods study.

Journal:	*BMJ Open*
Manuscript ID	bmjopen-2019-036468
Article Type:	Protocol
Date Submitted by the Author:	17-Dec-2019
Complete List of Authors:	khan, Zohaib; Khyber Medical University<br>Huque, Rumana; University of Dhaka, Economics<br>Sheikh, Aziz; University of Edinburgh, Division of Community Health Sciences<br>Readshaw, Anne; University of York Department of Health Sciences<br>Eckhardt, Jappe; University of York<br>Jackson, Cath; University of York, York Trials Unit<br>Kanaan, Mona; University of York, Health Sciences<br>Iqbal, Romaina; Aga Khan University, Department of Community Health Sciences and Medicine<br>Akhter, Zohaib; Aga Khan University<br>Garg, Suneela; Maulana Azad Med Coll, Community medicine<br>Singh, Mongjam; Maulana Azad Medical College<br>Ahmad, Fayaz; Khyber Medical University<br>Abdullah, S M; University of Dhaka Department of Economics<br>javaid, arshad; Khyber Medical University<br>A.Khan, Javaid; Aga Khan University, Department of Pulmonary medicine<br>Han, Lu; University of York Department of Health Sciences<br>Rahman, Aziz; Federation University Australia Faculty of Health<br>Siddiqi, Kamran; University of York, UK, Institute of Health Sciences
Keywords:	Health policy < HEALTH SERVICES ADMINISTRATION & MANAGEMENT, PUBLIC HEALTH, QUALITATIVE RESEARCH

SCHOLARONE™
Manuscripts

I, the Submitting Author has the right to grant and does grant on behalf of all authors of the Work (as defined in the below author licence), an exclusive licence and/or a non-exclusive licence for contributions from authors who are: i) UK Crown employees; ii) where BMJ has agreed a CC-BY licence shall apply, and/or iii) in accordance with the terms applicable for US Federal Government officers or employees acting as part of their official duties; on a worldwide, perpetual, irrevocable, royalty-free basis to BMJ Publishing Group Ltd ("BMJ") its licensees and where the relevant Journal is co-owned by BMJ to the co-owners of the Journal, to publish the Work in this journal and any other BMJ products and to exploit all rights, as set out in our licence.

The Submitting Author accepts and understands that any supply made under these terms is made by BMJ to the Submitting Author unless you are acting as an employee on behalf of your employer or a postgraduate student of an affiliated institution which is paying any applicable article publishing charge ("APC") for Open Access articles. Where the Submitting Author wishes to make the Work available on an Open Access basis (and intends to pay the relevant APC), the terms of reuse of such Open Access shall be governed by a Creative Commons licence – details of these licences and which Creative Commons licence will apply to this Work are set out in our licence referred to above.

Other than as permitted in any relevant BMJ Author's Self Archiving Policies, I confirm this Work has not been accepted for publication elsewhere, is not being considered for publication elsewhere and does not duplicate material already published. I confirm all authors consent to publication of this Work and authorise the granting of this licence.

For peer review only

Compliance of smokeless tobacco vendors and supply chain actors with Tobacco Control laws: Protocol for a multi-centre sequential mixed-methods study.

Khan Z[1], Huque R[2], Sheikh A[3], Readshaw A[4], Eckhardt J[5], Jackson C[4], Kanaan M[4], Iqbal R[6], Akhter Z[6], Garg S[7], Singh MM[7], Ahmad F[1], Abdullah SM[2], Javaid A[1], Khan J[6], Han L[4], Rahman MA[8], Siddiqi K[4].

Authors' affiliations:

[1]Khyber Medical University, Pakistan; [2]ARK Foundation, Dhaka, Bangladesh; [3]Usher Institute, University of Edinburgh, UK; [4] Department of Health Sciences, University of York, UK; [5]Department of Politics, University of York, UK; [6]Aga Khan Medical University, Pakistan; [7]Maulana Azad Medical College, New Delhi, India; [8] Federation University, Australia.

Corresponding author: Dr. Anne Readshaw

Email: anne.readshaw@york.ac.uk

For peer review only

Abstract

Introduction

South Asia is home to more than 300 million smokeless tobacco (ST) users. Bangladesh, India, and Pakistan as signatories to the Framework Convention for Tobacco Control (FCTC) have developed policies aimed at curbing the use of tobacco. The objective of this study is to assess the compliance of ST point-of-sale (POS) vendors and the supply chain with the articles of the FCTC and specifically with national tobacco control laws. We also aim to assess disparities in compliance with tobacco control laws between ST and smoked tobacco products.

Methods and Analysis

The study will be carried out at two sites each in Bangladesh, India, and Pakistan. We will conduct sequential mixed-method cross-sectional studies with multiple components including i. Mapping of ST POS, ii. Analyses of ST samples packaging, iii. Observation of POS, iv. Interviews with ST vendors at POS, and v. In-depth interviews with wholesale dealers/suppliers/manufacturers of ST. We aim to conduct a total of up to 900 interviews and POS observations, and 10-15 in-depth interviews across all three countries, using a random sampling technique. Data collection will be done by trained data collectors. Data will be entered and stored on a secure central database. Specific analysis plans will be used for each component of the study.

Ethics and dissemination

Ethical approvals for the study have been received from the Health Sciences Research Governance Committee at the University of York, UK. In-country approvals were from the National Bioethics Committee in Pakistan, the Bangladesh Medical Research Council, and the Indian Medical Research Council. Additionally, we will seek administrative approvals from

the relevant bodies at all study sites. Each randomly selected POS vendor will be provided with verbal and written information about the study, following which he/she will be asked to record his/her permission to be included in the study through signing an informed consent form. Where the POS vendor wants to be involved in the study, but is unwilling to sign any document, his/her verbal consent will be taken in the presence of an independent witness. Our results will be disseminated via scientific conferences, peer-reviewed research publications, and press releases.

Keywords: Smokeless tobacco, South Asia, point of sale, tobacco control, Bangladesh, India, Pakistan

Word Count: 3960

Strengths and limitations

- This will be the first multi-country study on ST vendors and supply chain actors in South Asia, where >85% of the world's ST users live.
- Our proposed study will develop/update the inventory of ST products available in the local markets of Bangladesh, India, and Pakistan. This will give us an insight into the scope of the products available in these markets, as well as information about illicit products.
- We will also map the ST POS at our study sites, thus providing us with baselines figures regarding the availability and density of ST POS, as well as geographical information such as proximity to schools etc. These can be used for future surveillance, and as a reference in the future assessment of compliance with the ST control laws.
- Although our focus is on ST, our study will also collect relevant data on smoking tobacco products, and identify any disparity that might exist regarding the implementation of tobacco control policies on smoking and ST products.
- It will be very difficult to collect tax-related and other monetary data from vendors.

Introduction

Smokeless tobacco (ST) refers to all tobacco products that are used without burning the product [1]. The International Agency for Research on Cancer estimates that ST contains more than 30 carcinogens [2]. The use of ST is associated with an increased incidence of head, neck and gastrointestinal cancers [3-5]. Additionally, ST use is associated with Oral Potentially Malignant Disorders (OPMDs) [6] and a variety of other oral and systemic diseases [7, 8]. An estimated 300 million people around the globe use ST products in some form, and the majority (≥85%) of these ST users live in South Asia [1, 9]. The use of ST products is particularly common in India, Bangladesh, and Pakistan, where it is considered an acceptable social norm [10]. This situation is further complicated by the presence of high amounts of Tobacco Specific Nitrosamines, the main cancer-causing agents in tobacco, in the products available in South Asia, compared to the rest of the world [11].

The Framework Convention for Tobacco Control (FCTC) is a global treaty that aims to enhance tobacco control measures and incorporate the provisions of the framework into their national tobacco control laws [12]. Although the FCTC is aimed at curbing all types of tobacco use, the focus of tobacco control in most countries has been on controlling smoking, with control of the non-smoking forms of tobacco often put on a back burner [13, 14]. The 2016 Global Progress Report on the implementation of FCTC highlighted gaps in the formulation

For peer review only

and implementation of ST control policies in most parties to the FCTC [15]. Bangladesh, India, and Pakistan are signatories to the FCTC and have made variable progress in implementing the articles of the framework by developing national tobacco control policies [16].

The tightening up of smoking regulations has been associated with a reported shift from smoking to ST use in some South Asian countries [9, 17]. The increase in ST use can be attributed to a variety of factors, including, but not limited to cultural acceptance, perceived safety of ST products compared to smoking, harm-reduction, low prices, and lax or absent ST control laws [14].

A 2018 report from the Global Knowledge Hub on Smokeless Tobacco indicated progress being made with regards to ST control policies and regulations in South Asia [14]. Many of these regulations and laws are aimed at demand reduction via various interventions at the tobacco points of sale [18, 19]. The success of these regulations and laws in curbing the tobacco epidemic in South Asia largely depends on the degree of compliance with the implementation of these laws and regulations [20]. However, the evidence on the implementation of the ST control laws and regulations at the POS in the context of India, Bangladesh, and Pakistan is scarce. Most of the previous work carried out in these countries focused on tobacco advertisement and promotion at the POS, with little emphasis on the other provisions of the FCTC, such as sale to/by minors, price and taxation, packaging and labeling, and provision of viable alternatives. As such, there is a need for a comprehensive assessment of the compliance of POS in India, Pakistan, and Bangladesh with the local ST control policies and the specific articles of the FCTC. `

This multi-country mixed methods study aims to provide a comprehensive assessment of compliance of ST POS and products with the tobacco control laws in our three target countries. Furthermore, we aim to elicit information on any disparities between the implementation of FCTC/local laws for ST and smoking. We will also develop a country-specific inventory of tobacco products, to gain insight into the diversity of the products available in the local market, as well as identifying illicit products. Additionally, we will assess the barriers and facilitators to compliance of the ST wholesale dealers and suppliers with the different provisions of the FCTC/local laws.

Methods and Analysis

Study design

We will conduct a sequential mixed-methods study, incorporating both qualitative (in-depth interviews with supply chain actors e.g. wholesale dealers, suppliers) and quantitative work (geo-mapping of ST POS, in-house analyses of ST products, POS observation and vendor interviews).

Study sites

We will conduct this study in Bangladesh, India and Pakistan. In each country, we have purposively selected two sites based on the use and diversity of the available ST products. In India, the study will be conducted in the North-East and North-West districts of Delhi. In Bangladesh, the study will be conducted in the districts of Dhaka and Rangpur. In Pakistan, the study will be conducted in the districts of Karachi and Peshawar.

For peer review only - http://bmjopen.bmj.com/site/about/guidelines.xhtml

Study duration

The data collection will be carried out from June 1, 2019, to May 30, 2020 and analyses will be completed by March 30, 2021.

General sampling strategy

We will use a multi-stage mixed sampling technique for the study (Fig. 1).

Stage 1: Within each of the selected administrative areas (District/Division), we will purposively select one predominantly urban and one predominantly rural sub-district.

Stage 2: We will randomly select three smaller administrative units per sub-district. These will be our Primary Sampling Units (PSU).

Stage 3: From each PSU, we will randomly select two enumeration blocks/neighbourhood areas/villages; these will be our Secondary Sampling Units (SSU).

Data collection (General considerations)

At each study site, we will deploy a team of trained data collectors to administer the quantitative data collection instruments (comprising the geo-mapping, in-house analysis of the ST products packaging, structured POS vendor interview, and POS observation components). Data collectors will be trained on how to administer the various study tools beforehand. They will also receive training in obtaining informed and written consent. Calibration of the product in-house analysis component will be done via video conferencing involving study teams from all sites. Data will be monitored for quality and completeness by the site leads/study leads/principal investigator. We will also conduct small pilot studies in each country to field-test our study tools and reduce interview time if deemed very long. As part of the piloting our study tools, we will carry out mock store observations and vendor interviews at POS not selected for our study. We will also conduct in-house mock product analysis.

Data management

Data will be initially collected on a paper-based questionnaire and then transferred to a central database kept on a secure server at the University of York. Each participant will be allotted a unique participant identification number and the recorded data will not contain any identifiable information about the participants. Only the study leads/principal investigator/study statistician will have access to the database and data collection tools. All filled questionnaires will be stored in locked filing cabinets at the collaborating institutions in each of the study centres.

In line with the General Data Protection Regulations (GDPR) of the European Union (EU) and the Research Governance Framework for Health and Social Care Research, data will be securely archived by the University of York for a minimum of five years after the completion of the study. Following authorisation from the sponsor, arrangements for confidential destruction will then be made. Recordings from focus group discussions (FGDs) will be deleted at the end of the study. If an individual withdraws consent for their data to be used, it will be destroyed without breach of confidentiality.

For peer review only

Study component-specific methods

POS Geo-Mapping

Eligibility criteria for POS

A POS will be eligible for inclusion in the study if it sells any form of ST. The common types of tobacco POS that might be eligible for our study will include:

a. General/departmental stores
b. Petrol pump/gas station stores
c. Beer or liquor stores
d. *Paan* (betel-quid) shops
e. Grocery stores
f. Exclusive tobacco shops
g. Discount shops
h. Mobile vendors/carts
i. Stationary carts
j. Country-specific additional POS types.

Identifying and mapping eligible POS

To identify the eligible POS, we will employ standard approaches for tobacco POS audits [21]. This will entail:

i. Developing a list of tobacco sellers in the selected areas by collecting the details of local vendors who have a license to sell tobacco products (if applicable), and/or the acquisition of general lists of local businesses/sellers from trade unions and/or local administrative authorities and combing potential tobacco POS from these lists.
ii. This will be followed by verifying the tobacco POS through "ground-truthing" by trained data collectors, who will visit these shops to ascertain, a. The existence of the POS, and b. The sale of ST products at these POS.
iii. Once ascertained, each eligible POS will be allotted a unique identification number and its location will be recorded via the Google Maps® digital application that is compatible with all the popular digital platforms e.g. Android, Apple iOS, and Windows. Once turned on, the application gives its users their current location on Google Maps, the Global Positioning System (GPS) coordinates, and the geolocation status.
iv. If no prior lists (license holders or businesses/sellers) are available at the study location(s), our data collection team will do "canvassing" of the ST POS by walking along all the primary and secondary roads in that area and identifying eligible ST POS (Appendix 1).
v. Additionally, we will request each ascertained POS vendor to identify other ST POS, which will be added to the list.
vi. If data collectors observe a mobile vendor during the ground-truthing or canvassing at the study sites, the area where the team establishes the first contact with that vendor will be marked as his/her location.

Measuring the density of ST POS

We will collect the most recent official population census data of our selected study sites from the concerned department(s) in each country. If such data are not available, we will use unofficial estimates gathered from the local administration/Upazila in-charge/village

For peer review only

chief/nazim/nambardar/ etc. The density of the POS will be calculated per 1,000 population at our study sites.

Measuring the distance of the POS from schools

We will acquire the list of all schools situated in the selected SSUs from the local education departments in each country. We will also request from the local education departments to provide us the GPS coordinates for the schools in the selected SSUs. We will plot all the ST POS and the schools on a digital map (Google Maps) and to assess if there is compliance of the local laws that ban the sale of tobacco products in the vicinity (distance will vary according to country) of the schools.

ST products in-house analysis

Eligibility criteria

All brands of ST products available in the markets at the study sites will be procured. In order for a product to be eligible, it must contain tobacco. The collection of the ST products will be based on the brand names and not the generic product e.g. all brands of *Gutkha* available in the local market will be collected. Loose products that are not sold in packaging will be eligible, but only one generic sample of the product will be procured. The most commonly sold brand/make of cigarettes, cigars, cigarillos, and beedi according to the selected vendor, will be procured.

Sample collection and storage

All ST sale shops/vendors identified in the selected SSUs by the geo-mapping exercise will be approached to procure the ST products that are available in the local markets. We will employ an adapted form of the unique packs sampling technique used for the TPackSS samples collection [22]. One of every unique pack of ST (any pack with at least one difference in an exterior feature of the pack including size, brand name, presentation, colours, cellophane, packaging material (i.e. hard, soft, tin), and the inclusion of a promotional item) available in the store will be purchased. This sampling will begin at a randomly-selected eligible vendor. After the purchase at the initial vendor, at each of the remaining eligible vendors, one of every ST pack that was not already purchased at a previous outlet will be purchased. If the selected vendor does not have any new packs (packs that were not purchased at another outlet), the sample collection team will move on to the next eligible vendor. Sample collection will continue until the team has approached the last eligible vendor. The sample collection team will ascertain the retail price of each procured pack from the vendor. They will also ascertain if the prices are inclusive of tax or not. The sample collection team will neither request nor accept a reduced price, in case the vendor offers a discount due to the large quantity being purchased, other than in those cases where discounted products are already being offered to all customers as a part of a promotion. All such general promotions, and individual “reduced price” offers will be recorded on a field notes sheet provided to each data collector.

Product analyses

All the procured samples will be sorted by product type, and each sample will be allotted a unique identification number marked on the Ziploc bag with indelible ink. The study team at each participating institution will analyze one sample at a time, carefully locking it back in the bag before moving onto the next sample. A physical inventory of all the products will be developed at each site and the data related to the products e.g. unique identification number, product family, purchase price and date of purchase will be fed into a digital inventory form (Appendix 2) adapted from the TPackSS inventory data entry form.

For peer review only

The in-house assessment of the ST products will involve assessment and recording of their size, price, tax disclosure, ingredient lists, health warnings, manufacturer details, etc. Our study tool (Appendix 3) has been adapted from the TAPS standard tool. We will collect the samples by brand names and assess whether disparities exist between ST product categories and/or different manufacturers. We will also collect data on the country of manufacture and analyze if differences in compliance to the relevant laws can be explained by differences in the country of manufacture and/or "smuggled/illicit" designation of the product. The definition of illicit products for each participating site will be developed after consulting local stakeholders. We will also analyze the collected cigarette packs, to assess their compliance with the local laws/FCTC. This will also help us in in the identification of any disparity in the local laws for ST and those for cigarettes. Data entry will be carried out through a web-based form that has been adapted from the TPackSS.

POS observation and Interview

Sample size

For the POS observation and Interviews, we calculated sample sizes for the individual countries based on the prevalence of non-compliance with specific provisions of the local tobacco control laws in the three countries. The point estimates were derived from published literature i.e. 20% of shops selling tobacco to minors in Pakistan [24], 19.4% POS non-compliant with COTPA regulations regarding sizing of the advertisements in India [25], and 20% POS non-compliant with tobacco advertisement and promotion laws in Bangladesh [26].

Using the formula sample $\boldsymbol{n} = \mathbf{[DEFF*Np(1\text{-}p)]/\ [(d^2/Z^2_{1-\alpha/2}*(N\text{-}1)+p*(1\text{-}p)],}$ and assuming a 20% prevalence of non-compliance, a confidence interval of 95%, and an absolute precision of 7%, the sample size for each country came out to be 251. After accounting for a 15% non-response (based on our ST supply chain study in Pakistan and Bangladesh) [23], the sample size was rounded off to 290 per country. All sample size calculations were carried out in Open Epi software [27].

Sampling strategy

We will follow the general sampling strategy (Fig 1). Once a sampling frame of eligible ST POS in each SSU has been developed through the geo-mapping, a random sample of the 13 POS vendors will be recruited from each SSU.

Recruitment

After providing brief information about the study to the person-in-charge of the POS at the time of the store observation, we would formally ask them to provide written consent to participate in both the POS observation and POS vendor interview components of the study. Those who do not agree to participate after receiving the study information will not be enrolled in the study. The reasons for declining to participate in the study will be recorded. Following consent, the field worker will proceed to data collection. The consent provider, which could be either one of the POS owner (not land-lords), POS manager, or sales assistant(s) will be eligible for interview.

Data collection and tools

An adapted version (Appendix 4) of the Standardized Tobacco Assessment for Retail Settings (STARS) tool [28], will be used to record the observations about product promotion, advertisement, and health warnings, etc. We will observe both the exterior and interior of the shops to assess the compliance with FCTC/local laws. The tool consists of data fields on health warnings inside and outside the shop, product advertisement, promotions, display shelves,

For peer review only

dummy packs, product placement, products sold as confectioneries, and cross-promotion. We will also collect similar data for cigarettes and other smoking products, for comparison.

Our interview tool (Appendix 5) has been adapted from a survey instrument that has already been field-tested in the UK, Bangladesh, Nepal, and Pakistan [23]. The tool will be translated into local languages at each study site. In addition to a series of close-ended questions on ST products regarding their supply, sale, popularity, prices, profits, and taxes, the tool also includes similar questions for cigarettes, to allow for comparative analysis. We will collect data on mediating/moderating variables that could act as barriers/facilitators to the increased implementation of the FCTC/local laws. These variables include awareness of local tobacco control legislation, standards, and obligations, awareness of adverse health effects of ST, the effect of health warnings on sales, market share of SLT products, overall sales related to availability of tobacco products for non-exclusive vendors, incentives by manufacturers/suppliers, profit margins, regular inspections, penalties for non-compliance, and availability of viable alternatives.

Data analysis

The main statistical analysis will report the frequencies and proportions of shops that comply with the WHO FCTC and local tobacco control policies, as measured by the different items on the survey questionnaire and provide a 95% confidence interval of this estimate. We will also provide summaries for the other categorical variables using frequencies and percentages and for continuous variables, we will provide means and standard deviations in addition to graphical representation. Medians and inter-quartile ranges will also be computed if the data are skewed.

We will use logistic regression to explore the bivariate association of compliance with shop characteristics, such as the type of shop, adjusting for the study design. We will further expand these regression models to explore the association of compliance with multiple explanatory variables adjusting for the study design. Where possible, figures related to ST sales will be compared with those of cigarette sales, using similar approaches to the above. We will consider a *p*-value of less than 0.05 to be statistically significant. A detailed statistical analysis plan will be developed prior to the main analysis, which will include any *a priori* subgroup analyses and strategies for handling missing data. We will use STATA 15 for analysis.

In-depth Interviews (Supply chain)

Participants, sampling and recruitment

At each study site, we will identify at least five supply chain actors (from manufacturers, wholesalers, suppliers) with the help of the POS survey respondents. These actors will then be approached to participate in our study.

Data collection

A topic guide will be developed for the interviews. The contents of the guide will be based on the findings from the POS survey. That is, the responses in the survey that warrant further exploration will be identified by the study teams in each country and the topic guide aimed at eliciting further information on the responses will be developed by the qualitative research team.

The interviews will be conducted at locations that are easily accessible to participants, such as their workplaces or the offices of ward/union council office bearers or the village chief's house/hujra. Each interview will last 30-60 minutes. A trained moderator will conduct the interviews in the local language and will be recorded digitally.

For peer review only

Data analysis

The interviews will be transcribed verbatim, translated into English and analysed using deductive content analysis. A categorisation matrix will be developed and organised to address the study objective. The data from the three countries will be coded using the same matrix. Excel software will aid data handling.

Ethics and Dissemination

We will uphold the core principles of good ethical practice i.e. autonomy, justice, beneficence, and non-maleficence during our research study. We will adhere to the fundamental principles of human rights as per the Helsinki Declaration. Ethical approval has been obtained from the University of York "Health Sciences Research Governance Committee". Additionally, each participating South Asian institution in ASTRA has obtained ethical approvals from their institutional/national ethics bodies. All potential participants will be provided with information regarding the project and written/verbal consent will be taken from each participant prior to recruitment. Consent forms will contain personal details including name, date of birth, and address.

All information collected during the course of the study will be kept strictly confidential. There will be restricted access and disposal arrangements for the participants' personal details. Information will be held securely on paper/electronically at the University of York. The researchers will also comply with all aspects of the UK's Data Protection Act 2018. Data (other than consent forms) transferred to or from the University of York will be coded with a study enrolment number and will not include participants' identifiers. We have studied the conditions under which disclosures to law enforcement agencies are required and are satisfied that the chances of these occurring (criminal activities) are highly unlikely.

Our study team will make sure that concerns regarding sensitive questions such as tax payment, illicit trade, etc. are adequately addressed, by taking the respondents into confidence. In our experience that can be done by reassuring the respondents that the study is part of a university/academic project and that their responses will be kept confidential and will not be passed on to law enforcing agencies. They are then usually more open to discussing sensitive issues. The purpose of this study is to provide a sense of the disparity between regulatory policies and practices in ST control. Without ensuring our participants' complete protection from any litigation following disclosures, we would not be able to provide valid study findings.

To engage our academic audience and the research fraternity, we will publish the results of the study in peer-reviewed journals. We will also present our findings at an international public health conference and an international tobacco control conference. We will also publish an abstract of our findings on the websites of all the partner institutions. We will also present our work to regional research meetings. Additionally, we will incorporate the learning from this project into academic lectures for Master students of public health. We have already established national stakeholders groups in all three target countries. These comprise researchers, policymakers, and representatives of Tobacco Control Cells, representatives of health and education departments, the WHO, school owners, and community members. At all stages of the project, we will carry out meetings/workshops in collaboration with the National Tobacco Control Cell. We will invite the national stakeholders, other Non-Governmental Organisations, community leaders, and community members to participate in these workshops.

For peer review only

Declarations

The authors declare no conflict of interest.

Patient and public involvement

The study is supported by stakeholder groups in each participating country. The stakeholder groups consist of health professionals, policy-makers, representatives from the general society, students and influencers. The stakeholder groups meet once every year, and so far have been involved in the design of the study tools, refinement of research questions and data collection methods. These group will also be involved in the dissemination of findings form the study.

Funding

This research is funded by the UK's National Institute for Health Research (NIHR) [ASTRA (Grant Reference Number 17/63/76)]. The views expressed in this publication are those of the authors and not necessarily of the NIHR or the Department of Health and Social Care.

Authors contribution

ZK, KS, RH, SG, MMS conceived the study, wrote the first and all subsequent drafts.

AR, FA, AR, ZA, AS, RI, AJ, JK and AS wrote the initial drafts and commented on the final draft.

MK and LH commented on all drafts and wrote up the sampling and analyses section.

JE and CJ commented on initial and final versions of the draft and wrote up the section on in-depth interviews.

References

1. Eriksen M, Mackay J, Ross H. The tobacco atlas. American Cancer Society; 2013.

2. IARC Working Group on the Evaluation of Carcinogenic Risks to Humans, World Health Organization, International Agency for Research on Cancer. Smokeless tobacco and some tobacco-specific N-nitrosamines: World Health Organization; 2007.

3. Guha N, Warnakulasuriya S, Vlaanderen J, Straif K. Betel quid chewing and the risk of oral and oropharyngeal cancers: A meta-analysis with implications for cancer control. International Journal of Cancer. 2014;135(6):1433-1443.

4. Boffetta P, Hecht S, Gray N, Gupta P, Straif K. Smokeless tobacco and cancer. Lancet Oncol. 2008;9(7):667-675.

For peer review only

5. Khan Z, Dreger S, Shah SMH, Pohlabeln H, Khan S, Ullah Z, Rehman B, Zeeb H. Oral cancer via the bargain bin: The risk of oral cancer associated with a smokeless tobacco product (Naswar). PloS one. 2017;12(7):e0180445.

6. Khan Z, Khan S, Christianson L, Rehman S, Ekwunife O, Samkange-Zeeb F. Smokeless tobacco and oral potentially malignant disorders in South Asia: a systematic review and meta-analysis. Nicotine and Tobacco Research. 2017;20(1):12-21.

7. Siddiqi K, Shah S, Abbas SM, Vidyasagaran A, Jawad M, Dogar O, Sheikh A. Global burden of disease due to smokeless tobacco consumption in adults: analysis of data from 113 countries. BMC Med. 2015;13:194-015-0424-2.

8. Sinha DN, Abdulkader RS, Gupta PC. Smokeless tobacco-associated cancers: A systematic review and meta-analysis of Indian studies. International Journal of Cancer. 2016;138(6):1368-1379.

9. Suliankatchi RA, Sinha DN, Rath R, Aryal KK, Zaman MM, Gupta PC, Karki KB, Venugopal D. Smokeless Tobacco Use is “Replacing” the Smoking Epidemic in the South-East Asia Region. Nicotine Tobacco Res. 2017;1:6.

10. Gupta PC, Ray CS. Smokeless tobacco and health in India and South Asia. Respirology. 2003;8(4):419-431.

11. Stanfill SB, Connolly GN, Zhang L, Jia LT, Henningfield JE, Richter P, Lawler TS, Ayo-Yusuf OA, Ashley DL, Watson CH. Global surveillance of oral tobacco products: total nicotine, unionised nicotine and tobacco-specific N-nitrosamines. Tob Control. 2011;20(3):e2.

12. Shibuya K, Ciecierski C, Guindon E, Bettcher DW, Evans DB, Murray CJ, WHO Framework Convention on Tobacco Control. WHO Framework Convention on Tobacco Control: development of an evidence based global public health treaty. BMJ. 2003;327(7407):154-157.

13. WHO Study Group on Tobacco Product Regulation. Advisory note: Waterpipe tobacco smoking: health effects, research needs and recommended actions for regulators. 2015.

14. Zaatari GS MD, Bazzi A. Impact of the WHO FCTC on non-cigarette tobacco products. Tob Control. 2018.

15. Chung-Hall J, Craig L, Gravely S, Sansone N, Fong GT. Impact of the WHO FCTC over the first decade: a global evidence review prepared for the Impact Assessment Expert Group. Tob Control. 2018.

16. Shang C, Lee HM, Chaloupka FJ, Fong GT, Thompson M, O'Connor RJ. Association between tax structure and cigarette consumption: findings from the International Tobacco Control Policy Evaluation (ITC) Project. Tob Control. 2018.

17. Khan Z. A gaping gap (smokeless tobacco control in Pakistan). Tobacco induced diseases. 2016;14(1):36.

For peer review only

18. World Health Organization. Monitoring tobacco control among youth in countries of the South-East Asia Region: 2018. 2018.

19. World Health Organization. Second joint mission of the United Nations Interagency Task Force on the Prevention and Control of Noncommunicable Diseases, Sri Lanka, 23-27 April 2018. 2018.

20. He Y, Shang C, Huang J, Cheng KW, Chaloupka FJ. Global evidence on the effect of point-of-sale display bans on smoking prevalence. Tob Control. 2018.

21. Feld AL, Johnson TO, Byerly KW, Ribisl KM. How to Conduct Store Observations of Tobacco Marketing and Products. Prev Chronic Dis. 2016;13: E25.

22. Smith K, Washington C, Brown J, Vadnais A, Kroart L, Ferguson J, Cohen J. The Tobacco Pack Surveillance System: A Protocol for Assessing Health Warning Compliance, Design Features, and Appeals of Tobacco Packs Sold in Low- and Middle-Income Countries. JMIR Public Health Surveill. 2015;1(2):e8.

23. Siddiqi K, Scammell K, Huque R, Khan A, Baral S, Ali S, Watt I. Smokeless Tobacco Supply Chain in South Asia: A Comparative Analysis Using the WHO Framework Convention on Tobacco Control. Nicotine Tob Res. 2016;18(4):424-430.

24. CTC Pakistan. Stubbing it out. In Edited by Anonymous Pakistan: Society for Alternative Media and Research; 2017:[Anonymous].

25. Patel S, Rendell H, Maudgal S, Oswal K. Tobacco industry tactics with advertisements at the point of sale in Mumbai. Indian J Cancer. 2013;50(3):245-249.

26. Bhuiyan MEH, Ovi FH, Alam MM, Farheen I. Documentation on Tobacco Advertising, Promotion & Sponsorship in Bangladesh. 2014;ISBN: 978-984-33-6739-6.

27. Dean A, Sullivan K, Soe M. OpenEpi: Open source epidemiologic statistics for public health, version. 2014.

28. Henriksen L, Ribisl KM, Rogers T, Moreland-Russell S, Barker DM, Sarris Esquivel N, Loomis B, Crew E, Combs T. Standardized Tobacco Assessment for Retail Settings (STARS): dissemination and implementation research. Tob Control. 2016;25(Suppl 1):i67-i74.

For peer review only

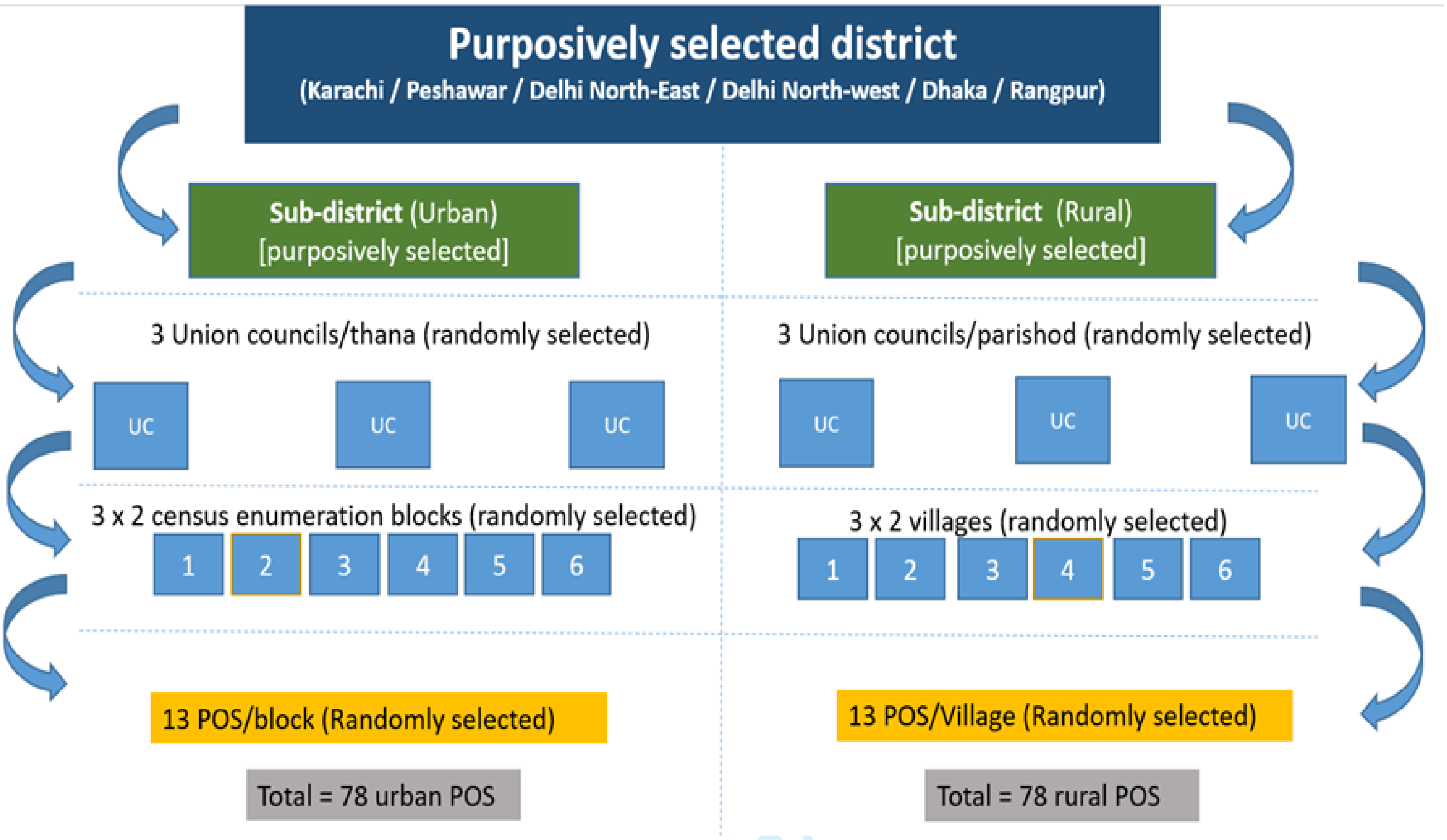

Figure 1. General sampling strategy for all study sites

Appendices

1. Field Protocol for Data Collection
2. Product Inventory Form
3. Product In-house assessment tool
4. Point of sale observation tool
5. Point of sale Interview tool

For peer review only

Appendix 1

ASTRA Sellers Study Field Protocol

After selection of villages and enumeration blocks in rural and urban areas respectively through ASTRA sellers study sampling strategy, data collectors will be sent to these villages and enumeration blocks for mapping of the POS and collection of samples for In-House assessment. Following the is the field protocol for Mapping and Sample collection.

Inclusion criteria for POS

A POS will be eligible for inclusion in the study if it sells any form of Smokeless Tobacco. The common types of tobacco POS that are eligible for our study include:

a. General/departmental stores
b. Petrol pump/gas station stores
c. Beer or liquor stores
d. *Paan* (betel-quid) shops
e. Grocery stores
f. Exclusive tobacco shops
g. Discount shops
h. Mobile vendors/carts
i. Stationary carts
j. Any Other ________________. (Country teams can add specific POS not in the list)

Walking Protocol

The data collector will observe the following guidelines to map the POS in villages and enumeration blocks.

A. *When Map of the Roads is available*

1. Obtain the map of the specific area (Village/enumeration block) with all the primary roads marked on it.
2. Mark the geographic boundaries of that village/enumeration block on the map.
3. Number all the roads for identification and ease e.g. 1,2,….n
4. Identify the start point of each road as **"A"** and end point as **"B"**.
5. Start from point **A** of the road No.1, and map all the POS available on both sides of the road until you reach point **B** of the road.
6. Repeat the process until all the roads are covered.
7. While mapping the POS at each road ask the shopkeepers for POS away from the roads. Visit those POS and map them.

B. *When Map of the Roads is not available*

For peer review only

1. Identify the geographic boundaries of the village or enumeration block on the map.
2. Identify and mark all the primary roads on the map with the help of key informants.
3. Number all the roads for identification and ease e.g. 1,2,….n
4. Identify the start point of each road as **"A"** and end point as **"B"**.
5. Start from point **A** of the road No.1, and map all the POS available on both sides of the road until you reach point **B** of the road.
6. Repeat the process until all the roads are covered.
7. While mapping the POS at each road ask the shopkeepers for POS away from the roads. Visit those POS and map them.

Notes:

- In case if the shop is closed, and you suspect that it as a ST POS, map it by standing at the door of the shop. Mention in the comments as "closed".
- In case of mobile vendor/carts, map at the location where you find them.

For peer review only

Mapping of POS

For mapping use **Google Maps** application, it is usually present in the phone but if not download from play store.

1. Open **Google Maps** from your phone. The following screen will appear. Your location should be **On** and your phone must have access to the internet via mobile data or Wi-Fi.

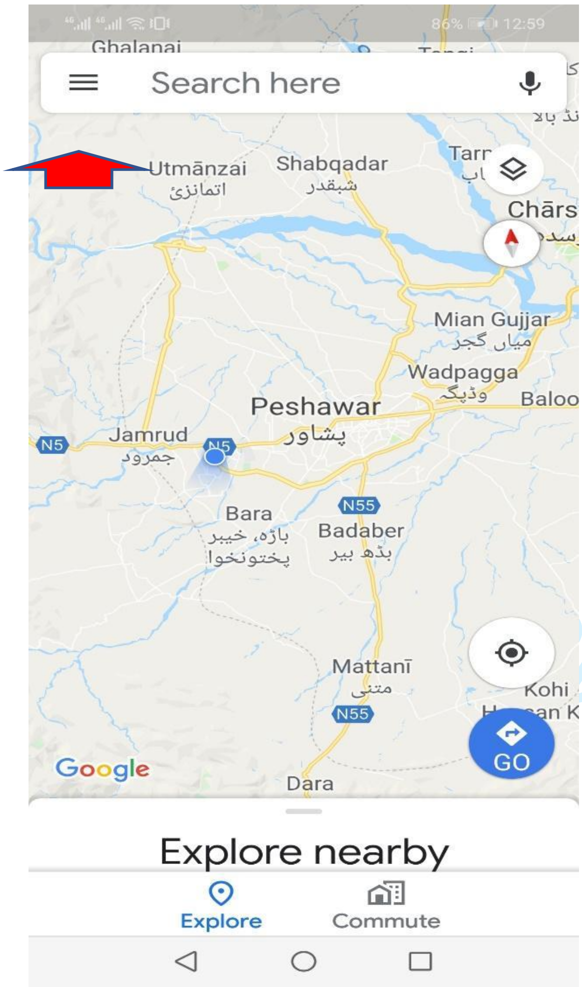

2. Touch the three horizontal lines on the top left corner of the screen beside the search bar. A sidebar will open showing different options.

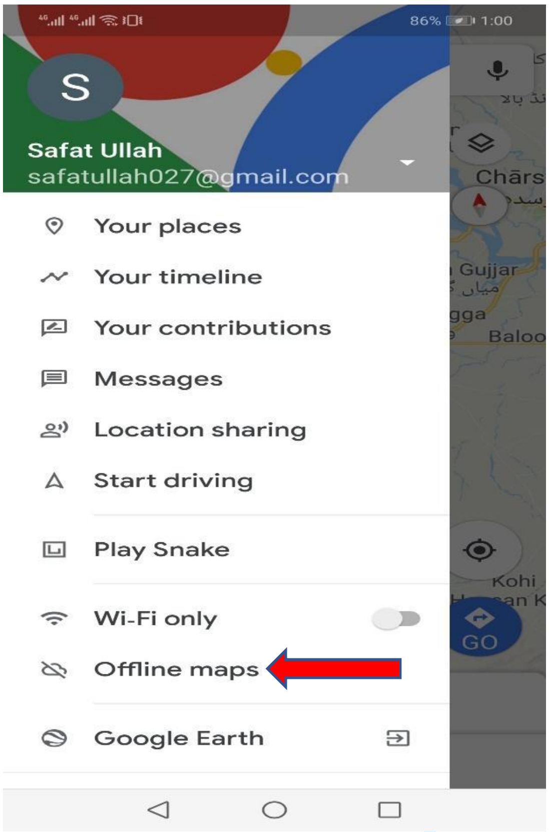

3. Go to the offline maps in the sidebar. A new window will pop up.

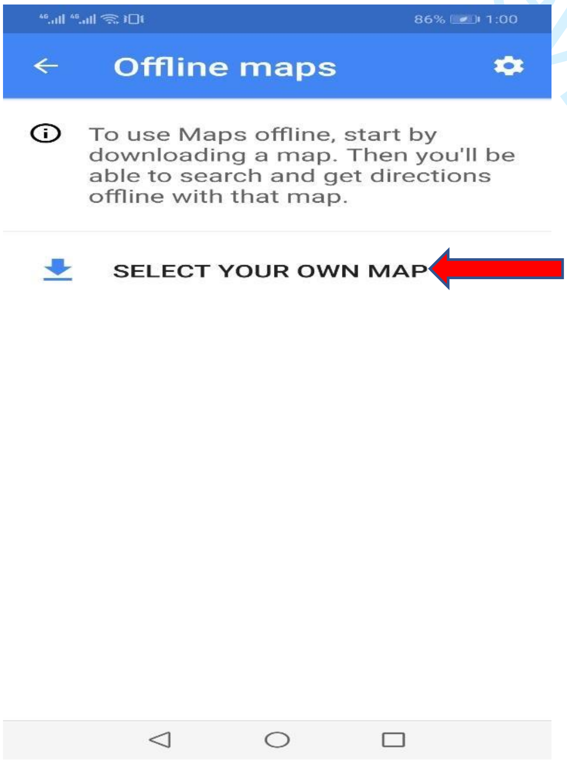

4. Click on "**Select your own Map**". Next window will open showing you a map with the highlighted area in a rectangle. You can swipe through to move to another area and zoom in or out.

review only

For peer review only - http://bmjopen.bmj.com/site/about/guidelines.xhtml

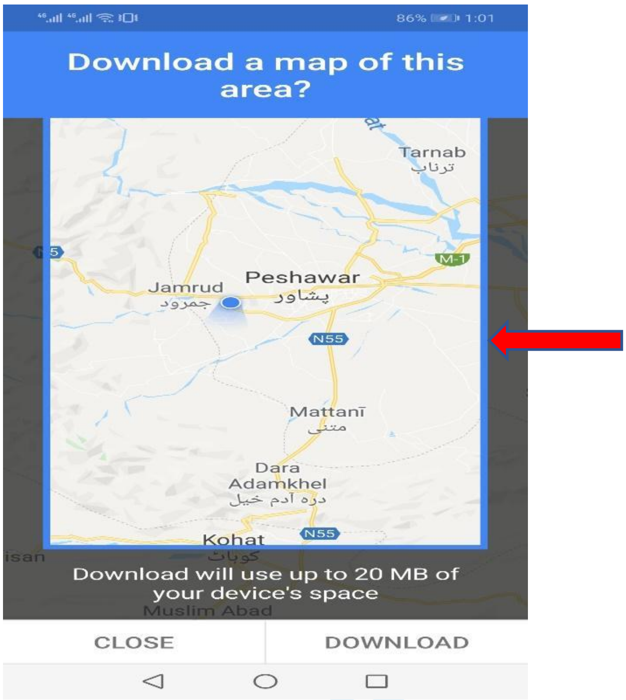

5. Select the area for which you want to download the map. (for example, I have selected Hayatabad). You can download maps for different locations separately.

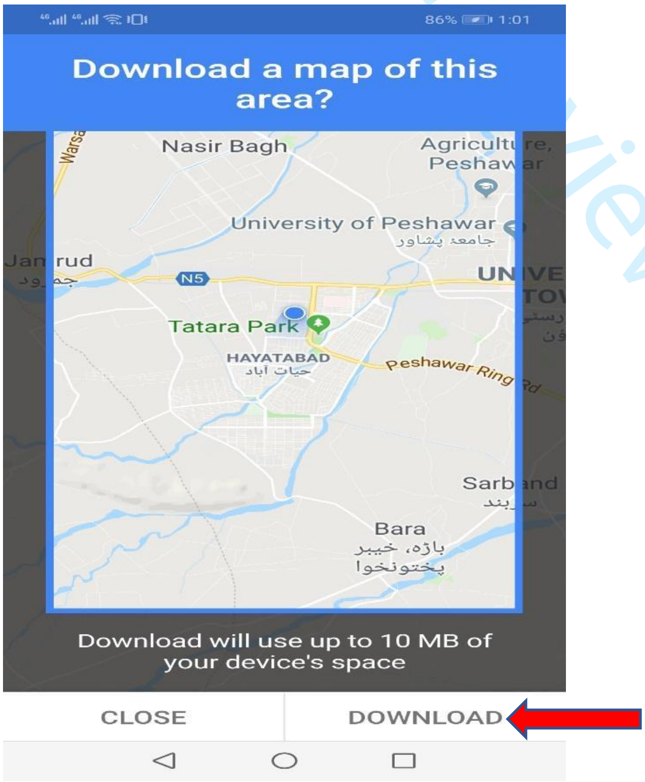

6. Clicking on the **download** will start downloading the map of that area. (this map will work for 30 days). You can update or rename or delete the map.

For peer review only

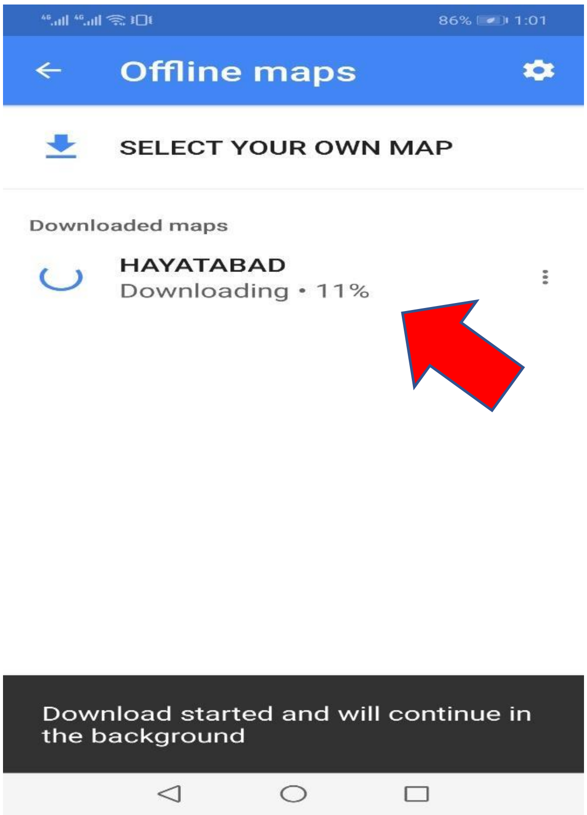

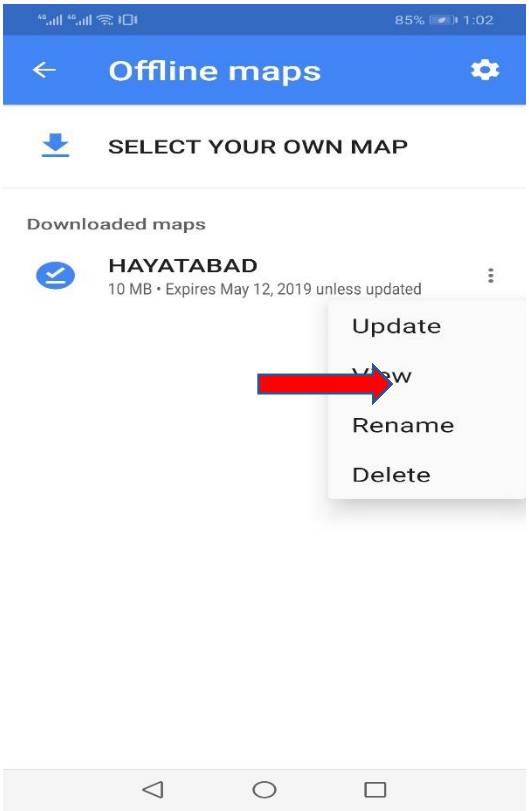

7. When you access the area where there is no internet, your location must be on. (GPS works offline as well).

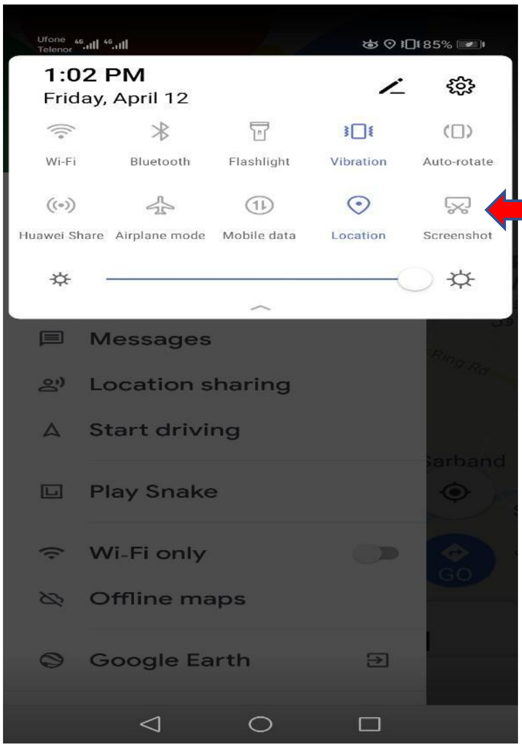

8. Reaching at the first shop open your offline map for the area by clicking in the highlighted map.

For peer review only

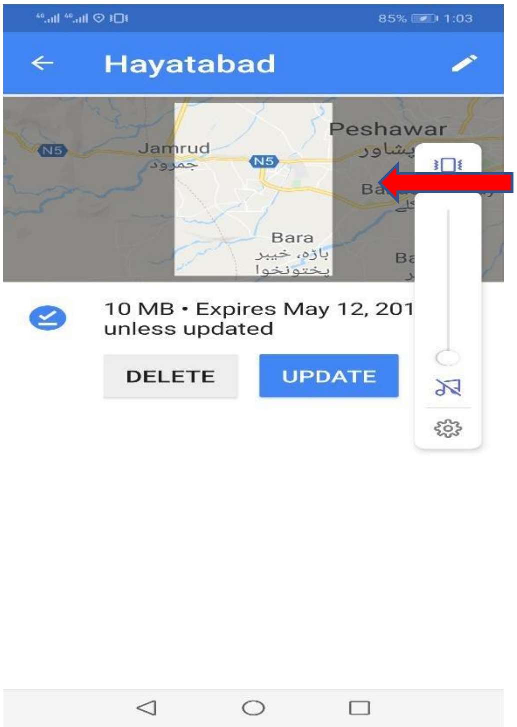

9. Click on **My current location** in the lower right corner above the Go option.

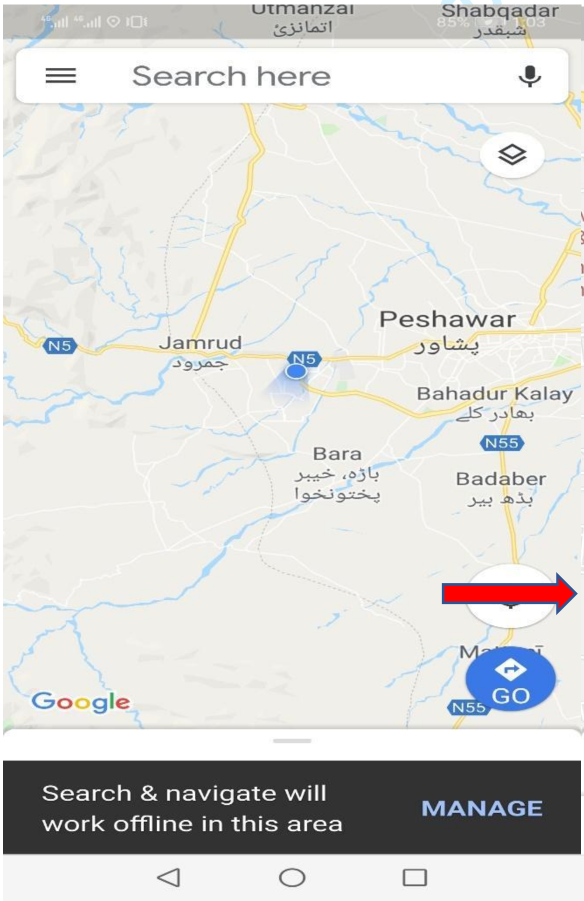

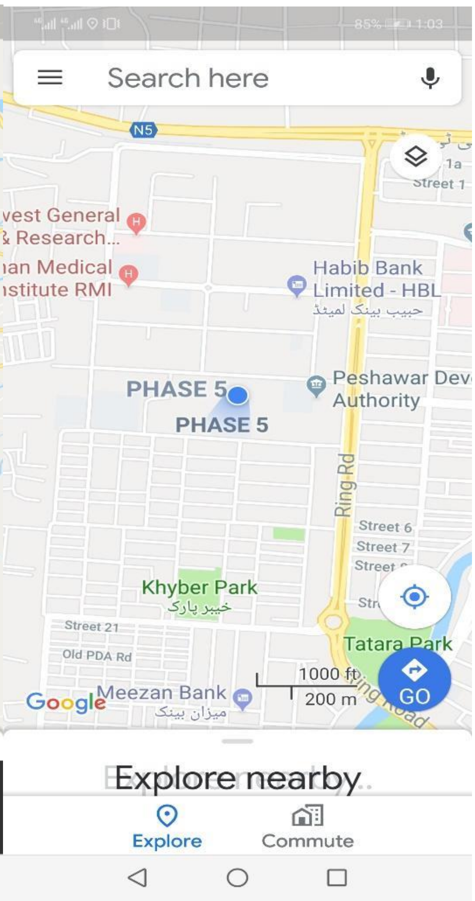

10. Touch and hold on screen (the exact location is shown via GPS) for three seconds. A red pin will pop up on the map showing coordinates at the lower screen.

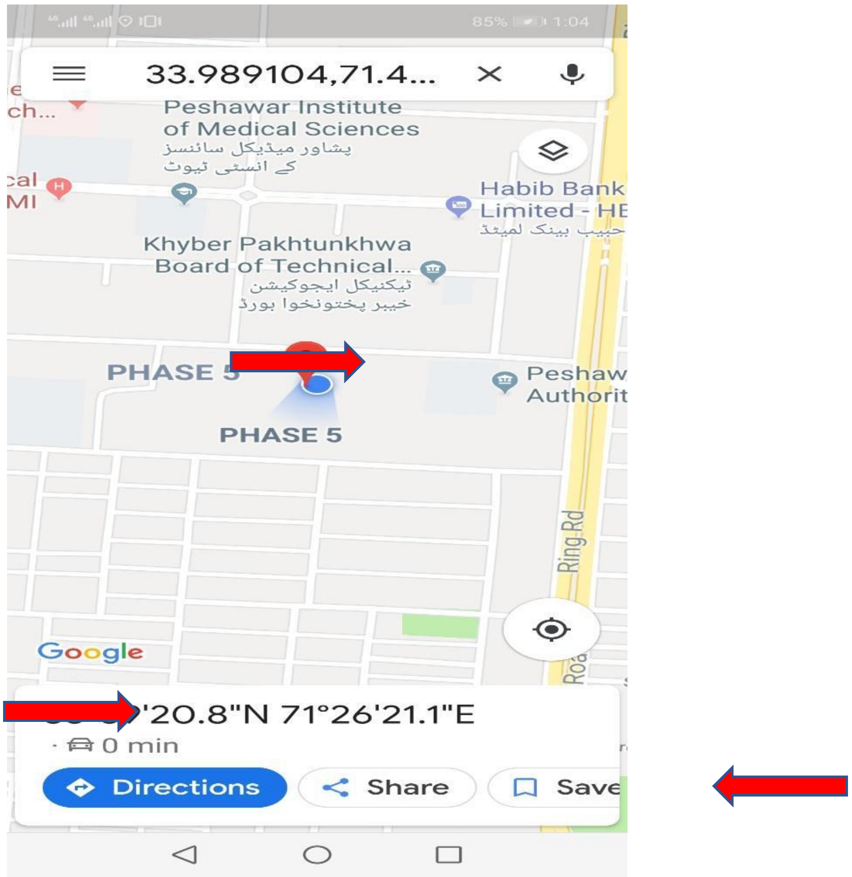

11. Below the coordinates, there is a **save** option and by clicking the save options a new window will popup showing different list options. Create a **new list** with the name of the area.

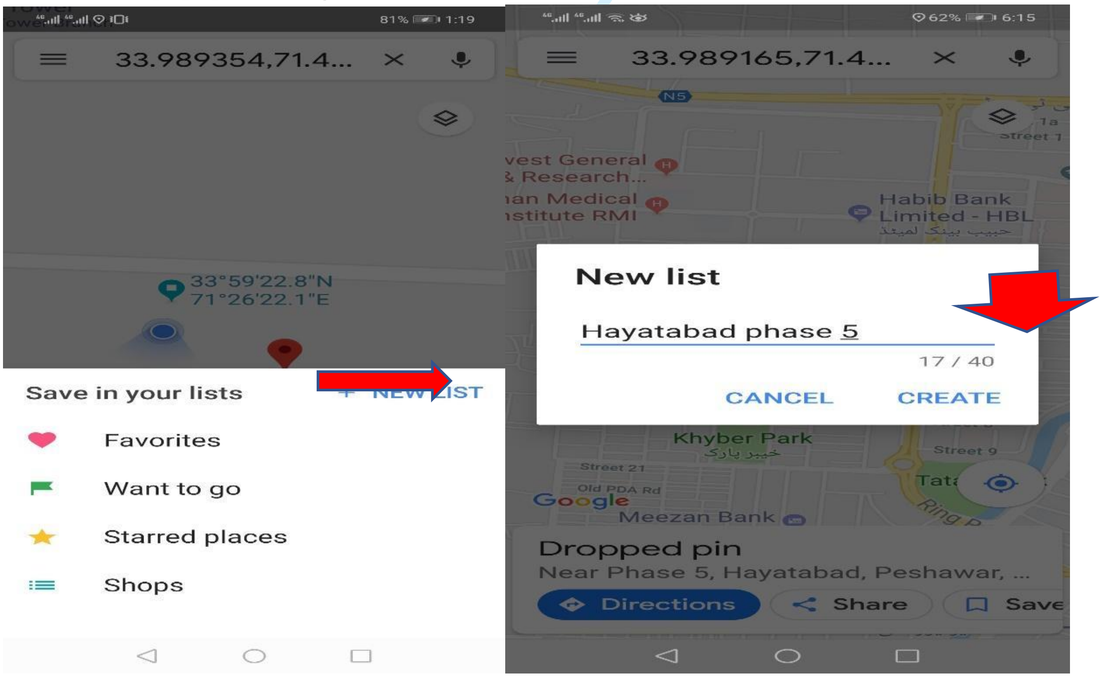

12. Open the list from the Horizontal lines on the main screen by opening **Your Places** and then **Saved**.

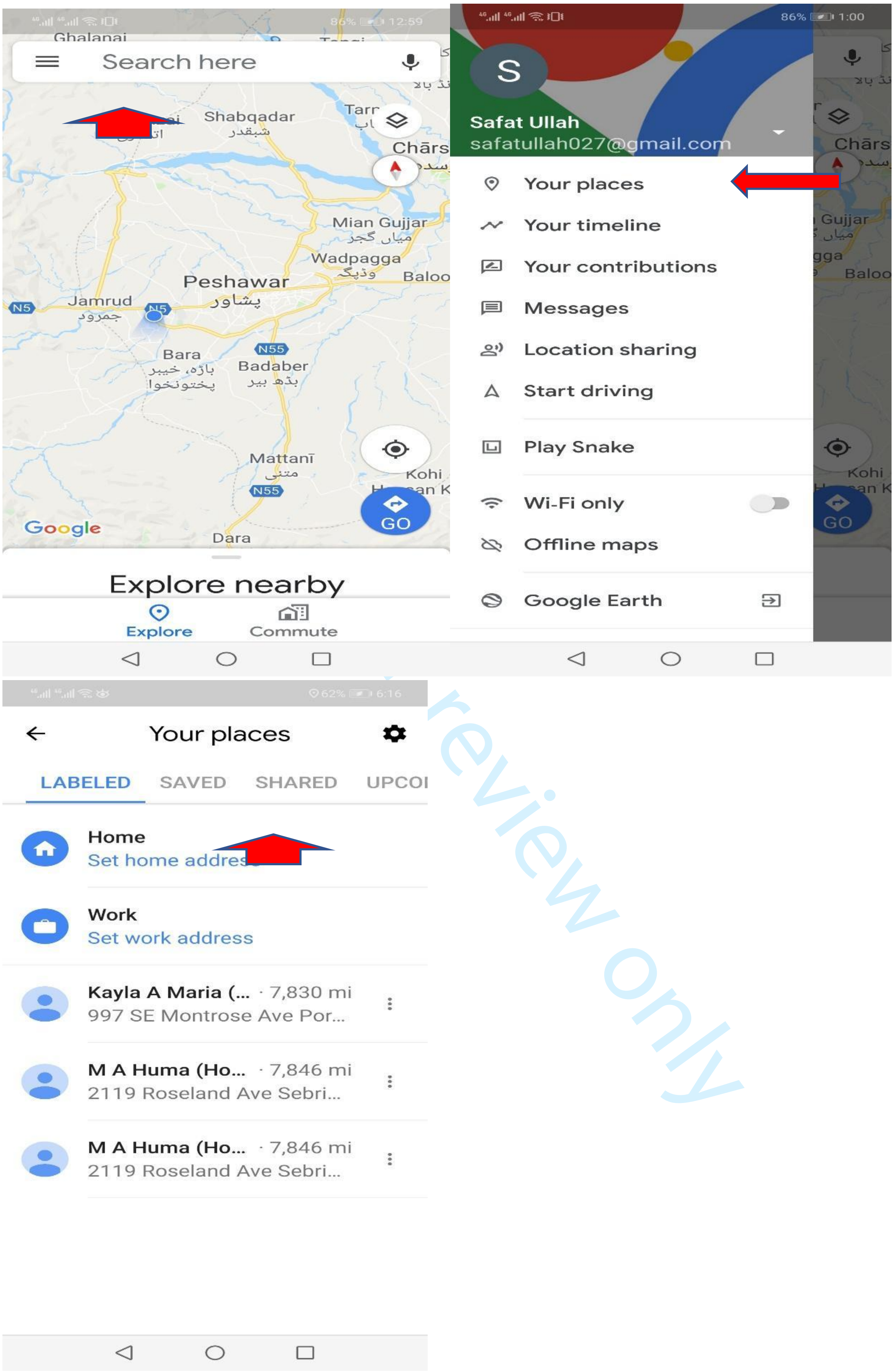

13. Opening the list, you will see the **Dropped pin**. To the right of the dropped pin, you will see **three dots**. By clicking these dots, click **add comments** and in comments put the serial number, name (shop name or owner name) and address of the shop (an area or market name).

For peer review only - http://bmjopen.bmj.com/site/about/guidelines.xhtml

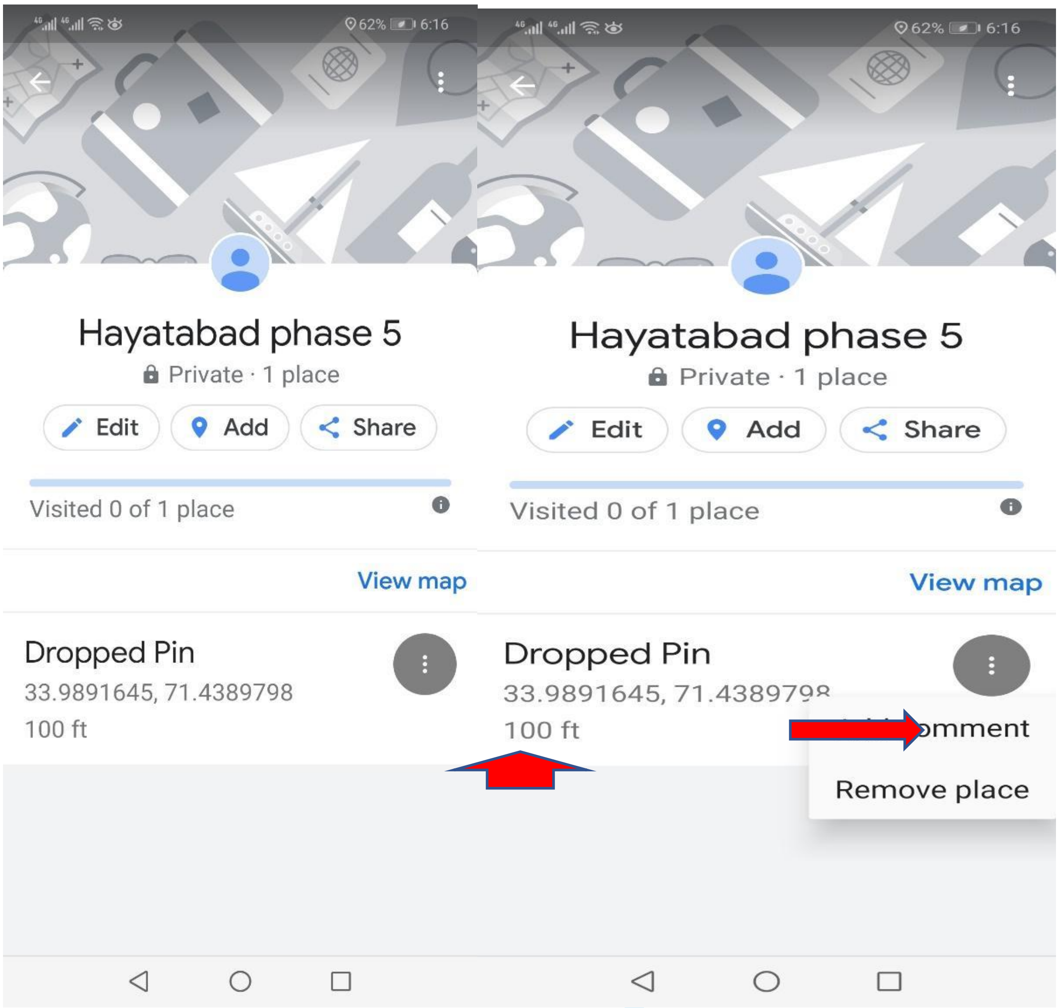
62% 6:16
Hayatabad phase 5
Private · 1 place
Edit
Add
Share
Visited 0 of 1 place
View map
Dropped Pin
33.9891645, 71.4389798
100 ft
62% 6:16
Hayatabad phase 5
Private · 1 place
Edit
Add
Share
Visited 0 of 1 place
View map
Dropped Pin
100 ft
Remove place

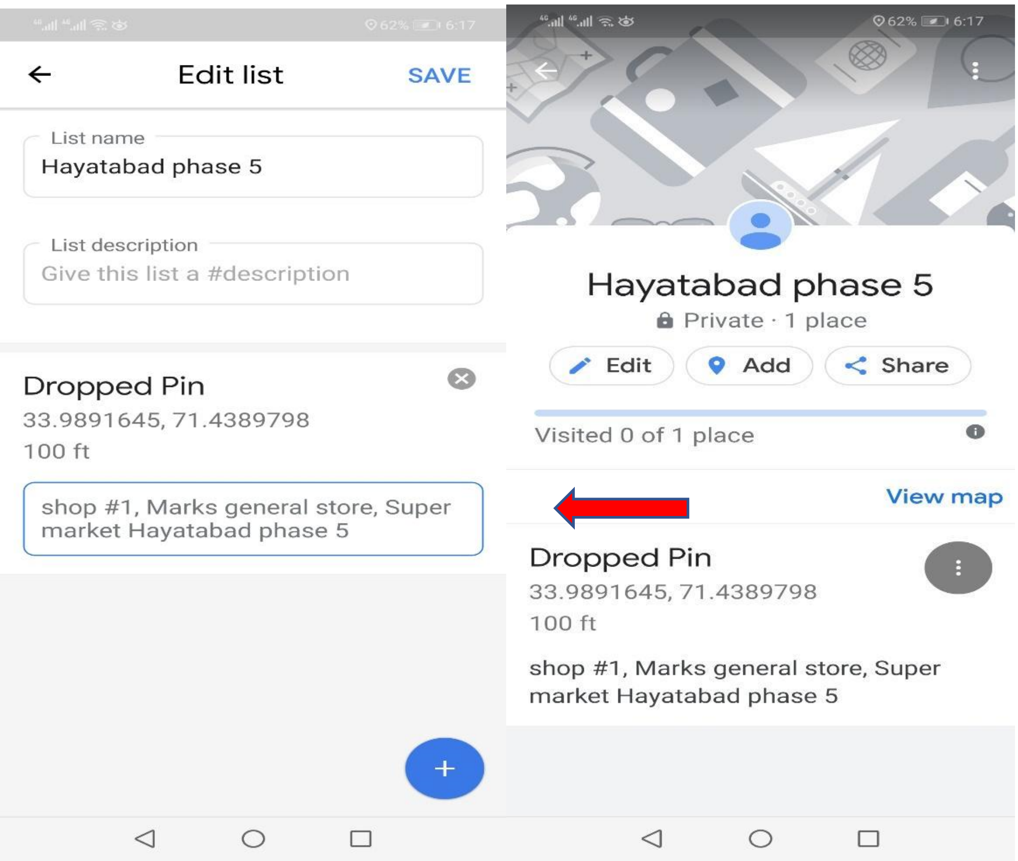

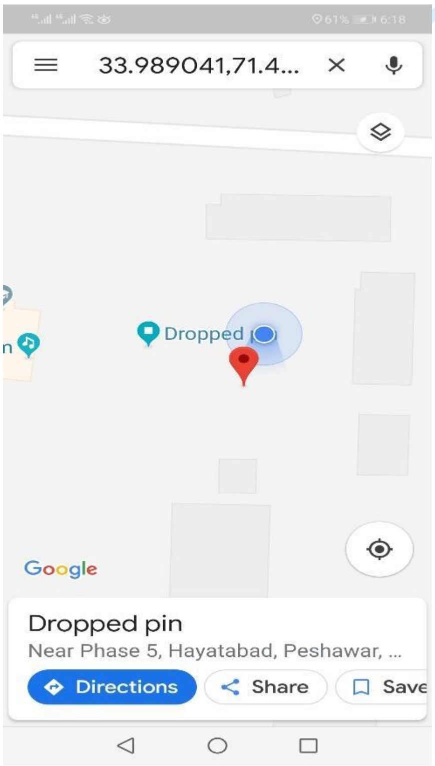

14. Similarly, drop pins of all the POS you visit.
15. This method works for **Online** (with access to the internet) and **Offline** (Without access to the internet),

Create a Timeline

review only

When you turn on Location History, your Timeline will reflect the places you have gone with the devices in which your account is logged-in and which are reporting location.

Open Timeline:

1. On your Android phone or tablet, open the Google Maps app .
2. Tap Menu > Timeline .
3. Tap More > Settings.
4. Make sure you see "Location is on." If you don’t, tap Location is off > turn on Location.
5. Make sure you see "Location History is on." If you don’t, tap Location History is off > turn on Location History.

For peer review only

Purchase of Products

Data collectors will be sent in pairs to map the POS. Arriving at the first shop one data collector will purchase the products and another one will map the shop;

1. The purchaser will visit the counter and ask for all the available brands of following smokeless tobacco (ST) products.
    a) Naswar
    b) Paan/ Betel Quid
    c) Gutka
    d) Tobacco Gum
    e) Gul
    f) Snus
    g) Zarda
    h) Nasal Snuff
    i) Khaini
    j) Others___________(Country Specific)

Complete **unique packs sample** by purchasing all unique smokeless tobacco packs available.
Unique Pack Definition: any pack with at least one difference in an exterior feature of the pack including: size, brand name, presentation, colors, packaging material (i.e. hard, soft, tin), and inclusion of a promotional item.

a. Face the counter and request to purchase one of each unique ST pack.
b. Capture the price of each pack. Place a sticker on the back of the pack and record the price on the sticker.
    i. For retailers where the price is not given, do not request nor accept a reduced price if the vendor offers a discount due to the large quantity being purchased.
c. Ask the shopkeeper to confirm that one of each ST pack they sell has been acquired and purchase any additional packs identified.
d. Also acquire one of the most commonly sold cigarette pack. Put a price tag on the pack. If cigarette packs is not available with the shop, continue with the process.
e. Place the packs in a zip lock bag. Pay for the entire purchase and retain a receipt to submit for reimbursement.
    i. Record on the receipt the ID of the area and shop, number of packs purchased, total price of the purchase, and date.
    ii. If the retailer does not provide receipts use a page and record the information listed above. Put the page along with the packs in a zip lock bag and write the area and the shop ID on the bag with a permanent marker.
f. Move to the next shop and ask for ST products other than purchased at previous shop.
g. Put stickers, write price, put in zip lock bag and put the IDs.
    i. If unique packs, other than previously purchased, are not avaible then move to the next shop.

As **two or more teams** will be collecting data simultaneously at **different locations**. Each team will purchase the available ST proucts. During **In-house assessment** of the products **duplication** will be removed.

For peer review only

Coding of each unique product will be done during In-House assessment.

In case if the shop is closed, no consent is given, donot sell STor donot sell ST to strangers, move to the next shop. Ask each shop for new products. If you find a new product, purchase and label it. Otherwise, don't buy duplicate product brands.

Equipment needed for field collection

Shoulder Bags (For carrying products)	Rubber bands
Zip Lock Bags	Stickers
Pens	Cash
Writing Pads	Umbrella

Data entry

An excel sheet will be given to the Data collectors. This sheet will contain information regarding the mapping of POS. It will be filled by Data collectors accordingly and will be submitted to the field supervisors on daily basis.

For peer review only

Appendix 2

Data fields for the digital ASTRA SLT product inventory form*

a) Study site________ (P1- P2)(B1-B2) (I1-I2)

b) Product Type_______(01-18)

c) Product unique ID_________________ (Study site/Product Type/product number)

d) Name of brand___________________
E.g. Bacha Naswar, JM Gutka …………..

e) Packaged _____________________ (Yes/No)

f) Hard/soft pack/sachet __________________ (coding 1-4, 4 for other)

g) Country of make________________ (coding 0 -195) (0 = Unknown)

h) Price (in local currency)__________________ (number)

i) Type of product _______________(coding 1-18, 18 for other)

j) Analyses status _____________ (Yes/No)

*Adapted from the TPackSS online product inventory/registration form, available at https://globaltobaccocontrol.org/tpackss/

For peer review only

Appendix 3

In-house assessment of SLT products tool (Adapted from TAPS guidelines)

•

**1. PACKAGING & LABELLING** (Yes =1, No = 0)	Cigarettes	Cigar/ Cigarillo	Beedi	Hukaah/shees	Naswar	Gutka	Paan/ Betel	Tobacco Gum	Mawah	Zarda	Gul	Snus	Nasal Snuff	Khaini	Others
a) Brand Name															
b) Name of the manufacturer only															
c) Address of the manufacturer only															
d) Name & Address of the manufacturer															
e) Contents listed (Yes/No)															
f) Weight per content is given (Yes/No)															
g) Country of Origin															
h) Statements of health claims															
i) Statements of harm e.g. harm to babies in the womb.															
j) Written health warnings (Yes/No)															
k) The language of health claims, Harm, Warnings (if No skip to k)															
I. Regional language															
II. National language															
III. English															
IV. Another foreign language															
l) Pictorial Health warning (Yes/No)(if No then skip to "m")															
m) The proportion of package does it cover															
I. 25%															

For peer review only

II. 50%															
III. 75%															
IV. 100%															
n) Provision of quit information on packaging (Yes/No)															
o) Use of culturally specific references (such as special images, symbols, or colours, etc.) (see your law or speak to local advocates for details)															
p) Presence of double packs															
q) Use of words that suggest flavour or reduced strength(Yes/no)															

**2. PRICE AND TAXATION** (Yes =1, No = 0)	Cigarettes	Cigar/ Cigarillo	Beedhi	Hukaah/sheesha	Naswar	Gutka	Paan/ Betel Quid [illegible]	Tobacco Gum	Mawah	Zarda	Gul	Snus	Nasal Snuff	Khaini	Others
In what form is the product sold?															
a) Loosely sold?															
b) Loose, Mixed with other products (custom made)															
c) Packaged or wrapped by the retailer															
d) Pre-packaged by manufacturer															
e) If pre-packed then in the form of															
I. Packet															
II. Tin															
III. Bottle															
IV. Can															
V. Sachet															
VI. Box															
VII. If other please specify															

For peer review only

VIII. With branding															
f) The minimum unit of sale															
I. By quantity															
II. By weight															
g) Price of Unit customer pays															
h) Price of Unit retailer pays															

For peer review only

Appendix 4
POS observation tool

ASTRA - POINT OF SALE OBSERVATION TOOL (Adapted from STARS)

1. Date of visit:__/__/____ Start Time: __:__ End Time: __:__

2. Coder Name/ID:________________________

3. Point of Sale ID:_____/ ________ (Study site code/serial number)

4. Store Type: (Choose one)

a) General/Departmental Store ☐
b) Petrol pump/Gas station store ☐
c) Beer or Liquor store ☐
d) Grocery store ☐
e) Exclusive Tobacco shop ☐
f) Tea Stall ☐
g) Mobile cart ☐
h) Stationary cart ☐
i) Other (specify e.g. Discount shop):____

5. Can you survey this store? [If not, then select an option below and STOP.]

a) Yes, I can ☐
b) No, store does not exist ☐
c) No, store is closed ☐
d) No, environment unsafe for me ☐
e) No, asked to leave before completing the survey ☐
f) Other (specify):____

SECTION 1: OBSERVATION (POS display for mobile or stationary cart should be considered exterior)

EXTERIOR

6.

Are there any point-of-sale displays of the tobacco products **OUTSIDE** the shop? (Yes = 1, No =0)	Cigarettes	Cigar/ Cigarillo	Beedi	Hukaah/sheesha	Naswar	Gutka	Paan/ Betel Quid [illegible]	Tobacco Gum	Mawah	Zarda	Gul	Snus	Nasal Snuff	Khaini	Others
a) Shelf edging															
b) Dummy packs															
c) Display packs															
d) Display stands															
e) Any other types of advertising?															

7. Is the point of sale vendor within _________meter (average distance) vicinity of any educational institution?

8. Does store display a health warning sign (OUTSIDE) against tobacco use? (If No skip to 9) ☐

Products	Written	Graphical

For peer review only

a) Smoking tobacco		
b) Smokeless Tobacco		

INTERIOR

9. Does the store have a pharmacy counter? (Yes = 1, No =0) ☐

10. Does store display a health warning sign (INSIDE) against tobacco use? (If No skip to 11) ☐

Products	Written	Graphical
a) Smoking tobacco		
b) Smokeless Tobacco		

11.

Are there any point-of-sale displays of the tobacco products **INSIDE** the shop? (Yes = 1, No =0)	Cigarettes	Cigar/ Cigarillo	Beedi	Hukaah/sheesha	Naswar	Gutka	Paan/ Betel Quid [illegible]	Tobacco Gum	Mawah	Zarda	Gul	Snus	Nasal Snuff	Khaini	Others
a) Shelf edging															
b) Dummy packs															
c) Display packs															
d) Display stands															
e) Any other types of advertising?															

12.

**PRODUCTS OBSERVATION** (Yes = 1, No = 0)	Cigarettes	Cigar/ Cigarillo	Beedi	Hukaah/sheesha	Naswar	Gutka	Paan/ Betel Quid [illegible]	Tobacco Gum	Mawah	Zarda	Gul	Snus	Nasal Snuff	Khaini	Others
a) Sold here?															
b) Visible															
c) Loosely sold here?															
d) Loose, mixed with other products (custom made)															
e) Product within 1 feet range of confectionaries, toys, candy, gum, slushy/soda machines, or ice cream?															
f) Products within 3 feet range of floor?															
g) Any price promotions/Credit?															
h) Cross-product promotion with tobacco? (Give any															

For peer review only

other related product free on purchase of the mentioned product)															

SALES TO/BY MINORS

(Yes =1, No = 0)	**Smoking tobacco**	**Smokeless tobacco**
a) Did you observe any sales assistants under the age of 18 years in the POS?		
b) If yes, what are their expected ages? Please list.		
c) Did you observe the vendor was selling any tobacco product to young people under the age of 18?		

For peer review only

Appendix 5

POS vendor interview tool

(The products in the columns and their unit of sales will be revised after the inventory study)

PRICES & Sale		Cigarettes	Cigar/ Cigarillo	Beedhi	Hukaah/sheesha	Naswar	Gutka	Paan/ Betel Quid with tobacco	Tobacco Gum	Mawah	Zarda	Gul	Snus	Nasal Snuff	Khaini	Others
a) Unit price that customer pay (in local currency)																
b) Unit price that retailer pay																
c) Sales Tax (in local currency)																
d) Total number of units sold per day	By Quantity															
	By Weight															
e)	f) How was the price obtained															
I. Cashier/ owner/ shop keeper																
II. Advertised price																

SUPPORT FOR VIABLE ALTERNATIVES

13.

**2.1**Why do you sell tobacco products? Tick all that apply. (Options should not be disclosed until the shopkeeper answer. Tick all that apply and then probe with the options remaining)	**Smoking tobacco products**	**Smokeless tobacco products**
a) Good profit margin		
b) Products are in demand		
c) Family business		
d) No other viable alternative		
e) Brings people into my shop to buy other things		
f) Other reasons (please state)		
**2.2**Would you ever consider stop selling SLT? (Yes = 1, N0 = 2)		
If yes, for which of the following reasons? Tick all that apply (if No skip to 16) (Options should not be disclosed until the shopkeeper answer. Tick all that apply and then probe with the options remaining)		
I. A reduction in profit margin		
II. A reduction in demand		
III. Stricter regulations and inspections		
IV. Limited supply		
V. A viable alternative		
VI. Awareness about its harms		
VII. Other reasons (please state)		

For peer review only

SUPPLIES

14.

Where do you get your supplies? (Tick all that apply)	Cigarettes	Cigar/ Cigarillo	Beedhi	Hukaah/sheesh	Naswar	Gutka	Paan/ Betel	Tobacco Gum	Zarda	Mawah	Gul	Snus	Nasal Snuff	Khaini	Others
a) Wholesalers (Cash and carry)															
b) Manufacturers															
c) Importers															
d) Unknown source															
e) Another shop/ store															
f) Others (Please state)															

15.

Why do you buy this product from a particular supplier? (Tick all that apply)	Cigarettes	Cigar/ Cigarillo	Beedhi	Hukaah/sheesha	Naswar	Gutka	Paan/ Betel Quid	Tobacco Gum	Mawah	Zarda	Gul	Snus	Nasal Snuff	Khaini	Others
a) They are the only supplier															
b) They can supply the required amount															
c) They are cheap															
d) They are nearby															
e) They are a friend or family member															
f) They sell products or brands my customers want															
g) They give me incentives															
h) Another reason (please state)															

16.

What is the nature of the incentive by a particular supplier? (Tick all that apply)	Cigarettes	Cigar/ Cigarillo	Beedhi	Hukaah/sheesha	Naswar	Gutka	Paan/ Betel Quid	Tobacco Gum	Mawah	Zarda	Gul	Snus	Nasal Snuff	Khaini	Others

For peer review only

I. Discount															
II. Promotional Goods															
III. Point-of-sale displays															
IV. Free sample															
V. Others (please state)															

17.

Where are your suppliers based (Tick all that apply)	Cigarettes	Cigar/ Cigarillo	Beedhi	Hukaah/sheesha	Naswar	Gutka	Paan/ Betel Quid	Tobacco Gum	Mawah	Zarda	Gul	Snus	Nasal Snuff	Khaini	Others
a) Local															
b) Abroad															
c) If Abroad, Where?															
d) Do you have contact details of the supplier?															
e) If "d" Yes, Can you Provide the contact details															
f) If "e" Yes, Contact Details of suppliers	**(Type of Product)**														
	**(Type of Product)**														
	**(Type of Product)**														
	**(Type of Product)**														

For peer review only

TOBACCO ADVERTISING, PROMOTION, AND SPONSORSHIP

18. (Yes =1, No = 0)

How do you (retailer) try to retain your tobacco customers? Tick all that apply.	**Smoking tobacco**	**Smokeless tobacco**
a) Offer discounts		
b) Offer promotional goods		

c) Offer credit lines		
d) Good customer relationships		
e) Other ways (please state)		

SALES TO/BY MINORS

19.

(Yes =1, No = 0)	**Smoking tobacco**	**Smokeless tobacco**
d) Do you have any sales assistants under the age of 18 years?		
e) If yes, what are their ages? Please list.		
f) Are they allowed to sell tobacco products?		
g) Do you ever sell any tobacco products to young people under the age of 18?		
h) If no, why not?		

20.

**PROFIT**	Amount in local currency
a) Roughly what amount of profit do you make from daily sales (Overall)	
b) What amount of profit comes from daily cigarettes sale	
c) What amount of profit comes from daily smokeless tobacco sale	

EDUCATION AND PUBLIC AWARENESS

21. (Yes =1, No = 0)

**10.1** Are you aware of any laws that impact your business of selling tobacco products?	**Smoking tobacco**	**Smokeless tobacco**
a) If yes, please state which law(s) and how it affects your business		
**10.2** Are you visited by any inspector or regulator to inspect and/or test your tobacco products?	**Smoking tobacco**	**Smokeless tobacco**
a) Inspect products		
b) Test products		
c) Check your license or registration		
d) How many visits did you have in the last 3 years?		
e) What happens after the inspection?		
I. If found not in compliance, nothing happens		
II. If found not in compliance, there is a penalty		
III. If found in compliance, nothing happens		
IV. If found in compliance, you are given a certificate		
V. Any other consequences?		
**10.3** Do you have to disclose the contents of smokeless tobacco products to any statutory body?		

For peer review only

**10.4** Do you have to send the products to a laboratory, for testing after Inspection?		

Signature of the interviewer_________________________

For peer review only

BMJ Open

Compliance of smokeless tobacco supply chain actors and products with Tobacco Control laws in Bangladesh, India and Pakistan: Protocol for a multi-centre sequential mixed-methods study.

Journal:	*BMJ Open*
Manuscript ID	bmjopen-2019-036468.R1
Article Type:	Protocol
Date Submitted by the Author:	03-Apr-2020

Complete List of Authors:	khan, Zohaib; Khyber Medical University<br>Huque, Rumana; University of Dhaka, Economics<br>Sheikh, Aziz; University of Edinburgh, Division of Community Health Sciences<br>Readshaw, Anne; University of York Department of Health Sciences<br>Eckhardt, Jappe; University of York<br>Jackson, Cath; University of York, York Trials Unit<br>Kanaan, Mona; University of York, Health Sciences<br>Iqbal, Romaina; Aga Khan University, Department of Community Health Sciences and Medicine<br>Akhter, Zohaib; Aga Khan University<br>Garg, Suneela; Maulana Azad Med Coll, Community medicine<br>Singh, Mongjam; Maulana Azad Medical College<br>Ahmad, Fayaz; Khyber Medical University<br>Abdullah, S M; University of Dhaka Department of Economics<br>javaid, arshad; Khyber Medical University<br>A.Khan, Javaid; Aga Khan University, Department of Pulmonary medicine<br>Han, Lu; University of York Department of Health Sciences<br>Rahman, Aziz; Federation University Australia Faculty of Health<br>Siddiqi, Kamran; University of York, UK, Institute of Health Sciences

**Primary Subject Heading**:	Global health
Secondary Subject Heading:	Global health, Health policy
Keywords:	PUBLIC HEALTH, QUALITATIVE RESEARCH, STATISTICS & RESEARCH METHODS

SCHOLARONE™
Manuscripts

I, the Submitting Author has the right to grant and does grant on behalf of all authors of the Work (as defined in the below author licence), an exclusive licence and/or a non-exclusive licence for contributions from authors who are: i) UK Crown employees; ii) where BMJ has agreed a CC-BY licence shall apply, and/or iii) in accordance with the terms applicable for US Federal Government officers or employees acting as part of their official duties; on a worldwide, perpetual, irrevocable, royalty-free basis to BMJ Publishing Group Ltd ("BMJ") its licensees and where the relevant Journal is co-owned by BMJ to the co-owners of the Journal, to publish the Work in this journal and any other BMJ products and to exploit all rights, as set out in our licence.

The Submitting Author accepts and understands that any supply made under these terms is made by BMJ to the Submitting Author unless you are acting as an employee on behalf of your employer or a postgraduate student of an affiliated institution which is paying any applicable article publishing charge ("APC") for Open Access articles. Where the Submitting Author wishes to make the Work available on an Open Access basis (and intends to pay the relevant APC), the terms of reuse of such Open Access shall be governed by a Creative Commons licence – details of these licences and which Creative Commons licence will apply to this Work are set out in our licence referred to above.

Other than as permitted in any relevant BMJ Author's Self Archiving Policies, I confirm this Work has not been accepted for publication elsewhere, is not being considered for publication elsewhere and does not duplicate material already published. I confirm all authors consent to publication of this Work and authorise the granting of this licence.

For peer review only

Compliance of smokeless tobacco supply chain actors and products with Tobacco Control laws in Bangladesh, India and Pakistan: Protocol for a multi-centre sequential mixed-methods study.

Khan Z[1], Huque R[2,3], Sheikh A[4], Readshaw A[5], Eckhardt J[6], Jackson C [5,7], Kanaan M[5], Iqbal R[8], Akhter Z[8], Garg S[9], Singh MM[9], Ahmad F[1], SM Abdullah SM[2,3], Javaid A[1], Khan J[8], Han L[4], Rahman MA[10], Siddiqi K[5].

Authors' affiliations:

[1]Khyber Medical University, Pakistan; [2]ARK Foundation, Bangladesh; [3]Department of Economics, University of Dhaka, Bangladesh; [4]Usher Institute, University of Edinburgh, UK; [5]Department of Health Sciences, University of York, UK; [6]Department of Politics, University of York, UK; [7]Valid Research Ltd, Wetherby UK, [8]Aga Khan Medical University, Pakistan; [9]Maulana Azad Medical College, New Delhi, India; [10]Federation University, Australia.

Corresponding author: Dr. Anne Readshaw

Email: anne.readshaw@york.ac.uk

Abstract

Introduction

South Asia is home to more than 300 million smokeless tobacco (ST) users. Bangladesh, India, and Pakistan as signatories to the Framework Convention for Tobacco Control (FCTC) have developed policies aimed at curbing the use of tobacco. The objective of this study is to assess the compliance of ST point-of-sale (POS) vendors and the supply chain with the articles of the FCTC and specifically with national tobacco control laws. We also aim to assess disparities in compliance with tobacco control laws between ST and smoked tobacco products.

Methods and Analysis

The study will be carried out at two sites each in Bangladesh, India, and Pakistan. We will conduct a sequential mixed-method study with five components: i. Mapping of ST POS, ii. Analyses of ST samples packaging, iii. Observation, and iv. Survey interviews of POS, and v. In-depth interviews with wholesale dealers/suppliers/manufacturers of ST. We aim to conduct 900 POS survey interviews and observations, and 3-5 in-depth interviews at each study site in the three countries. Data collection will be done by trained data collectors. The main statistical analysis will report the frequencies and proportions of shops that comply with the FCTC and local tobacco control policies, and provide a 95% confidence interval of these estimates. The qualitative in-depth interview data will be analysed using the framework approach. The findings will be connected, each component informing the focus and/or design of the next component.

Ethics and dissemination

Ethical approvals for the study have been received from the Health Sciences Research Governance Committee at the University of York, UK. In-country approvals were taken from

For peer review only

the National Bioethics Committee in Pakistan, the Bangladesh Medical Research Council, and the Indian Medical Research Council. Our results will be disseminated via scientific conferences, peer-reviewed research publications, and press releases.

Keywords: Smokeless tobacco, South Asia, point of sale, tobacco control, Bangladesh, India, Pakistan

Word Count: 3999

Strengths and limitations

- This will be the first multi-country study on smokeless tobacco supply chain in South Asia that aims to map smokeless tobacco points of sale using GIS.
- Building upon the methods used by the global TPACKSS project, our proposed study will develop/update the inventory of smokeless tobacco products available in Bangladesh, India, and Pakistan.
- Purposive sampling of our main study sites may decrease the external validity of our methods and results.
- Owing to a fear of the relevant authorities, the answers to our survey questions on taxes and finances might not be completely accurate.
- We will not be able to do a regression analysis including shop characteristics for the pack analysis component, as we are only collecting unique packs and not all packs from each shop

Introduction

Smokeless tobacco (ST) refers to all tobacco products that are used without burning the product [1]. The International Agency for Research on Cancer estimates that ST contains more than 30 carcinogens [2]. The use of ST is associated with an increased incidence of head, neck and gastrointestinal cancers [3-5]. Additionally, ST use is associated with Oral Potentially Malignant Disorders (OPMDs) [6] and a variety of other oral and systemic diseases [7, 8]. An estimated 300 million people around the globe use ST products in some form, and the majority (≥85%) of these ST users live in South Asia [1, 9]. The use of ST products is particularly common in India, Bangladesh, and Pakistan, where it is considered an acceptable social norm [10]. This situation is further complicated by the presence of high amounts of Tobacco Specific Nitrosamines, the main cancer-causing agents in tobacco, in the products available in South Asia, compared to the rest of the world [11].

The Framework Convention for Tobacco Control (FCTC) is a global treaty that aims to enhance tobacco control measures and incorporate the provisions of the framework into their national tobacco control laws [12]. Although the FCTC is aimed at curbing all types of tobacco use, the focus of tobacco control in most countries has been on controlling smoking, with control of the non-smoking forms of tobacco often put on a back burner [13, 14]. The 2016 Global Progress Report on the implementation of FCTC highlighted gaps in the formulation and implementation of ST control policies in most parties to the FCTC [15]. Bangladesh, India, and Pakistan are signatories to the FCTC and have made variable progress in implementing the articles of the framework by developing national tobacco control policies [16].

The tightening up of smoking regulations has been associated with a reported shift from smoking to ST use in some South Asian countries [9, 17]. The increase in ST use can be

attributed to a variety of factors, including, but not limited to cultural acceptance, perceived safety of ST products compared to smoking, harm-reduction, low prices, and lax or absent ST control laws [14].

A 2018 report from the Global Knowledge Hub on Smokeless Tobacco indicated progress being made with regards to ST control policies and regulations in South Asia [14]. Many of these regulations and laws are aimed at demand reduction via various interventions at the tobacco points of sale e.g. removal of tobacco products displays and restrictions and bans on advertisement of tobacco products at the point of sale [18, 19]. The success of these regulations and laws in curbing the tobacco epidemic in South Asia largely depends on the degree of compliance with the implementation of these laws and regulations [20]. However, the evidence on the implementation of the ST control laws and regulations at the point of sale (POS) in the context of India, Bangladesh, and Pakistan is scarce. Most of the previous work carried out in these countries focused on tobacco advertisement and promotion at the POS and health warnings on ST packaging [21-26], with little emphasis on the other provisions of the FCTC, such as sale to/by minors, price and taxation, and provision of viable alternatives. As such, there is a need for a comprehensive assessment of the compliance of POS in India, Pakistan, and Bangladesh with the local ST control policies and the specific articles of the FCTC. `

This multi-country sequential mixed methods study aims to provide a comprehensive assessment of compliance of ST POS and products with the tobacco control laws in our three target countries. Furthermore, we aim to elicit information on any disparities between the implementation of FCTC/local laws for ST and smoking. We will also develop a country-specific inventory of tobacco products, to gain insight into the diversity of the products available in the local market, as well as identifying illicit products. Additionally, we will assess the barriers and facilitators to compliance of the ST wholesale dealers and suppliers with the different provisions of the FCTC/local laws.

Methods and Analysis

Study design

We will conduct a sequential mixed-methods study, incorporating quantitative work (geo-mapping of ST POS, in-house analyses of ST products, POS survey interviews and observations and qualitative (in-depth-interviews with supply chain actors e.g. wholesale dealers, suppliers) work. The findings from each component will be connected and used to inform the focus and/or the design of the next component i.e. Mapping of the POS will allow us to build a sampling frame from which we will randomly choose survey interview respondents, the ST pack analyses will provide us an insight into manufacturing and sale practices that can be incorporated into the POS survey interview tool and the topic guide for in-depth interviews. The findings from the survey interviews and observations of the POS will guide the development of country specific topic guides for the In-depth Interviews.

Study sites

We will conduct this study in Bangladesh, India and Pakistan. In each country, we have purposively selected two sites based on the use and diversity of the available ST products. In India, the study will be conducted in the North-East and North-West districts of Delhi. In Bangladesh, the study will be conducted in the districts of Dhaka and Rangpur. In Pakistan, the study will be conducted in the districts of Karachi and Peshawar. In Pakistan and Bangladesh, the selected sites are the hubs of ST use and manufacture, for India, our rationale for choosing

For peer review only - http://bmjopen.bmj.com/site/about/guidelines.xhtml

the sites was based on Delhi being a metropolitan city with diverse population and products and also the logistics available to study team.

Study duration

The data collection will be carried out from June 1, 2019, to May 30, 2020 and analyses will be completed by March 30, 2021.

General sampling strategy

We will use a multi-stage mixed sampling technique for the study (Fig. 1).

Stage 1: Within each of the selected administrative areas (District/Division), we will purposively select one predominantly urban and one predominantly rural sub-district. Our study sites included districts/areas, which comprise urban, peri-urban, and rural areas. We will use the available local government documents, latest census and expert opinion of local researchers to identify two sub-districts/area at each site, one predominantly urban and the other predominantly rural/peri-urban.

Stage 2: We will randomly select three smaller administrative units per sub-district. These will be our Primary Sampling Units (PSU).

Stage 3: From each PSU, we will randomly select two enumeration blocks/neighbourhood areas/villages; these will be our Secondary Sampling Units (SSU).

Data collection (General considerations)

At each study site, we will deploy a team of trained data collectors to administer the quantitative data collection instruments (comprising the geo-mapping, in-house analysis of the ST products packaging, structured POS vendor survey interview and observation components). Data collectors will be trained on how to administer the various study tools beforehand. They will also receive training in obtaining informed and written/verbal consent. Calibration of the product in-house analysis component will be done via video conferencing involving study teams from all sites. A qualitative research specialist will train one person at each participating site to conduct and transcribe the in-depth interviews with the suppliers and/or manufacturers. Data will be monitored for quality and completeness by the site leads/study leads/principal investigator. We will also conduct small pilot studies in each country to field-test our study tools.

Data management

Data will be initially collected on a paper-based questionnaire and then transferred to a central database kept on a secure server at the University of York. Each ST product, POS vendor, supplier, and manufacturer recruited in the study will be allotted a unique identification number and the recorded data will not contain any identifiable information about them. Only the study leads/principal investigator/study statistician will have access to the database and data collection tools. All filled questionnaires will be stored in locked filing cabinets at the collaborating institutions in each of the study centres.

In line with the General Data Protection Regulations (GDPR) of the European Union (EU) and the Research Governance Framework for Health and Social Care Research, data will be securely archived by the University of York for a minimum of five years after the completion of the study. Following authorisation from the sponsor, arrangements for confidential

For peer review only

destruction will then be made. Recordings from the in-depth interviews will be deleted at the end of the study. If an individual withdraws consent for their data to be used, it will be destroyed without breach of confidentiality.

Study component-specific methods

POS Geo-Mapping

Eligibility criteria for POS

A POS will be eligible for inclusion in the study if it sells any form of ST. The common types of tobacco POS that might be eligible for our study are:

a. General/departmental stores
b. Petrol pump/gas station stores
c. Beer or liquor stores
d. *Paan* (betel-quid) shops
e. Grocery stores
f. Exclusive tobacco shops
g. Discount shops
h. Mobile vendors/carts
i. Stationary carts
j. Country-specific additional POS types.

Identifying and mapping eligible POS

To identify the eligible POS, we will employ standard approaches for tobacco POS audits [27]. This will entail:

i. Developing a list of tobacco sellers in the selected areas by collecting the details of local vendors who have a license to sell tobacco products (if applicable), and/or the acquisition of general lists of local businesses/sellers from trade unions and/or local administrative authorities and combing potential tobacco POS from these lists.
ii. This will be followed by "ground-truthing" by trained data collectors, who will visit these shops to ascertain, a. The existence of the POS, and b. The sale of ST products at these POS.
iii. Once ascertained, each eligible POS will be allotted a unique identification number and its location will be recorded via the Google Maps® digital application. The application gives its users their current location on Google Maps, the Global Positioning System (GPS) coordinates, and the geolocation status.
iv. If no prior lists (license holders or businesses/sellers) are available at the study location(s), our data collection team will do "canvassing" of the ST POS by walking along all the primary and secondary roads in that area and identifying eligible ST POS (Appendix 1).
v. Additionally, we will request each ascertained POS vendor to identify other ST POS, which will be added to the list.
vi. If data collectors observe a mobile vendor during the ground-truthing or canvassing at the study sites, the area where the team establishes the first contact with that vendor will be marked as his/her location.

For peer review only

Measuring the density of ST POS

We will collect the most recent official population census data of our selected study sites from the relevant department(s) in each country. If such data are not available, we will use unofficial estimates gathered from the local administration. The density of the POS will be calculated per 1,000 population at our study sites.

Measuring the distance of the POS from schools

We will acquire the list of all schools along with their GPS coordinates, situated in the selected SSUs from the local education departments. If the data is not available, our team will visit each school physically, drop a pin at the school's gate using Google Maps and record the coordinates. We will plot all the ST POS and the schools on a digital map and to assess if there is compliance of the local laws that ban the sale of tobacco products in the vicinity (distance will vary according to country) of the schools.

ST products in-house analysis

Eligibility criteria

All brands of ST products available in the markets at the study sites will be procured. In order for a product to be eligible, it must contain tobacco. The collection of the ST products will be based on the brand names and not the generic product e.g. all brands of *Gutkha* available in the local market will be collected. Loose products that are not sold in packaging will be eligible, but only one generic sample of the product will be procured. The most commonly sold brand/make of cigarettes, cigars, cigarillos, and beedi according to the selected vendor, will be procured.

Sample collection and storage

All ST sale shops/vendors identified in the selected SSUs by the geo-mapping exercise will be approached to procure the ST products that are available in the local markets. We will employ an adapted form of the unique packs sampling technique used for the TPackSS samples collection [28]. One of every unique pack of ST (any pack with at least one difference in an exterior feature of the pack including size, brand name, presentation, colours, cellophane, packaging material (i.e. hard, soft, tin), and the inclusion of a promotional item) available in the store will be purchased. This sampling will begin at a randomly-selected eligible vendor. After the purchase at the initial vendor, at each of the remaining eligible vendors, one of every ST pack that was not already purchased at a previous outlet will be purchased. If the selected vendor does not have any new packs (packs that were not purchased at another outlet), the sample collection team will move on to the next eligible vendor. Sample collection will continue until the team has approached the last eligible vendor. The sample collection team will ascertain the retail price of each procured pack from the vendor. They will also ascertain if the prices are inclusive of tax or not. The sample collection team will neither request nor accept a reduced price, in case the vendor offers a discount due to the large quantity being purchased, other than in those cases where discounted products are already being offered to all customers as a part of a promotion. All such general promotions, and individual "reduced price" offers will be recorded on a field notes sheet provided to each data collector.

Product analyses

All the procured samples will be sorted by product type, and each sample will be allotted a unique identification number marked on the Ziploc bag with indelible ink. The study team at each participating institution will analyze one sample at a time, carefully locking it back in the bag before moving onto the next sample. A physical inventory of all the products will be

For peer review only

developed at each site and the data related to the products e.g. unique identification number, product family, purchase price and date of purchase will be fed into a digital inventory form (Appendix 2) adapted from the TPackSS inventory data entry form.

The in-house assessment of the ST products will involve assessment and recording of their size, price, tax disclosure, ingredient lists, health warnings, manufacturer details, etc. Our study tool (Appendix 3) has been adapted from the TAPS standard tool. We will collect the samples by brand names and assess whether disparities exist between ST product categories and/or different manufacturers. We will also collect data on the country of manufacture and analyze if differences in compliance to the relevant laws can be explained by differences in the country of manufacture and/or "smuggled/illicit" designation of the product. The definition of illicit products for each participating site will be developed after consulting local stakeholders. We will also analyze the collected cigarette packs, to assess their compliance with the local laws/FCTC. This will also help us in in the identification of any disparity in the local laws for ST and those for cigarettes.

POS Survey Interviews and Observation

Sample size

For the POS observation and Interviews, we calculated sample sizes for the individual countries based on the prevalence of non-compliance with specific provisions of the local tobacco control laws in the three countries. The point estimates were derived from published literature i.e. 20% of shops selling tobacco to minors in Pakistan [29], 19.4% POS non-compliant with COTPA regulations regarding sizing of the advertisements in India [30], and 20% POS non-compliant with tobacco advertisement and promotion laws in Bangladesh [31].

Using the formula sample $\boldsymbol{n} = \mathbf{[DEFF*Np(1\text{-}p)]/\ [(d^2/Z^2_{1-\alpha/2}*(N\text{-}1)+p*(1\text{-}p)]}$, and assuming a 20% prevalence of non-compliance, a confidence interval of 95%, and an absolute precision of 7%, the sample size for each country came out to be 251. After accounting for a 15% non-response (based on our ST supply chain study in Pakistan and Bangladesh) [32], the sample size was rounded up to 290 per country. All sample size calculations were carried out in Open Epi software [33].

Sampling strategy

We will follow the general sampling strategy (Fig 1). Once a sampling frame of eligible ST POS in each SSU has been developed through the geo-mapping, a random sample of the 13 POS vendors will be recruited from each SSU.

Recruitment

After providing brief information about the study to the person-in-charge of the POS at the time of the store observation, we will formally ask them to provide written consent to participate in both the POS observation and POS vendor interview components of the study. Those who do not agree to participate after receiving the study information will not be enrolled in the study. The reasons for declining to participate in the study will be recorded. Following consent, the data collector will proceed to data collection. The consent provider, who could be either the POS owner (not land-lords), POS manager, or sales assistant(s) will be eligible for interview.

Data collection and tools

An adapted version (Appendix 4) of the Standardized Tobacco Assessment for Retail Settings (STARS) tool [34], will be used to record the observations about product promotion, advertisement, and health warnings, etc. We will observe both the exterior and interior of the

For peer review only

shops to assess the compliance with FCTC/local laws. The tool consists of data fields on health warnings inside and outside the shop, product advertisement, promotions, display shelves, dummy packs, product placement, products sold as confectioneries, and cross-promotion. We will also collect similar data for cigarettes and other smoking products, for comparison.

Our interview tool (Appendix 5) has been adapted from a survey instrument that has already been field-tested in the UK, Bangladesh, Nepal, and Pakistan [32]. The tool will be translated into local languages at each study site. In addition to a series of close-ended questions on ST products regarding their supply, sale, popularity, prices, profits, and taxes, the tool also includes similar questions for cigarettes, to allow for comparative analysis. We will collect data on mediating/moderating variables that could act as barriers/facilitators to the increased implementation of the FCTC/local laws. These variables are awareness of local tobacco control legislation, standards, and obligations, awareness of adverse health effects of ST, the effect of health warnings on sales, market share of SLT products, overall sales related to availability of tobacco products for non-exclusive vendors, incentives by manufacturers/suppliers, profit margins, regular inspections, penalties for non-compliance, and availability of viable alternatives.

Data analysis

The main statistical analysis will report the frequencies and proportions of shops that comply with the WHO FCTC and local tobacco control policies, as measured by the different items on the survey questionnaire and provide a 95% confidence interval of this estimate. We will also provide summaries for the other categorical variables using frequencies and percentages and for continuous variables, we will provide means and standard deviations in addition to graphical representation. Medians and inter-quartile ranges will also be computed if the data are skewed.

We will use logistic regression to explore the bivariate association of compliance with shop characteristics, such as the type of shop, adjusting for the study design. We will further expand these regression models to explore the association of compliance with multiple explanatory variables adjusting for the study design. Where possible, figures related to ST sales will be compared with those of cigarette sales, using similar approaches to the above. We will consider a p-value of less than 0.05 to be statistically significant. A detailed statistical analysis plan will be developed prior to the main analysis, which will include any *a priori* subgroup analyses and strategies for handling missing data. We will use STATA 15 for analysis.

In-depth Interviews (Supply chain)

Participants, sampling and recruitment

At each of the six study sites, we will identify, and collect contact details of, at least five supply chain actors (from manufacturers, wholesalers, suppliers) with the help of the POS survey respondents. From this list we will purposively recruit 03-05 participants at each site ensuring that the sample includes at least one manufacturer and one supplier from each site.

Data collection

A topic guide will be developed for the interviews. The contents of the guide will be based on the findings from the POS survey. That is, the responses in the survey that warrant further exploration will be identified by the study teams in each country and the topic guide aimed at eliciting further information on the responses will be developed by the qualitative research team. We will start with a standard topic guide and then tailor it to the findings from the different countries

For peer review only

The interviews will be conducted at locations that are easily accessible to participants, and where they are confident to speak openly, such as their workplaces or the offices of ward/union council office bearers or the village chief's house/hujra. Each interview will last 30-60 minutes. A trained researcher will conduct the interviews in the local language and will be recorded digitally.

Data analysis

The interviews will be transcribed verbatim, translated into English and analysed using the Framework approach [35]. The framework approach is not associated with a particular epistemological viewpoint or theoretical approach and, as such, is not necessarily concerned with generating social theory. The approach can be used in inductive or deductive analysis or a combination of the two [36]. We will be using an inductive approach and develop a framework matrix, informed by the study objectives, the interview topics and data from preliminary interviews. The data from the three countries will be charted into the same matrix; and then reviewed and interrogated to compare and contrast views, seek patterns, connections and explanations within the data, including comparison across the three countries. Excel software will aid data handling.

Patient and public involvement

The study is supported by stakeholder groups in each participating country. The stakeholder groups consist of health professionals, policy-makers, representatives from the general society, students and influencers. The stakeholder groups meet once every year, and so far have been involved in the design of the study tools, refinement of research questions and data collection methods. These group will also be involved in the dissemination of findings form the study.

Ethics and Dissemination

We will adhere to the fundamental principles of human rights as per the Helsinki Declaration. Ethical approval has been obtained from the University of York "Health Sciences Research Governance Committee". Additionally, each participating South Asian institution in ASTRA has obtained ethical approvals from their institutional/national ethics bodies. All potential participants will be provided with information regarding the project and written/verbal consent will be taken from each participant prior to recruitment. Consent forms will contain personal details including name, date of birth, and address.

All information collected during the course of the study will be kept strictly confidential. There will be restricted access and disposal arrangements for the participants' personal details. Information will be held securely on paper/electronically at the University of York. Data (other than consent forms) transferred to or from the University of York will be coded with a study enrolment number and will not have participants' identifiers. We have studied the conditions under which disclosures to law enforcement agencies are required and are satisfied that the chances of these occurring (criminal activities) are highly unlikely.

To engage our academic audience and the research fraternity, we will publish the results of the study in peer-reviewed journals. We will also present our findings at an international public health conference and an international tobacco control conference. We will also publish an abstract of our findings on the websites of all the partner institutions. We will also present our work to regional research meetings. Additionally, we will incorporate the learning from this project into academic lectures for Master students of public health. We have already established national stakeholders groups in all three target countries, who will guide the study conduct and help us in the dissemination of our findings.

For peer review only

Declarations

The authors declare no conflict of interest.

Funding

This research is funded by the UK's National Institute for Health Research (NIHR) [ASTRA (Grant Reference Number 17/63/76)]. The views expressed in this publication are those of the authors and not necessarily of the NIHR or the Department of Health and Social Care.

Authors contribution

ZK, KS, RH, SG, MMS conceived the study, wrote the first and all subsequent drafts.

AR, FA, AMR, ZA, ASM, RI, AJ, JK and AS wrote the initial drafts and commented on the final draft.

MK and LH commented on all drafts and wrote up the sampling and analyses section.

JE and CJ commented on initial and final versions of the draft and wrote up the section on mixed methods and in-depth interviews.

Figure Legend

Fig 1. General sampling strategy to be used at all sites.

a. Within each of the selected administrative areas (District/Division), we will purposively select one predominantly urban and one predominantly rural sub-district. We will use the available local government documents, latest census and expert opinion of local researchers to identify two sub-districts/area at each site, one predominantly urban and the other predominantly rural/peri-urban.
b. We will randomly select three smaller administrative units per sub-district. These will be our Primary Sampling Units (PSU).
c. From each PSU, we will randomly select two enumeration blocks/neighbourhood areas/villages; these will be our Secondary Sampling Units (SSU).
d. From each SSU we will recruit up to 13 POS.
e. Required sample size/country is 290, through this strategy we will be able to recruit up to 312 POS.

References

1. Eriksen M, Mackay J, Ross H. The tobacco atlas. American Cancer Society; 2013.

2. IARC Working Group on the Evaluation of Carcinogenic Risks to Humans, World Health Organization, International Agency for Research on Cancer. Smokeless tobacco and some tobacco-specific N-nitrosamines: World Health Organization; 2007.

3. Guha N, Warnakulasuriya S, Vlaanderen J, Straif K. Betel quid chewing and the risk of oral and oropharyngeal cancers: A meta-analysis with implications for cancer control. International Journal of Cancer. 2014;135(6):1433-1443.

4. Boffetta P, Hecht S, Gray N, Gupta P, Straif K. Smokeless tobacco and cancer. Lancet Oncol. 2008;9(7):667-675.

5. Khan Z, Dreger S, Shah SMH, Pohlabeln H, Khan S, Ullah Z, Rehman B, Zeeb H. Oral cancer via the bargain bin: The risk of oral cancer associated with a smokeless tobacco product (Naswar). PloS one. 2017;12(7):e0180445.

6. Khan Z, Khan S, Christianson L, Rehman S, Ekwunife O, Samkange-Zeeb F. Smokeless tobacco and oral potentially malignant disorders in South Asia: a systematic review and meta-analysis. Nicotine and Tobacco Research. 2017;20(1):12-21.

7. Siddiqi K, Shah S, Abbas SM, Vidyasagaran A, Jawad M, Dogar O, Sheikh A. Global burden of disease due to smokeless tobacco consumption in adults: analysis of data from 113 countries. BMC Med. 2015;13:194-015-0424-2.

8. Sinha DN, Abdulkader RS, Gupta PC. Smokeless tobacco-associated cancers: A systematic review and meta-analysis of Indian studies. International Journal of Cancer. 2016;138(6):1368-1379.

9. Suliankatchi RA, Sinha DN, Rath R, Aryal KK, Zaman MM, Gupta PC, Karki KB, Venugopal D. Smokeless Tobacco Use is "Replacing" the Smoking Epidemic in the South-East Asia Region. Nicotine Tobacco Res. 2017;1:6.

10. Gupta PC, Ray CS. Smokeless tobacco and health in India and South Asia. Respirology. 2003;8(4):419-431.

11. Stanfill SB, Connolly GN, Zhang L, Jia LT, Henningfield JE, Richter P, Lawler TS, Ayo-Yusuf OA, Ashley DL, Watson CH. Global surveillance of oral tobacco products: total nicotine, unionised nicotine and tobacco-specific N-nitrosamines. Tob Control. 2011;20(3):e2.

12. Shibuya K, Ciecierski C, Guindon E, Bettcher DW, Evans DB, Murray CJ, WHO Framework Convention on Tobacco Control. WHO Framework Convention on Tobacco Control: development of an evidence based global public health treaty. BMJ. 2003;327(7407):154-157.

13. WHO Study Group on Tobacco Product Regulation. Advisory note: Waterpipe tobacco smoking: health effects, research needs and recommended actions for regulators. 2015.

14. Zaatari GS MD, Bazzi A. Impact of the WHO FCTC on non-cigarette tobacco products. Tob Control. 2018.

15. Chung-Hall J, Craig L, Gravely S, Sansone N, Fong GT. Impact of the WHO FCTC over the first decade: a global evidence review prepared for the Impact Assessment Expert Group. Tob Control. 2018.

For peer review only

16. Shang C, Lee HM, Chaloupka FJ, Fong GT, Thompson M, O'Connor RJ. Association between tax structure and cigarette consumption: findings from the International Tobacco Control Policy Evaluation (ITC) Project. Tob Control. 2018.

17. Khan Z. A gaping gap (smokeless tobacco control in Pakistan). Tobacco induced diseases. 2016;14(1):36.

18. World Health Organization. Monitoring tobacco control among youth in countries of the South-East Asia Region: 2018. 2018.

19. World Health Organization. Second joint mission of the United Nations Interagency Task Force on the Prevention and Control of Noncommunicable Diseases, Sri Lanka, 23-27 April 2018. 2018.

20. He Y, Shang C, Huang J, Cheng KW, Chaloupka FJ. Global evidence on the effect of point-of-sale display bans on smoking prevalence. Tob Control. 2018.

21. Tobacco Pack Surveillance System (TPackSS). Health Warning Label Compliance: India‑2013. Baltimore, MD: Johns Hopkins Bloomberg School of Public Health. https://www.globaltobaccocontrol.org/tpackss/sites/default/files/tpackss_smokeless_HWL_10_22_2018.pdf

22. Iacobelli M, Saraf S, Welding K, Smith KC, Cohen JE. Manipulated: graphic health warnings on smokeless tobacco in rural India. Tobacco Control. 2020 Mar 1;29(2):241-2.

23. Mullapudi S, Britton J, Kulkarni MM, Moodie C, Kamath VG, Kamath A. A pilot study to assess compliance and impact of health warnings on tobacco products in the Udupi district of Karnataka State, India. Tobacco induced diseases. 2019;17.

24. Rahman SM, Alam MS, Zubair AB, Shahriar MH, Hossein M, Alam MS, Hefler M. Graphic health warnings on tobacco packets and containers: compliance status in Bangladesh. Tobacco control. 2019 May 1;28(3):261-7.

25. Cohen JE, Brown J, Washington C, Welding K, Ferguson J, Smith KC. Do cigarette health warning labels comply with requirements: A 14-country study. Preventive medicine. 2016 Dec 1;93:128-34.

26. Tobacco Pack Surveillance System (TPackSS). Health Warning Label Compliance: Bangladesh‑2016. Baltimore, MD: Johns Hopkins Bloomberg School of Public Health. http://globaltobaccocontrol.org/tpackss/sites/default/files/Tpackss_Bangladesh_healthwarningII_090517.pdf

27. Feld AL, Johnson TO, Byerly KW, Ribisl KM. How to Conduct Store Observations of Tobacco Marketing and Products. Prev Chronic Dis. 2016;13: E25.

28. Smith K, Washington C, Brown J, Vadnais A, Kroart L, Ferguson J, Cohen J. The Tobacco Pack Surveillance System: A Protocol for Assessing Health Warning Compliance, Design Features, and Appeals of Tobacco Packs Sold in Low- and Middle-Income Countries. JMIR Public Health Surveill. 2015;1(2):e8.

For peer review only

29. CTC Pakistan. Stubbing it out. In Edited by Anonymous Pakistan: Society for Alternative Media and Research; 2017:[Anonymous].

30. Patel S, Rendell H, Maudgal S, Oswal K. Tobacco industry tactics with advertisements at the point of sale in Mumbai. Indian J Cancer. 2013;50(3):245-249.

31. Bhuiyan MEH, Ovi FH, Alam MM, Farheen I. Documentation on Tobacco Advertising, Promotion & Sponsorship in Bangladesh. 2014;ISBN: 978-984-33-6739-6.

32. Siddiqi K, Scammell K, Huque R, Khan A, Baral S, Ali S, Watt I. Smokeless Tobacco Supply Chain in South Asia: A Comparative Analysis Using the WHO Framework Convention on Tobacco Control. Nicotine Tob Res. 2016;18(4):424-430.

33. Dean A, Sullivan K, Soe M. OpenEpi: Open source epidemiologic statistics for public health, version. 2014.

34. Henriksen L, Ribisl KM, Rogers T, Moreland-Russell S, Barker DM, Sarris Esquivel N, Loomis B, Crew E, Combs T. Standardized Tobacco Assessment for Retail Settings (STARS): dissemination and implementation research. Tob Control. 2016;25(Suppl 1):i67-i74.

35. Ritchie J, Lewis J. McNaugton Nicholls C, Ormston R. (2014). Qualitative Research Practice: A guide for social science students and researchers. London: SAGE.

36. Gale NK, Heath G, Cameron E, Rashid S, Redwood S. Using the framework method for the analysis of qualitative data in multi-disciplinary health research. BMC medical research methodology. 2013 Dec;13(1):117.

For peer review only

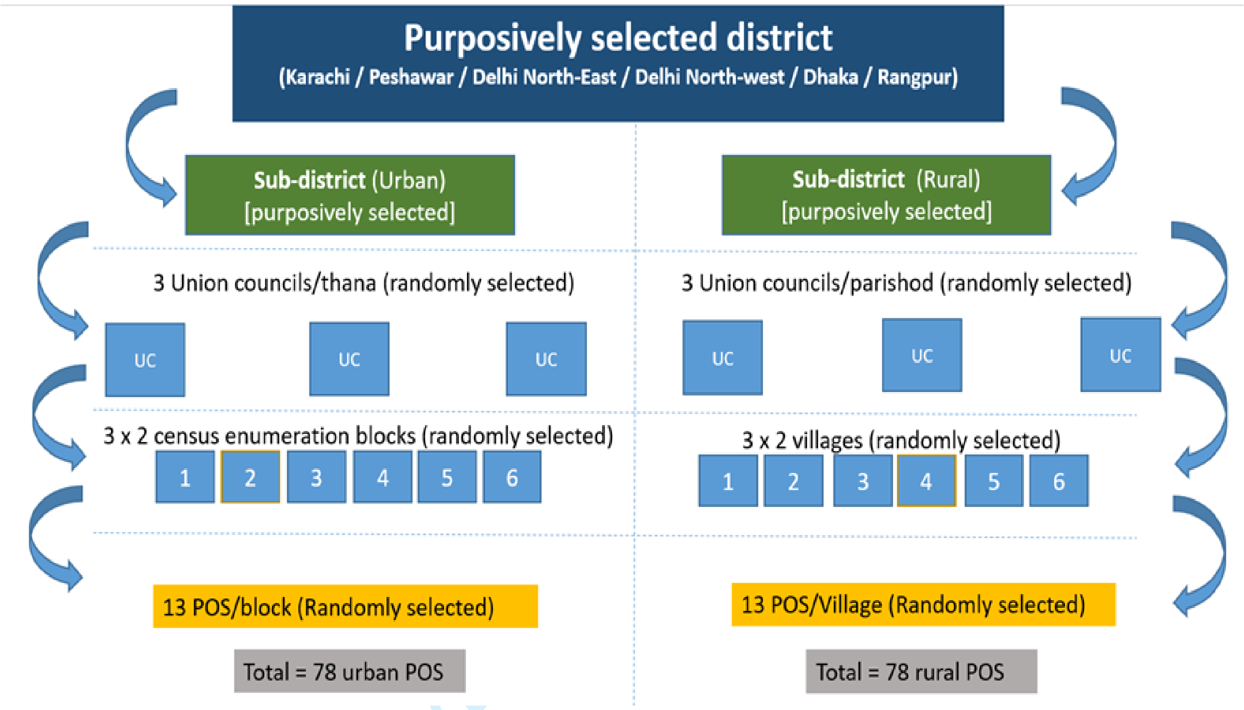

Fig 1. General sampling strategy to be used at all sites.

a. Within each of the selected administrative areas (District/Division), we will purposively select one predominantly urban and one predominantly rural sub-district. We will use the available local government documents, latest census and expert opinion of local researchers to identify two sub-districts/area at each site, one predominantly urban and the other predominantly rural/peri-urban.
b. We will randomly select three smaller administrative units per sub-district. These will be our Primary Sampling Units (PSU).
c. From each PSU, we will randomly select two enumeration blocks/neighbourhood areas/villages; these will be our Secondary Sampling Units (SSU).
d. From each SSU we will recruit up to 13 POS.
e. Required sample size/country is 290, through this strategy we will be able to recruit up to 312 POS.

For peer review only - http://bmjopen.bmj.com/site/about/guidelines.xhtml

Appendices

1. Field Protocol for Data Collection
2. Product Inventory Form
3. Product In-house assessment tool
4. Point of sale observation tool
5. Point of sale Interview tool

For peer review only

Appendix 1

ASTRA Sellers Study Field Protocol

After selection of villages and enumeration blocks in rural and urban areas respectively through ASTRA sellers study sampling strategy, data collectors will be sent to these villages and enumeration blocks for mapping of the POS and collection of samples for In-House assessment. Following the is the field protocol for Mapping and Sample collection.

Inclusion criteria for POS

A POS will be eligible for inclusion in the study if it sells any form of Smokeless Tobacco. The common types of tobacco POS that are eligible for our study include:

a. General/departmental stores
b. Petrol pump/gas station stores
c. Beer or liquor stores
d. *Paan* (betel-quid) shops
e. Grocery stores
f. Exclusive tobacco shops
g. Discount shops
h. Mobile vendors/carts
i. Stationary carts
j. Any Other ________________. (Country teams can add specific POS not in the list)

The data collectors will ascertain the eligibility of an establishment by first inquiring about the availability of ST at that establishment and then proceeding for procurement of ST samples for the "In-house analysis" component of the study. Once they have established the POS as eligible, they will drop a pin on the Google Map as per the method used for "Mapping of POS".

Walking Protocol

The data collector will observe the following guidelines to map the POS in villages and enumeration blocks.

A. ***When Map of the Roads is available***

1. Obtain the map of the specific area (Village/enumeration block) with all the primary roads marked on it.
2. Mark the geographic boundaries of that village/enumeration block on the map.
3. Number all the roads for identification and ease e.g. 1,2,….n
4. Identify the start point of each road as **"A"** and end point as **"B"**.
5. Start from point **A** of the road No.1, and map all the POS available on both sides of the road until you reach point **B** of the road.
6. Repeat the process until all the roads are covered.

For peer review only

7. While mapping the POS at each road ask the shopkeepers for POS away from the roads. Visit those POS and map them.

B. ***When Map of the Roads is not available***

1. Identify the geographic boundaries of the village or enumeration block on the map.
2. Identify and mark all the primary roads on the map with the help of key informants.
3. Number all the roads for identification and ease e.g. 1,2,….n
4. Identify the start point of each road as **"A"** and end point as **"B"**.
5. Start from point **A** of the road No.1, and map all the POS available on both sides of the road until you reach point **B** of the road.
6. Repeat the process until all the roads are covered.
7. While mapping the POS at each road ask the shopkeepers for POS away from the roads. Visit those POS and map them.

Notes:

- In case if the shop is closed, and you suspect that it as a ST POS, map it by standing at the door of the shop. Mention in the comments as "closed".
- In case of mobile vendor/carts, map at the location where you find them.

For peer review only

Mapping of POS

Since this component uses Images from **Google Maps**, it cannot be published. The authors are willing to share the detailed method upon request.

Purchase of Products

Data collectors will be sent in pairs to map the POS. Arriving at the first shop one data collector will purchase the products and another one will map the shop;

1. The purchaser will visit the counter and ask for all the available brands of following smokeless tobacco (ST) products.
    a) Naswar
    b) Paan/ Betel Quid
    c) Gutka
    d) Tobacco Gum
    e) Gul
    f) Snus
    g) Zarda
    h) Nasal Snuff
    i) Khaini
    j) Others__________(Country Specific)

Complete **unique packs sample** by purchasing all unique smokeless tobacco packs available.
Unique Pack Definition: any pack with at least one difference in an exterior feature of the pack including: size, brand name, presentation, colors, packaging material (i.e. hard, soft, tin), and inclusion of a promotional item.

a. Face the counter and request to purchase one of each unique ST pack.
b. Capture the price of each pack. Place a sticker on the back of the pack and record the price on the sticker.
    i. For retailers where the price is not given, do not request nor accept a reduced price if the vendor offers a discount due to the large quantity being purchased.
c. Ask the shopkeeper to confirm that one of each ST pack they sell has been acquired and purchase any additional packs identified.
d. Also acquire one of the most commonly sold cigarette pack. Put a price tag on the pack. If cigarette packs is not available with the shop, continue with the process.
e. Place the packs in a zip lock bag. Pay for the entire purchase and retain a receipt to submit for reimbursement.
    i. Record on the receipt the ID of the area and shop, number of packs purchased, total price of the purchase, and date.
    ii. If the retailer does not provide receipts use a page and record the information listed above. Put the page along with the packs in a zip lock bag and write the area and the shop ID on the bag with a permanent marker.
f. Move to the next shop and ask for ST products other than purchased at previous shop.
g. Put stickers, write price, put in zip lock bag and put the IDs.
    i. If unique packs, other than previously purchased, are not available then move to the next shop.

For peer review only

As **two or more teams** will be collecting data simultaneously at **different locations**. Each team will purchase the available ST products. During **In-house assessment** of the products **duplication** will be removed.

Coding of each unique product will be done during In-House assessment.

In case if the shop is closed, no consent is given, do not sell STor do not sell ST to strangers, move to the next shop. Ask each shop for new products. If you find a new product, purchase and label it. Otherwise, don’t buy duplicate product brands.

Equipment needed for field collection

Shoulder Bags (For carrying products)	Rubber bands
Zip Lock Bags	Stickers
Pens	Cash
Writing Pads	Umbrella

Data entry

An excel sheet will be given to the Data collectors. This sheet will contain information regarding the mapping of POS. It will be filled by Data collectors accordingly and will be submitted to the field supervisors on daily basis.

For peer review only

Appendix 2

Data fields for the digital ASTRA SLT product inventory form*

a) Study site________ (P1- P2)(B1-B2) (I1-I2)

b) Product Type_______(01-18)

c) Product unique ID________________ (Study site/Product Type/product number)

d) Name of brand__________________
E.g. Bacha Naswar, JM Gutka …………..

e) Packaged ____________________ (Yes/No)

f) Hard/soft pack/sachet _________________ (coding 1-4, 4 for other)

g) Country of make_______________ (coding 0 -195) (0 = Unknown)

h) Price (in local currency)_________________ (number)

i) Type of product ______________(coding 1-18, 18 for other)

j) Analyses status ____________ (Yes/No)

k) Price mentioned on pack_______________(Yes/No)

l) Tax detail mentioned on pack_______________(Yes/No)

If yes

m) How much amount has been mentioned ___________

*Adapted from the TPackSS online product inventory/registration form, available at https://globaltobaccocontrol.org/tpackss/

For peer review only

Appendix 3

In-house assessment of SLT products tool* (Adapted from TAPS guidelines)

•

**1. PACKAGING & LABELLING** (Yes =1, No = 0)	Cigarettes	Cigar/ Cigarillo	Beedi	Hukaah/shees [illegible]	Naswar	Gutka	Paan/ Betel [illegible]	Tobacco Gum	Mawah	Zarda	Gul	Snus	Nasal Snuff	Khaini	Others
a) Brand Name															
b) Name of the manufacturer only															
c) Address of the manufacturer only															
d) Name & Address of the manufacturer															
e) Contents listed (Yes/No)															
f) Weight per content is given (Yes/No)															
g) Country of Origin															
h) Statements of health claims															
i) Statements of harm e.g. harm to babies in the womb.															
j) Written health warnings (Yes/No)															
k) The language of health claims, Harm, Warnings (if No skip to k)															
I. Regional language															
II. National language															
III. English															
IV. Another foreign language															
l) Pictorial Health warning (Yes/No)(if No then skip to "m")															
m) The proportion of package does it cover															
I. 25%															

For peer review only

II. 50%															
III. 75%															
IV. 100%															
n) Provision of quit information on packaging (Yes/No)															
o) Use of culturally specific references (such as special images, symbols, or colours, etc.) (see your law or speak to local advocates for details)															
p) Presence of double packs															
q) Use of words that suggest added flavour (Yes/no)															
r) Use of words that suggest reduced strength (Yes/no)															

**2. PRICE AND TAXATION** (Yes =1, No = 0)	Cigarettes	Cigar/ Cigarillo	Beedhi	Hukaah/sheesha	Naswar	Gutka	Paan/ Betel Quid [illegible]	Tobacco Gum	Mawah	Zarda	Gul	Snus	Nasal Snuff	Khaini	Others
In what form is the product sold?															
a) Loosely sold?															
b) Loose, Mixed with other products (custom made)															
c) Packaged or wrapped by the retailer															
d) Pre-packaged by manufacturer															
e) If pre-packed then in the form of															
I. Packet															
II. Tin															
III. Bottle															
IV. Can															
V. Sachet															
VI. Box															

For peer review only

VII. If other please specify															
VIII. With branding															
f) The minimum unit of sale															
I. By quantity															
II. By weight															
g) Price of Unit customer pays															
h) Price of Unit retailer pays															

*this is the general tool. Countries are allowed to add any country specific provisions into the items.

For peer review only

Appendix 4
POS observation tool

ASTRA - POINT OF SALE OBSERVATION TOOL (Adapted from STARS)

1. Date of visit:__/__/____ Start Time: __:__ End Time: __:__
2. Coder Name/ID:________________________
3. Point of Sale ID:_____/ _______ (Study site code/serial number)
4. Store Type: (Choose one)
- a) General/Departmental Store ☐
- b) Petrol pump/Gas station store ☐
- c) Beer or Liquor store ☐
- d) Grocery store ☐
- e) Exclusive Tobacco shop ☐
- f) Tea Stall ☐
- g) Mobile cart ☐
- h) Stationary cart ☐
- i) Other (specify e.g. Discount shop):____

5. Can you survey this store? [If not, then select an option below and STOP.]
- a) Yes, I can ☐
- b) No, store does not exist ☐
- c) No, store is closed ☐
- d) No, environment unsafe for me ☐
- e) No, asked to leave before completing the survey ☐
- f) Other (specify):____

SECTION 1: OBSERVATION (POS display for mobile or stationary cart should be considered exterior)

6. EXTERIOR

Are there any point-of-sale displays of the tobacco products **OUTSIDE** the shop? (Yes = 1, No =0)	Cigarettes	Cigar/ Cigarillo	Beedi	Hukaah/sheesha	Naswar	Gutka	Paan/ Betel Quid [illegible]	Tobacco Gum	Mawah	Zarda	Gul	Snus	Nasal Snuff	Khaini	Others
a) Shelf edging															
b) Dummy packs															
c) Display packs															
d) Display stands															
e) Any other types of advertising?															

7. Is the point of sale vendor within _________meter (average distance) vicinity of any educational institution?

8. Does store display a health warning sign (OUTSIDE) against tobacco use? (If No skip to 9) ☐

Products	Written	Graphical

a) Smoking tobacco		
b) Smokeless Tobacco		

INTERIOR

9. Does the store have a pharmacy counter? (Yes = 1, No =0) ☐

10. Does store display a health warning sign (INSIDE) against tobacco use? (If No skip to 11) ☐

Products	Written	Graphical
a) Smoking tobacco		
b) Smokeless Tobacco		

11.

Are there any point-of-sale displays of the tobacco products **INSIDE** the shop? (Yes = 1, No =0)	Cigarettes	Cigar/ Cigarillo	Beedi	Hukaah/sheesha	Naswar	Gutka	Paan/ Betel Quid	Tobacco Gum	Mawah	Zarda	Gul	Snus	Nasal Snuff	Khaini	Others
a) Shelf edging															
b) Dummy packs															
c) Display packs															
d) Display stands															
e) Any other types of advertising?															

12.

**PRODUCTS OBSERVATION** (Yes = 1, No = 0)	Cigarettes	Cigar/ Cigarillo	Beedi	Hukaah/sheesha	Naswar	Gutka	Paan/ Betel Quid	Tobacco Gum	Mawah	Zarda	Gul	Snus	Nasal Snuff	Khaini	Others
a) Sold here?															
b) Visible															
c) Loosely sold here?															
d) Loose, mixed with other products (custom made)															
e) Product within 1 feet range of confectionaries, toys, candy, gum, slushy/soda machines, or ice cream?															
f) Products within 3 feet range of floor?															
g) Any price promotions/Credit?															

For peer review only

h) Cross-product promotion with tobacco? (Give any other related product free on purchase of the mentioned product)															

13. SALES TO/BY MINORS*

(Yes =1, No = 0)	**Smoking tobacco**	**Smokeless tobacco**
a) Did you observe any sales assistants under the age of 18 years in the POS?		
b) If yes, what are their expected ages? Please list.		
c) Did you observe the vendor was selling any tobacco product to young people under the age of 18?		

If you observe sale of ST to/or by a potential minor please make an effort to ask their age for confirmation.

For peer review only

Appendix 5

POS vendor interview tool

(The products in the columns and their unit of sales will be revised after the inventory study)

14. PRICES & Sale		Cigarettes	Cigar/ Cigarillo	Beedhi	Hukaah/sheesha	Naswar	Gutka	Paan/ Betel Quid with tobacco	Tobacco Gum	Mawah	Zarda	Gul	Snus	Nasal Snuff	Khaini	Others
a) Unit price that customer pay (in local currency)																
b) Unit price that retailer pay																
c) Sales Tax (in local currency)																
d) Total number of units sold per day	By Quantity															
	By Weight															
e) f) How was the price obtained																
I. Cashier/ owner/ shop keeper																
II. Advertised price																

15. SUPPORT FOR VIABLE ALTERNATIVES

Why do you sell tobacco products? Tick all that apply. (Options should not be disclosed until the shopkeeper answer. Tick all that apply and then probe with the options remaining)	**Smoking tobacco products**	**Smokeless tobacco products**
a) Good profit margin		
b) Products are in demand		
c) Family business		
d) No other viable alternative		
e) Brings people into my shop to buy other things		
f) Other reasons (please state)		
Would you ever consider stop selling SLT? (Yes = 1, N0 = 2)		
If yes, for which of the following reasons? Tick all that apply (if No skip to 16) (Options should not be disclosed until the shopkeeper answer. Tick all that apply and then probe with the options remaining)		
I. A reduction in profit margin		
II. A reduction in demand		
III. Stricter regulations and inspections		
IV. Limited supply		
V. A viable alternative		
VI. Awareness about its harms		
VII. Other reasons (please state)		

For peer review only

16. SUPPLIES

Where do you get your supplies? (Tick all that apply)	Cigarettes	Cigar/ Cigarillo	Beedhi	Hukaah/sheesh	Naswar	Gutka	Paan/ Betel Quid	Tobacco Gum	Zarda	Mawah	Gul	Snus	Nasal Snuff	Khaini	Others
a) Wholesalers (Cash and carry)															
b) Manufacturers															
c) Importers															
d) Unknown source															
e) Another shop/ store															
f) Others (Please state)															

Why do you buy this product from a particular supplier? (Tick all that apply)	Cigarettes	Cigar/ Cigarillo	Beedhi	Hukaah/sheesha	Naswar	Gutka	Paan/ Betel Quid	Tobacco Gum	Mawah	Zarda	Gul	Snus	Nasal Snuff	Khaini	Others
a) They are the only supplier															
b) They can supply the required amount															
c) They are cheap															
d) They are nearby															
e) They are a friend or family member															
f) They sell products or brands my customers want															
g) They give me incentives															
h) Another reason (please state)															

What is the nature of the incentive by a particular supplier? (Tick all that apply)	Cigarettes	Cigar/ Cigarillo	Beedhi	Hukaah/sheesha	Naswar	Gutka	Paan/ Betel Quid	Tobacco Gum	Mawah	Zarda	Gul	Snus	Nasal Snuff	Khaini	Others
I. Discount															

For peer review only

II. Promotional Goods															
III. Point-of-sale displays															
IV. Free sample															
V. Others (please state)															

Where are your suppliers based (Tick all that apply)	Cigarettes	Cigar/ Cigarillo	Beedhi	Hukaah/sheesha	Naswar	Gutka	Paan/ Betel Quid	Tobacco Gum	Mawah	Zarda	Gul	Snus	Nasal Snuff	Khaini	Others
a) Local															
b) Abroad															
c) If Abroad, Where?															
d) Do you have contact details of the supplier?															
e) If "d" Yes, Can you Provide the contact details															
f) If "e" Yes, Contact Details of suppliers	**(Type of Product)**														
	**(Type of Product)**														
	**(Type of Product)**														
	**(Type of Product)**														

For peer review only

17. TOBACCO ADVERTISING, PROMOTION, AND SPONSORSHIP

(Yes =1, No = 0)

How do you (retailer) try to retain your tobacco customers? Tick all that apply.	**Smoking tobacco**	**Smokeless tobacco**
a) Offer discounts		
b) Offer promotional goods		
c) Offer credit lines		
d) Good customer relationships		
e) Other ways (please state)		

18. SALES TO/BY MINORS

(Yes =1, No = 0)	Smoking tobacco	Smokeless tobacco
d) Do you have any sales assistants under the age of 18 years?		
e) If yes, what are their ages? Please list.		
f) Are they allowed to sell tobacco products?		
g) Do you ever sell any tobacco products to young people under the age of 18?		
h) If no, why not?		

PROFIT	Amount in local currency
a) Roughly what amount of profit do you make from daily sales (Overall)	
b) What amount of profit comes from daily cigarettes sale	
c) What amount of profit comes from daily smokeless tobacco sale	

19. EDUCATION AND PUBLIC AWARENESS

(Yes =1, No = 0)

Are you aware of any laws that impact your business of selling tobacco products?	Smoking tobacco	Smokeless tobacco
a) If yes, please state which law(s) and how it affects your business		
**Are you visited by any inspector or regulator to inspect and/or test your tobacco products?**	**Smoking tobacco**	**Smokeless tobacco**
a) Inspect products		
b) Test products		
c) Check your license or registration		
d) How many visits did you have in the last 3 years?		
e) What happens after the inspection?		
I. If found not in compliance, nothing happens		
II. If found not in compliance, there is a penalty		
III. If found in compliance, nothing happens		
IV. If found in compliance, you are given a certificate		
V. Any other consequences?		
Do you have to disclose the contents of smokeless tobacco products to any statutory body?		
Do you have to send the products to a laboratory, for testing after Inspection?		

Signature of the interviewer___________________________

For peer review only

BMJ Open

Compliance of smokeless tobacco supply chain actors and products with Tobacco Control laws in Bangladesh, India and Pakistan: Protocol for a multi-centre sequential mixed-methods study.

Journal:	*BMJ Open*
Manuscript ID	bmjopen-2019-036468.R2
Article Type:	Protocol
Date Submitted by the Author:	11-May-2020
Complete List of Authors:	khan, Zohaib; Khyber Medical University<br>Huque, Rumana; University of Dhaka, Economics<br>Sheikh, Aziz; University of Edinburgh, Division of Community Health Sciences<br>Readshaw, Anne; University of York Department of Health Sciences<br>Eckhardt, Jappe; University of York<br>Jackson, Cath; University of York, York Trials Unit<br>Kanaan, Mona; University of York, Health Sciences<br>Iqbal, Romaina; Aga Khan University, Department of Community Health Sciences and Medicine<br>Akhter, Zohaib; Aga Khan University<br>Garg, Suneela; Maulana Azad Med Coll, Community medicine<br>Singh, Mongjam; Maulana Azad Medical College<br>Ahmad, Fayaz; Khyber Medical University<br>Abdullah, S M; University of Dhaka Department of Economics<br>javaid, arshad; Khyber Medical University<br>A.Khan, Javaid; Aga Khan University, Department of Pulmonary medicine<br>Han, Lu; University of York Department of Health Sciences<br>Rahman, Aziz; Federation University Australia Faculty of Health<br>Siddiqi, Kamran; University of York, UK, Institute of Health Sciences
**Primary Subject Heading**:	Global health
Secondary Subject Heading:	Global health, Health policy
Keywords:	PUBLIC HEALTH, QUALITATIVE RESEARCH, STATISTICS & RESEARCH METHODS

SCHOLARONE™
Manuscripts

I, the Submitting Author has the right to grant and does grant on behalf of all authors of the Work (as defined in the below author licence), an exclusive licence and/or a non-exclusive licence for contributions from authors who are: i) UK Crown employees; ii) where BMJ has agreed a CC-BY licence shall apply, and/or iii) in accordance with the terms applicable for US Federal Government officers or employees acting as part of their official duties; on a worldwide, perpetual, irrevocable, royalty-free basis to BMJ Publishing Group Ltd ("BMJ") its licensees and where the relevant Journal is co-owned by BMJ to the co-owners of the Journal, to publish the Work in this journal and any other BMJ products and to exploit all rights, as set out in our licence.

The Submitting Author accepts and understands that any supply made under these terms is made by BMJ to the Submitting Author unless you are acting as an employee on behalf of your employer or a postgraduate student of an affiliated institution which is paying any applicable article publishing charge ("APC") for Open Access articles. Where the Submitting Author wishes to make the Work available on an Open Access basis (and intends to pay the relevant APC), the terms of reuse of such Open Access shall be governed by a Creative Commons licence – details of these licences and which Creative Commons licence will apply to this Work are set out in our licence referred to above.

Other than as permitted in any relevant BMJ Author's Self Archiving Policies, I confirm this Work has not been accepted for publication elsewhere, is not being considered for publication elsewhere and does not duplicate material already published. I confirm all authors consent to publication of this Work and authorise the granting of this licence.

For peer review only

Compliance of smokeless tobacco supply chain actors and products with Tobacco Control laws in Bangladesh, India and Pakistan: Protocol for a multi-centre sequential mixed-methods study.

Khan Z[1], Huque R[2,3], Sheikh A[4], Readshaw A[5], Eckhardt J[6], Jackson C [5,7], Kanaan M[5], Iqbal R[8], Akhter Z[8], Garg S[9], Singh MM[9], Ahmad F[1], SM Abdullah[2,3], Javaid A[1], Khan J[8], Han L[5], Rahman MA[10], Siddiqi K[5].

Authors' affiliations:

[1]Khyber Medical University, Pakistan; [2]ARK Foundation, Bangladesh; [3]Department of Economics, University of Dhaka, Bangladesh; [4]Usher Institute, University of Edinburgh, UK; [5]Department of Health Sciences, University of York, UK; [6]Department of Politics, University of York, UK; [7]Valid Research Ltd, Wetherby UK, [8] The Aga Khan University, Pakistan; [9]Maulana Azad Medical College, New Delhi, India; [10]Federation University, Australia.

Corresponding author: Dr. Anne Readshaw

Email: anne.readshaw@york.ac.uk

Abstract

Introduction

South Asia is home to more than 300 million smokeless tobacco (ST) users. Bangladesh, India, and Pakistan as signatories to the Framework Convention for Tobacco Control (FCTC) have developed policies aimed at curbing the use of tobacco. The objective of this study is to assess the compliance of ST point-of-sale (POS) vendors and the supply chain with the articles of the FCTC and specifically with national tobacco control laws. We also aim to assess disparities in compliance with tobacco control laws between ST and smoked tobacco products.

Methods and Analysis

The study will be carried out at two sites each in Bangladesh, India, and Pakistan. We will conduct a sequential mixed-method study with five components: i. Mapping of ST POS, ii. Analyses of ST samples packaging, iii. Observation, and iv. Survey interviews of POS, and v. In-depth interviews with wholesale dealers/suppliers/manufacturers of ST. We aim to conduct 900 POS survey interviews and observations, and 3-5 in-depth interviews at each study site in the three countries. Data collection will be done by trained data collectors. The main statistical analysis will report the frequencies and proportions of shops that comply with the FCTC and local tobacco control policies, and provide a 95% confidence interval of these estimates. The qualitative in-depth interview data will be analysed using the framework approach. The findings will be connected, each component informing the focus and/or design of the next component.

Ethics and dissemination

Ethical approvals for the study have been received from the Health Sciences Research Governance Committee at the University of York, UK. In-country approvals were taken from

For peer review only

the National Bioethics Committee in Pakistan, the Bangladesh Medical Research Council, and the Indian Medical Research Council. Our results will be disseminated via scientific conferences, peer-reviewed research publications, and press releases.

Keywords: Smokeless tobacco, South Asia, point of sale, tobacco control, Bangladesh, India, Pakistan

Word Count: 3999

Strengths and limitations

- This will be the first multi-country study on the smokeless tobacco supply chain in South Asia that aims to map smokeless tobacco points of sale using geographical information systems.
- Building upon the methods used by the global TPackSS project, our proposed study will develop/update the inventory of smokeless tobacco products available in Bangladesh, India, and Pakistan.
- Purposive sampling of our main study sites may decrease the external validity of our methods and results.
- Owing to a fear of the relevant authorities, the answers to our survey questions on taxes and finances might not be completely accurate.
- We will not be able to do a regression analysis including shop characteristics for the pack analysis component, as we are only collecting unique packs and not all packs from each shop

Introduction

Smokeless tobacco (ST) refers to all tobacco products that are used without burning the product [1]. The International Agency for Research on Cancer estimates that ST contains more than 30 carcinogens [2]. The use of ST is associated with an increased incidence of head, neck and gastrointestinal cancers [3-5]. Additionally, ST use is associated with Oral Potentially Malignant Disorders (OPMDs) [6] and a variety of other oral and systemic diseases [7, 8]. An estimated 300 million people around the globe use ST products in some form, and the majority (≥85%) of these ST users live in South Asia [1, 9]. The use of ST products is particularly common in India, Bangladesh, and Pakistan, where it is considered an acceptable social norm [10]. This situation is further complicated by the presence of high amounts of Tobacco Specific Nitrosamines, the main cancer-causing agents in tobacco, in the products available in South Asia, compared to the rest of the world [11].

The Framework Convention for Tobacco Control (FCTC) is a global treaty that aims to enhance tobacco control measures and incorporate the provisions of the framework into their national tobacco control laws [12]. Although the FCTC is aimed at curbing all types of tobacco use, the focus of tobacco control in most countries has been on controlling smoking, with control of the non-smoking forms of tobacco often neglected [13, 14]. The 2016 Global Progress Report on the implementation of FCTC highlighted gaps in the formulation and implementation of ST control policies in most parties to the FCTC [15]. Bangladesh, India, and Pakistan are signatories to the FCTC and have made variable progress in implementing the articles of the framework by developing national tobacco control policies [16].

For peer review only

The tightening up of smoking regulations has been associated with a reported shift from smoking to ST use in some South Asian countries [9, 17]. The increase in ST use can be attributed to a variety of factors, including, but not limited to cultural acceptance, perceived safety of ST products compared to smoking, harm-reduction, low prices, and lax or absent ST control laws [14].

A 2018 report from the Global Knowledge Hub on Smokeless Tobacco indicated progress being made with regards to ST control policies and regulations in South Asia [14]. Many of these regulations and laws are aimed at demand reduction via various interventions at the tobacco points of sale e.g. removal of tobacco products displays, and restrictions and bans on the advertisement of tobacco products at the point of sale [18, 19]. The success of these regulations and laws in curbing the tobacco epidemic in South Asia largely depends on the degree of compliance with the implementation of these laws and regulations [20]. However, the evidence on the implementation of the ST control laws and regulations at the point of sale (POS) in the context of India, Bangladesh, and Pakistan is scarce. Most of the previous work carried out in these countries focused on tobacco advertisement and promotion at the POS and health warnings on ST packaging [21-26], with little emphasis on the other provisions of the FCTC, such as sale to/by minors, price and taxation, and provision of viable alternatives. As such, there is a need for a comprehensive assessment of the compliance of POS in India, Pakistan, and Bangladesh with the local ST control policies and the specific articles of the FCTC. `

This multi-country sequential mixed-methods study aims to provide a comprehensive assessment of compliance of ST POS and products with the tobacco control laws in our three target countries. Furthermore, we aim to elicit information on any disparities between the implementation of FCTC/local laws for ST and smoking. We will also develop a country-specific inventory of tobacco products, to gain insight into the diversity of the products available in the local market, as well as identifying illicit products. Additionally, we will assess the barriers and facilitators to compliance of the ST wholesale dealers and suppliers with the different provisions of the FCTC/local laws.

Methods and Analysis

Study design

We will conduct a sequential mixed-methods study, incorporating quantitative work (geo-mapping of ST POS, in-house analyses of ST products, POS survey interviews and observations and qualitative (in-depth-interviews with supply chain actors e.g. wholesale dealers, suppliers) work. The findings from each component will be connected and used to inform the focus and/or the design of the next component i.e. mapping of the POS will allow us to build a sampling frame from which we will randomly choose survey interview respondents. The ST pack analyses will provide us an insight into manufacturing and sale practices that can be incorporated into the POS survey interview tool and the topic guide for in-depth interviews. The findings from the survey interviews and observations of the POS will guide the development of country-specific topic guides for the in-depth Interviews.

Study sites

We will conduct this study in Bangladesh, India and Pakistan. In each country, we have purposively selected two sites based on the use and diversity of the available ST products. In India, the study will be conducted in the North-East and North-West districts of Delhi. In Bangladesh, the study will be conducted in the districts of Dhaka and Rangpur. In Pakistan, the

For peer review only

study will be conducted in the districts of Karachi and Peshawar. In Pakistan and Bangladesh, the selected sites are the hubs of ST use and manufacture, for India, our rationale for choosing the sites was based on Delhi being a metropolitan city with a diverse population and products and also the logistics available to study team.

Study duration

The data collection will be carried out from June 1, 2019, to December 31, 2020, and analyses will be completed by March 30, 2021.

General sampling strategy

We will use a multi-stage mixed sampling technique for the study (Fig. 1).

Stage 1: Within each of the selected administrative areas (District/Division), we will purposively select one predominantly urban and one predominantly rural sub-district. Our study sites included districts/areas, which comprise urban, peri-urban, and rural areas. We will use the available local government documents, latest census and expert opinion of local researchers to identify two sub-districts/areas at each site, one predominantly urban and the other predominantly rural/peri-urban.

Stage 2: We will randomly select three smaller administrative units per sub-district. These will be our Primary Sampling Units (PSU).

Stage 3: From each PSU, we will randomly select two enumeration blocks/neighbourhood areas/villages; these will be our Secondary Sampling Units (SSU).

Data collection (General considerations)

At each study site, we will deploy a team of trained data collectors to administer the quantitative data collection instruments (comprising the geo-mapping, in-house analysis of the ST products packaging, structured POS vendor survey interview and observation components). Data collectors will be trained on how to administer the various study tools beforehand. They will also receive training in obtaining informed and written/verbal consent. Calibration of the product in-house analysis component will be done via video conferencing involving study teams from all sites. A qualitative research specialist will train one person at each participating site to conduct and transcribe the in-depth interviews with the suppliers and/or manufacturers. Data will be monitored for quality and completeness by the site leads/study leads/principal investigator. We will also conduct small pilot studies in each country to field-test our study tools.

Data management

Data will be initially collected on a paper-based questionnaire and then transferred to a central database kept on a secure server at the University of York. Each ST product, POS vendor, supplier, and manufacturer recruited in the study will be allotted a unique identification number and the recorded data will not contain any identifiable information about them. Only the study leads/principal investigator/study statistician will have access to the database and data collection tools. All filled questionnaires will be stored in locked filing cabinets at the collaborating institutions in each of the study centres.

In line with the General Data Protection Regulations (GDPR) of the European Union (EU) and the Research Governance Framework for Health and Social Care Research, data will be

For peer review only

securely archived by the University of York for a minimum of five years after the completion of the study. Following authorisation from the sponsor, arrangements for confidential destruction will then be made. Recordings from the in-depth interviews will be deleted at the end of the study. If an individual withdraws consent for their data to be used, it will be destroyed without breach of confidentiality.

Study component-specific methods

POS Geo-Mapping

Eligibility criteria for POS

A POS will be eligible for inclusion in the study if it sells any form of ST. The common types of tobacco POS that might be eligible for our study are:

a. General/departmental stores
b. Petrol pump/gas station stores
c. Beer or liquor stores
d. *Paan* (betel-quid) shops
e. Grocery stores
f. Exclusive tobacco shops
g. Discount shops
h. Mobile vendors/carts
i. Stationary carts
j. Country-specific additional POS types.

Identifying and mapping eligible POS

To identify the eligible POS, we will employ standard approaches for tobacco POS audits [27]. This will entail:

i. Developing a list of tobacco sellers in the selected areas by collecting the details of local vendors who have a license to sell tobacco products (if applicable), and/or the acquisition of general lists of local businesses/sellers from trade unions and/or local administrative authorities and combing potential tobacco POS from these lists.
ii. This will be followed by "ground-truthing" by trained data collectors, who will visit these shops to ascertain, a. The existence of the POS, and b. The sale of ST products at these POS.
iii. Once ascertained, each eligible POS will be allotted a unique identification number and its location will be recorded via the Google Maps® digital application. The application gives its users their current location on Google Maps, the Global Positioning System (GPS) coordinates, and the geolocation status.
iv. If no prior lists (license holders or businesses/sellers) are available at the study location(s), our data collection team will do "canvassing" of the ST POS by walking along all the primary and secondary roads in that area and identifying eligible ST POS (Appendix 1).
v. Additionally, we will request each ascertained POS vendor to identify other ST POS, which will be added to the list.
vi. If data collectors observe a mobile vendor during the ground-truthing or canvassing at the study sites, the area where the team establishes the first contact with that vendor will be marked as his/her location.

For peer review only

Measuring the density of ST POS

We will collect the most recent official population census data of our selected study sites from the relevant department(s) in each country. If such data are not available, we will use unofficial estimates gathered from the local administration. The density of the POS will be calculated per 1,000 population at our study sites.

Measuring the distance of the POS from schools

We will acquire the list of all schools along with their GPS coordinates, situated in the selected SSUs from the local education departments. If the data are not available, our team will visit each school physically, drop a Google Maps pin at the school gate and record the coordinates. We will plot all the ST POS and the schools on a digital map to assess if there is compliance with the local laws that ban the sale of tobacco products in the vicinity (distance will vary according to country) of the schools.

ST products in-house analysis

Eligibility criteria

All brands of ST products available in the markets at the study sites will be procured. For a product to be eligible, it must contain tobacco. The collection of the ST products will be based on the brand names and not the generic product e.g. all brands of *Gutkha* available in the local market will be collected. Loose products that are not sold in packaging will be eligible, but only one generic sample of the product will be procured. The most commonly sold brand/make of cigarettes, cigars, cigarillos, and beedi according to the selected vendor, will be procured.

Sample collection and storage

All ST sale shops/vendors identified in the selected SSUs by the geo-mapping exercise will be approached to procure the ST products that are available in the local markets. We will employ an adapted form of the unique packs sampling technique used for the TPackSS samples collection [28]. One of every unique pack of ST (any pack with at least one difference in an exterior feature of the pack including size, brand name, presentation, colours, cellophane, packaging material (i.e. hard, soft, tin), and the inclusion of a promotional item) available in the store will be purchased. This sampling will begin at a randomly-selected eligible vendor. After the purchase at the initial vendor, at each of the remaining eligible vendors, one of every ST pack that was not already purchased at a previous outlet will be purchased. If the selected vendor does not have any new packs (packs that were not purchased at another outlet), the sample collection team will move on to the next eligible vendor. The sample collection will continue until the team has approached the last eligible vendor. The sample collection team will ascertain the retail price of each procured pack from the vendor. They will also ascertain if the prices are inclusive of tax or not. The sample collection team will neither request nor accept a reduced price, in case the vendor offers a discount due to the large quantity being purchased, other than in those cases where discounted products are already being offered to all customers as a part of a promotion. All such general promotions, and individual "reduced price" offers will be recorded on a field notes sheet provided to each data collector.

Product analyses

All the procured samples will be sorted by product type, and each sample will be allotted a unique identification number marked on the Ziploc bag with indelible ink. The study team at each participating institution will analyze one sample at a time, carefully locking it back in the bag before moving onto the next sample. A physical inventory of all the products will be developed at each site and the data related to the products e.g. unique identification number,

For peer review only

product family, purchase price and date of purchase will be fed into a digital inventory form (Appendix 2) adapted from the TPackSS inventory data entry form.

The in-house assessment of the ST products will involve assessment and recording of their size, price, tax disclosure, ingredient lists, health warnings, manufacturer details, etc. Our study tool (Appendix 3) has been adapted from the TAPS standard tool. We will collect the samples by brand names and assess whether disparities exist between ST product categories and/or different manufacturers. We will also collect data on the country of manufacture and analyze if differences in compliance with the relevant laws can be explained by differences in the country of manufacture and/or "smuggled/illicit" designation of the product. The definition of illicit products for each participating site will be developed after consulting local stakeholders. We will also analyze the collected cigarette packs, to assess their compliance with the local laws/FCTC. This will also help us in the identification of any disparity in the local laws for ST and those for cigarettes.

For peer review only

POS Survey Interviews and Observation

Sample size

For the POS observation and Interviews, we calculated sample sizes for the individual countries based on the prevalence of non-compliance with specific provisions of the local tobacco control laws in the three countries. The point estimates were derived from published literature i.e. 20% of shops selling tobacco to minors in Pakistan [29], 19.4% POS non-compliant with COTPA regulations regarding sizing of the advertisements in India [30], and 20% POS non-compliant with tobacco advertisement and promotion laws in Bangladesh [31].

Using the formula sample $\boldsymbol{n}$ **= [DEFF*Np(1-p)]/ [(d^2/Z$^2_{1-\alpha/2}$*(N-1)+p*(1-p)],** and assuming a 20% prevalence of non-compliance, a confidence interval of 95%, and an absolute precision of 7%, the sample size for each country came out to be 251. After accounting for a 15% non-response (based on our ST supply chain study in Pakistan and Bangladesh) [32], the sample size was rounded up to 290 per country. All sample size calculations were carried out in Open Epi software [33].

Sampling strategy

We will follow the general sampling strategy (Fig 1). Once a sampling frame of eligible ST POS in each SSU has been developed through the geo-mapping, a random sample of the 13 POS vendors will be recruited from each SSU.

Recruitment

After providing brief information about the study to the person-in-charge of the POS at the time of the store observation, we will formally ask them to provide written consent to participate in both the POS observation and POS vendor interview components of the study. Those who do not agree to participate after receiving the study information will not be enrolled in the study. The reasons for declining to participate in the study will be recorded. Following consent, the data collector will proceed to data collection. The consent provider, who could be either the POS owner (not land-lords), POS manager or sales assistant(s) will be eligible for an interview.

Data collection and tools

An adapted version (Appendix 4) of the Standardized Tobacco Assessment for Retail Settings (STARS) tool [34], will be used to record the observations about product promotion, advertisement, and health warnings, etc. We will observe both the exterior and interior of the shops to assess compliance with FCTC/local laws. The tool consists of data fields on health

warnings inside and outside the shop, product advertisement, promotions, display shelves, dummy packs, product placement, products sold as confectioneries, and cross-promotion. We will also collect similar data for cigarettes and other smoking products, for comparison.

Our interview tool (Appendix 5) has been adapted from a survey instrument that has already been field-tested in the UK, Bangladesh, Nepal, and Pakistan [32]. The tool will be translated into local languages at each study site. In addition to a series of close-ended questions on ST products regarding their supply, sale, popularity, prices, profits, and taxes, the tool also includes similar questions for cigarettes, to allow for comparative analysis. We will collect data on mediating/moderating variables that could act as barriers/facilitators to the increased implementation of the FCTC/local laws. These variables are awareness of local tobacco control legislation, standards, and obligations, awareness of adverse health effects of ST, the effect of health warnings on sales, market share of SLT products, overall sales related to the availability of tobacco products for non-exclusive vendors, incentives by manufacturers/suppliers, profit margins, regular inspections, penalties for non-compliance, and availability of viable alternatives.

Data analysis

The main statistical analysis will report the frequencies and proportions of shops that comply with the WHO FCTC and local tobacco control policies, as measured by the different items on the survey questionnaire, and provide a 95% confidence interval of this estimate. We will also provide summaries for the other categorical variables using frequencies and percentages and for continuous variables, we will provide means and standard deviations in addition to a graphical representation. Medians and interquartile ranges will also be computed if the data are skewed.

We will use logistic regression to explore the bivariate association of compliance with shop characteristics, such as the type of shop, adjusting for the study design. We will further expand these regression models to explore the association of compliance with multiple explanatory variables adjusting for the study design. Where possible, figures related to ST sales will be compared with those of cigarette sales, using similar approaches to the above. We will consider a *p*-value of less than 0.05 to be statistically significant. A detailed statistical analysis plan will be developed before the main analysis, which will include any *a priori* subgroup analyses and strategies for handling missing data. We will use STATA 15 for analysis.

In-depth Interviews (Supply chain)

Participants, sampling and recruitment

At each of the six study sites, we will identify, and collect contact details of, at least five supply chain actors (from manufacturers, wholesalers, suppliers) with the help of the POS survey respondents. From this list, we will purposively recruit 03-05 participants at each site, ensuring that the sample includes at least one manufacturer and one supplier from each site.

Data collection

A topic guide will be developed for the interviews. The contents of the guide will be based on the findings from the POS survey. That is, the responses in the survey that warrant further exploration will be identified by the study teams in each country and the topic guide aimed at eliciting further information on the responses will be developed by the qualitative research team. We will start with a standard topic guide and then tailor it to the findings from the different countries

For peer review only

The interviews will be conducted at locations that are easily accessible to participants, and where they are confident to speak openly, such as their workplaces or the offices of ward/union council office bearers or the village chief's house/hujra. Each interview will last 30-60 minutes. A trained researcher will conduct the interviews in the local language. All interviews will be audio recorded.

Data analysis

The interviews will be transcribed verbatim, translated into English and analysed using the Framework approach [35]. The framework approach is not associated with a particular epistemological viewpoint or theoretical approach and, as such, is not necessarily concerned with generating social theory. The approach can be used in inductive or deductive analysis or a combination of the two [36]. We will be using an inductive approach and develop a framework matrix, informed by the study objectives, the interview topics and data from preliminary interviews. The data from the three countries will be charted into the same matrix; and then reviewed and interrogated to compare and contrast views, seek patterns, connections and explanations within the data, including comparison across the three countries. Excel software will aid data handling.

Patient and public involvement

The study is supported by stakeholder groups in each participating country. The stakeholder groups consist of health professionals, policy-makers, representatives from general society, students and influencers. The stakeholder groups meet once every year, and so far have been involved in the design of the study tools, refinement of research questions and data collection methods. These groups will also be involved in the dissemination of findings from the study.

Ethics and Dissemination

We will adhere to the fundamental principles of human rights as per the Helsinki Declaration. Ethical approval has been obtained from the University of York "Health Sciences Research Governance Committee". Additionally, each participating South Asian institution in ASTRA has obtained ethical approvals from their institutional/national ethics bodies (Supplementary file, appendix 6). All potential participants will be provided with information regarding the project and written/verbal consent will be taken from each participant before recruitment. Consent forms will contain personal details including name, date of birth, and address.

All information collected during the study will be kept strictly confidential. There will be restricted access and disposal arrangements for the participants' details. Information will be held securely on paper/electronically at the University of York. Data (other than consent forms) transferred to or from the University of York will be coded with a study enrolment number and will not have participants' identifiers. We have studied the conditions under which disclosures to law enforcement agencies are required and are satisfied that the chances of these occurring (criminal activities) are highly unlikely.

To engage our academic audience and the research fraternity, we will publish the results of the study in peer-reviewed journals. We will also present our findings at an international public health conference and an international tobacco control conference. We will also publish an abstract of our findings on the websites of all the partner institutions. We will also present our work to regional research meetings. Additionally, we will incorporate the learning from this project into academic lectures for Master students of public health. We have already established national stakeholder groups in all three target countries, who will guide the study conduct and help us in the dissemination of our findings.

For peer review only

Declarations

The authors declare no conflict of interest.

Funding

This research is funded by the UK's National Institute for Health Research (NIHR) [ASTRA (Grant Reference Number 17/63/76)]. The views expressed in this publication are those of the authors and not necessarily of the NIHR or the Department of Health and Social Care.

Authors contribution

ZK, KS, RH, SG, MMS conceived the study, wrote the first and all subsequent drafts.

AR, FA, AMR, ZA, ASM, RI, AJ, JK and AS wrote the initial drafts and commented on the final draft.

MK and LH commented on all drafts and wrote up the sampling and analyses section.

JE and CJ commented on initial and final versions of the draft and wrote up the section on mixed methods and in-depth interviews.

Figure Legend

Fig 1. General sampling strategy to be used at all sites.

a. Within each of the selected administrative areas (District/Division), we will purposively select one predominantly urban and one predominantly rural sub-district. We will use the available local government documents, latest census and expert opinion of local researchers to identify two sub-districts/areas at each site, one predominantly urban and the other predominantly rural/peri-urban.
b. We will randomly select three smaller administrative units per sub-district. These will be our Primary Sampling Units (PSU).
c. From each PSU, we will randomly select two enumeration blocks/neighbourhood areas/villages; these will be our Secondary Sampling Units (SSU).
d. From each SSU we will recruit up to 13 POS.
e. Required sample size/country is 290, through this strategy we will be able to recruit up to 312 POS.

References

1. Eriksen M, Mackay J, Ross H. The tobacco atlas. American Cancer Society; 2013.

2. IARC Working Group on the Evaluation of Carcinogenic Risks to Humans, World Health Organization, International Agency for Research on Cancer. Smokeless tobacco and some tobacco-specific N-nitrosamines: World Health Organization; 2007.

For peer review only

3. Guha N, Warnakulasuriya S, Vlaanderen J, Straif K. Betel quid chewing and the risk of oral and oropharyngeal cancers: A meta-analysis with implications for cancer control. International Journal of Cancer. 2014;135(6):1433-1443.

4. Boffetta P, Hecht S, Gray N, Gupta P, Straif K. Smokeless tobacco and cancer. Lancet Oncol. 2008;9(7):667-675.

5. Khan Z, Dreger S, Shah SMH, Pohlabeln H, Khan S, Ullah Z, Rehman B, Zeeb H. Oral cancer via the bargain bin: The risk of oral cancer associated with a smokeless tobacco product (Naswar). PloS one. 2017;12(7):e0180445.

6. Khan Z, Khan S, Christianson L, Rehman S, Ekwunife O, Samkange-Zeeb F. Smokeless tobacco and oral potentially malignant disorders in South Asia: a systematic review and meta-analysis. Nicotine and Tobacco Research. 2017;20(1):12-21.

7. Siddiqi K, Shah S, Abbas SM, Vidyasagaran A, Jawad M, Dogar O, Sheikh A. Global burden of disease due to smokeless tobacco consumption in adults: analysis of data from 113 countries. BMC Med. 2015;13:194-015-0424-2.

8. Sinha DN, Abdulkader RS, Gupta PC. Smokeless tobacco-associated cancers: A systematic review and meta-analysis of Indian studies. International Journal of Cancer. 2016;138(6):1368-1379.

9. Suliankatchi RA, Sinha DN, Rath R, Aryal KK, Zaman MM, Gupta PC, Karki KB, Venugopal D. Smokeless Tobacco Use is "Replacing" the Smoking Epidemic in the South-East Asia Region. Nicotine Tobacco Res. 2017;1:6.

10. Gupta PC, Ray CS. Smokeless tobacco and health in India and South Asia. Respirology. 2003;8(4):419-431.

11. Stanfill SB, Connolly GN, Zhang L, Jia LT, Henningfield JE, Richter P, Lawler TS, Ayo-Yusuf OA, Ashley DL, Watson CH. Global surveillance of oral tobacco products: total nicotine, unionised nicotine and tobacco-specific N-nitrosamines. Tob Control. 2011;20(3):e2.

12. Shibuya K, Ciecierski C, Guindon E, Bettcher DW, Evans DB, Murray CJ, WHO Framework Convention on Tobacco Control. WHO Framework Convention on Tobacco Control: development of an evidence based global public health treaty. BMJ. 2003;327(7407):154-157.

13. WHO Study Group on Tobacco Product Regulation. Advisory note: Waterpipe tobacco smoking: health effects, research needs and recommended actions for regulators. 2015.

14. Zaatari GS MD, Bazzi A. Impact of the WHO FCTC on non-cigarette tobacco products. Tob Control. 2018.

15. Chung-Hall J, Craig L, Gravely S, Sansone N, Fong GT. Impact of the WHO FCTC over the first decade: a global evidence review prepared for the Impact Assessment Expert Group. Tob Control. 2018.

For peer review only

16. Shang C, Lee HM, Chaloupka FJ, Fong GT, Thompson M, O'Connor RJ. Association between tax structure and cigarette consumption: findings from the International Tobacco Control Policy Evaluation (ITC) Project. Tob Control. 2018.

17. Khan Z. A gaping gap (smokeless tobacco control in Pakistan). Tobacco induced diseases. 2016;14(1):36.

18. World Health Organization. Monitoring tobacco control among youth in countries of the South-East Asia Region: 2018. 2018.

19. World Health Organization. Second joint mission of the United Nations Interagency Task Force on the Prevention and Control of Noncommunicable Diseases, Sri Lanka, 23-27 April 2018. 2018.

20. He Y, Shang C, Huang J, Cheng KW, Chaloupka FJ. Global evidence on the effect of point-of-sale display bans on smoking prevalence. Tob Control. 2018.

21. Tobacco Pack Surveillance System (TPackSS). Health Warning Label Compliance: India‐2013. Baltimore, MD: Johns Hopkins Bloomberg School of Public Health. https://www.globaltobaccocontrol.org/tpackss/sites/default/files/tpackss_smokeless_HWL_10_22_2018.pdf

22. Iacobelli M, Saraf S, Welding K, Smith KC, Cohen JE. Manipulated: graphic health warnings on smokeless tobacco in rural India. Tobacco Control. 2020 Mar 1;29(2):241-2.

23. Mullapudi S, Britton J, Kulkarni MM, Moodie C, Kamath VG, Kamath A. A pilot study to assess compliance and impact of health warnings on tobacco products in the Udupi district of Karnataka State, India. Tobacco induced diseases. 2019;17.

24. Rahman SM, Alam MS, Zubair AB, Shahriar MH, Hossein M, Alam MS, Hefler M. Graphic health warnings on tobacco packets and containers: compliance status in Bangladesh. Tobacco control. 2019 May 1;28(3):261-7.

25. Cohen JE, Brown J, Washington C, Welding K, Ferguson J, Smith KC. Do cigarette health warning labels comply with requirements: A 14-country study. Preventive medicine. 2016 Dec 1;93:128-34.

26. Tobacco Pack Surveillance System (TPackSS). Health Warning Label Compliance: Bangladesh‐2016. Baltimore, MD: Johns Hopkins Bloomberg School of Public Health. http://globaltobaccocontrol.org/tpackss/sites/default/files/Tpackss_Bangladesh_healthwarningII_090517.pdf

27. Feld AL, Johnson TO, Byerly KW, Ribisl KM. How to Conduct Store Observations of Tobacco Marketing and Products. Prev Chronic Dis. 2016;13: E25.

28. Smith K, Washington C, Brown J, Vadnais A, Kroart L, Ferguson J, Cohen J. The Tobacco Pack Surveillance System: A Protocol for Assessing Health Warning Compliance, Design Features, and Appeals of Tobacco Packs Sold in Low- and Middle-Income Countries. JMIR Public Health Surveill. 2015;1(2):e8.

For peer review only

29. CTC Pakistan. Stubbing it out. In Edited by Anonymous Pakistan: Society for Alternative Media and Research; 2017:[Anonymous].

30. Patel S, Rendell H, Maudgal S, Oswal K. Tobacco industry tactics with advertisements at the point of sale in Mumbai. Indian J Cancer. 2013;50(3):245-249.

31. Bhuiyan MEH, Ovi FH, Alam MM, Farheen I. Documentation on Tobacco Advertising, Promotion & Sponsorship in Bangladesh. 2014;ISBN: 978-984-33-6739-6.

32. Siddiqi K, Scammell K, Huque R, Khan A, Baral S, Ali S, Watt I. Smokeless Tobacco Supply Chain in South Asia: A Comparative Analysis Using the WHO Framework Convention on Tobacco Control. Nicotine Tob Res. 2016;18(4):424-430.

33. Dean A, Sullivan K, Soe M. OpenEpi: Open source epidemiologic statistics for public health, version. 2014.

34. Henriksen L, Ribisl KM, Rogers T, Moreland-Russell S, Barker DM, Sarris Esquivel N, Loomis B, Crew E, Combs T. Standardized Tobacco Assessment for Retail Settings (STARS): dissemination and implementation research. Tob Control. 2016;25(Suppl 1):i67-i74.

35. Ritchie J, Lewis J. McNaugton Nicholls C, Ormston R. (2014). Qualitative Research Practice: A guide for social science students and researchers. London: SAGE.

36. Gale NK, Heath G, Cameron E, Rashid S, Redwood S. Using the framework method for the analysis of qualitative data in multi-disciplinary health research. BMC medical research methodology. 2013 Dec;13(1):117.

For peer review only

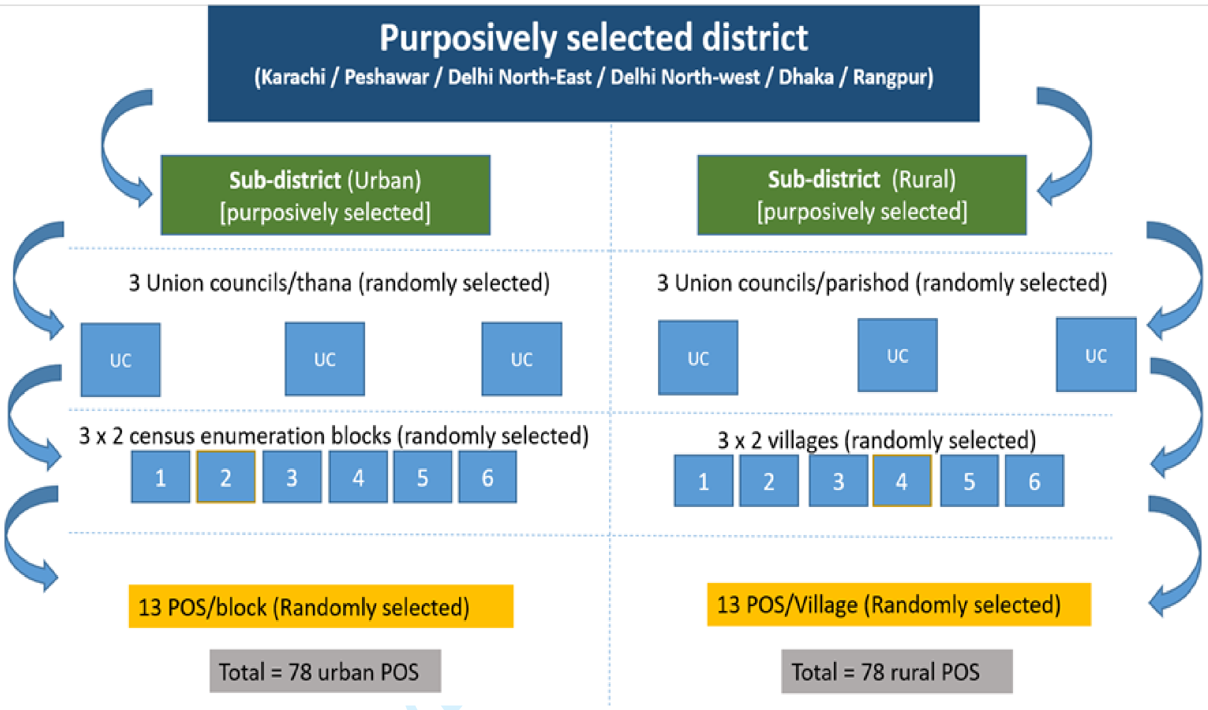

Fig 1. General sampling strategy to be used at all sites.

a. Within each of the selected administrative areas (District/Division), we will purposively select one predominantly urban and one predominantly rural sub-district. We will use the available local government documents, latest census and expert opinion of local researchers to identify two sub-districts/area at each site, one predominantly urban and the other predominantly rural/peri-urban.
b. We will randomly select three smaller administrative units per sub-district. These will be our Primary Sampling Units (PSU).
c. From each PSU, we will randomly select two enumeration blocks/neighbourhood areas/villages; these will be our Secondary Sampling Units (SSU).
d. From each SSU we will recruit up to 13 POS.
e. Required sample size/country is 290, through this strategy we will be able to recruit up to 312 POS.

For peer review only - http://bmjopen.bmj.com/site/about/guidelines.xhtml

Appendices

1. Field Protocol for Data Collection
2. Product Inventory Form
3. Product In-house assessment tool
4. Point of sale observation tool
5. Point of sale Interview tool
6. Details of the in-country ethical approvals

For peer review only

A.

For peer review only

Appendix 1

ASTRA Sellers Study Field Protocol

After selection of villages and enumeration blocks in rural and urban areas respectively through ASTRA sellers study sampling strategy, data collectors will be sent to these villages and enumeration blocks for mapping of the POS and collection of samples for In-House assessment. Following the is the field protocol for Mapping and Sample collection.

Inclusion criteria for POS

A POS will be eligible for inclusion in the study if it sells any form of Smokeless Tobacco. The common types of tobacco POS that are eligible for our study include:

a. General/departmental stores
b. Petrol pump/gas station stores
c. Beer or liquor stores
d. *Paan* (betel-quid) shops
e. Grocery stores
f. Exclusive tobacco shops
g. Discount shops
h. Mobile vendors/carts
i. Stationary carts
j. Any Other ________________. (Country teams can add specific POS not in the list)

The data collectors will ascertain the eligibility of an establishment by first inquiring about the availability of ST at that establishment and then proceeding for procurement of ST samples for the "In-house analysis" component of the study. Once they have established the POS as eligible, they will drop a pin on the Google Map as per the method used for "Mapping of POS".

Walking Protocol

The data collector will observe the following guidelines to map the POS in villages and enumeration blocks.

A. *When Map of the Roads is available*

1. Obtain the map of the specific area (Village/enumeration block) with all the primary roads marked on it.
2. Mark the geographic boundaries of that village/enumeration block on the map.
3. Number all the roads for identification and ease e.g. 1,2,….n
4. Identify the start point of each road as **"A"** and end point as **"B"**.
5. Start from point **A** of the road No.1, and map all the POS available on both sides of the road until you reach point **B** of the road.
6. Repeat the process until all the roads are covered.

For peer review only - http://bmjopen.bmj.com/site/about/guidelines.xhtml

7. While mapping the POS at each road ask the shopkeepers for POS away from the roads. Visit those POS and map them.

B. *When Map of the Roads is not available*

1. Identify the geographic boundaries of the village or enumeration block on the map.
2. Identify and mark all the primary roads on the map with the help of key informants.
3. Number all the roads for identification and ease e.g. 1,2,….n
4. Identify the start point of each road as **"A"** and end point as **"B"**.
5. Start from point **A** of the road No.1, and map all the POS available on both sides of the road until you reach point **B** of the road.
6. Repeat the process until all the roads are covered.
7. While mapping the POS at each road ask the shopkeepers for POS away from the roads. Visit those POS and map them.

Notes:

- In case if the shop is closed, and you suspect that it as a ST POS, map it by standing at the door of the shop. Mention in the comments as "closed".
- In case of mobile vendor/carts, map at the location where you find them.

For peer review only

Mapping of POS

Since this component uses Images from **Google Maps**, it cannot be published. The authors are willing to share the detailed method upon request.

Purchase of Products

Data collectors will be sent in pairs to map the POS. Arriving at the first shop one data collector will purchase the products and another one will map the shop;

1. The purchaser will visit the counter and ask for all the available brands of following smokeless tobacco (ST) products.
   a) Naswar
   b) Paan/ Betel Quid
   c) Gutka
   d) Tobacco Gum
   e) Gul
   f) Snus
   g) Zarda
   h) Nasal Snuff
   i) Khaini
   j) Others__________(Country Specific)

Complete **unique packs sample** by purchasing all unique smokeless tobacco packs available.
Unique Pack Definition: any pack with at least one difference in an exterior feature of the pack including: size, brand name, presentation, colors, packaging material (i.e. hard, soft, tin), and inclusion of a promotional item.

a. Face the counter and request to purchase one of each unique ST pack.
b. Capture the price of each pack. Place a sticker on the back of the pack and record the price on the sticker.
   i. For retailers where the price is not given, do not request nor accept a reduced price if the vendor offers a discount due to the large quantity being purchased.
c. Ask the shopkeeper to confirm that one of each ST pack they sell has been acquired and purchase any additional packs identified.
d. Also acquire one of the most commonly sold cigarette pack. Put a price tag on the pack. If cigarette packs is not available with the shop, continue with the process.
e. Place the packs in a zip lock bag. Pay for the entire purchase and retain a receipt to submit for reimbursement.
   i. Record on the receipt the ID of the area and shop, number of packs purchased, total price of the purchase, and date.
   ii. If the retailer does not provide receipts use a page and record the information listed above. Put the page along with the packs in a zip lock bag and write the area and the shop ID on the bag with a permanent marker.
f. Move to the next shop and ask for ST products other than purchased at previous shop.
g. Put stickers, write price, put in zip lock bag and put the IDs.
   i. If unique packs, other than previously purchased, are not available then move to the next shop.

For peer review only

As **two or more teams** will be collecting data simultaneously at **different locations**. Each team will purchase the available ST products. During **In-house assessment** of the products **duplication** will be removed.

Coding of each unique product will be done during In-House assessment.

In case if the shop is closed, no consent is given, do not sell STor do not sell ST to strangers, move to the next shop. Ask each shop for new products. If you find a new product, purchase and label it. Otherwise, don't buy duplicate product brands.

Equipment needed for field collection

Shoulder Bags (For carrying products)	Rubber bands
Zip Lock Bags	Stickers
Pens	Cash
Writing Pads	Umbrella

Data entry

An excel sheet will be given to the Data collectors. This sheet will contain information regarding the mapping of POS. It will be filled by Data collectors accordingly and will be submitted to the field supervisors on daily basis.

For peer review only

Appendix 2

Data fields for the digital ASTRA SLT product inventory form*

a) Study site________ (P1- P2)(B1-B2) (I1-I2)

b) Product Type_______(01-18)

c) Product unique ID_________________ (Study site/Product Type/product number)

d) Name of brand___________________
E.g. Bacha Naswar, JM Gutka …………..

e) Packaged _____________________ (Yes/No)

f) Hard/soft pack/sachet __________________ (coding 1-4, 4 for other)

g) Country of make________________ (coding 0 -195) (0 = Unknown)

h) Price (in local currency)__________________ (number)

i) Type of product _______________(coding 1-18, 18 for other)

j) Analyses status _____________ (Yes/No)

k) Price mentioned on pack________________(Yes/No)

l) Tax detail mentioned on pack________________(Yes/No)

If yes

m) How much amount has been mentioned ____________

*Adapted from the TPackSS online product inventory/registration form, available at https://globaltobaccocontrol.org/tpackss/

For peer review only

Appendix 3

In-house assessment of SLT products tool* (Adapted from TAPS guidelines)

•

**1. PACKAGING & LABELLING** (Yes =1, No = 0)	Cigarettes	Cigar/ Cigarillo	Beedi	Hukaah/shees [illegible]	Naswar	Gutka	Paan/ Betel [illegible]	Tobacco Gum	Mawah	Zarda	Gul	Snus	Nasal Snuff	Khaini	Others
a) Brand Name															
b) Name of the manufacturer only															
c) Address of the manufacturer only															
d) Name & Address of the manufacturer															
e) Contents listed (Yes/No)															
f) Weight per content is given (Yes/No)															
g) Country of Origin															
h) Statements of health claims															
i) Statements of harm e.g. harm to babies in the womb.															
j) Written health warnings (Yes/No)															
k) The language of health claims, Harm, Warnings (if No skip to k)															
I. Regional language															
II. National language															
III. English															
IV. Another foreign language															
l) Pictorial Health warning (Yes/No)(if No then skip to "m")															
m) The proportion of package does it cover															
I. 25%															

For peer review only

II. 50%															
III. 75%															
IV. 100%															
n) Provision of quit information on packaging (Yes/No)															
o) Use of culturally specific references (such as special images, symbols, or colours, etc.) (see your law or speak to local advocates for details)															
p) Presence of double packs															
q) Use of words that suggest added flavour (Yes/no)															
r) Use of words that suggest reduced strength (Yes/no)															

**2. PRICE AND TAXATION** (Yes =1, No = 0)	Cigarettes	Cigar/ Cigarillo	Beedhi	Hukaah/sheesha	Naswar	Gutka	Paan/ Betel Quid [illegible]	Tobacco Gum	Mawah	Zarda	Gul	Snus	Nasal Snuff	Khaini	Others
In what form is the product sold?															
a) Loosely sold?															
b) Loose, Mixed with other products (custom made)															
c) Packaged or wrapped by the retailer															
d) Pre-packaged by manufacturer															
e) If pre-packed then in the form of															
I. Packet															
II. Tin															
III. Bottle															
IV. Can															
V. Sachet															
VI. Box															

For peer review only

VII. If other please specify															
VIII. With branding															
f) The minimum unit of sale															
I. By quantity															
II. By weight															
g) Price of Unit customer pays															
h) Price of Unit retailer pays															

*this is the general tool. Countries are allowed to add any country specific provisions into the items.

For peer review only

Appendix 4

POS observation tool

ASTRA - POINT OF SALE OBSERVATION TOOL (Adapted from STARS)

1. Date of visit:__/__/____ Start Time: __:__ End Time: __:__

2. Coder Name/ID:_______________________

3. Point of Sale ID:_____/ ________ (Study site code/serial number)

4. Store Type: (Choose one)

a) General/Departmental Store ☐
b) Petrol pump/Gas station store ☐
c) Beer or Liquor store ☐
d) Grocery store ☐
e) Exclusive Tobacco shop ☐
f) Tea Stall ☐
g) Mobile cart ☐
h) Stationary cart ☐
i) Other (specify e.g. Discount shop):____

5. Can you survey this store? [If not, then select an option below and STOP.]

a) Yes, I can ☐
b) No, store does not exist ☐
c) No, store is closed ☐
d) No, environment unsafe for me ☐
e) No, asked to leave before completing the survey ☐
f) Other (specify):____

SECTION 1: OBSERVATION (POS display for mobile or stationary cart should be considered exterior)

6. EXTERIOR

Are there any point-of-sale displays of the tobacco products **OUTSIDE** the shop? (Yes = 1, No =0)	Cigarettes	Cigar/ Cigarillo	Beedi	Hukaah/sheesha	Naswar	Gutka	Paan/ Betel Quid [illegible]	Tobacco Gum	Mawah	Zarda	Gul	Snus	Nasal Snuff	Khaini	Others
a) Shelf edging															
b) Dummy packs															
c) Display packs															
d) Display stands															
e) Any other types of advertising?															

7. Is the point of sale vendor within _________meter (average distance) vicinity of any educational institution?

8. Does store display a health warning sign (OUTSIDE) against tobacco use? (If No skip to 9) ☐

Products	Written	Graphical

For peer review only

a) Smoking tobacco		
b) Smokeless Tobacco		

INTERIOR

9. Does the store have a pharmacy counter? (Yes = 1, No =0) ☐

10. Does store display a health warning sign (INSIDE) against tobacco use? (If No skip to 11) ☐

Products	Written	Graphical
a) Smoking tobacco		
b) Smokeless Tobacco		

11.

Are there any point-of-sale displays of the tobacco products **INSIDE** the shop? (Yes = 1, No =0)	Cigarettes	Cigar/ Cigarillo	Beedi	Hukaah/sheesha	Naswar	Gutka	Paan/ Betel Quid	Tobacco Gum	Mawah	Zarda	Gul	Snus	Nasal Snuff	Khaini	Others
a) Shelf edging															
b) Dummy packs															
c) Display packs															
d) Display stands															
e) Any other types of advertising?															

12.

**PRODUCTS OBSERVATION** (Yes = 1, No = 0)	Cigarettes	Cigar/ Cigarillo	Beedi	Hukaah/sheesha	Naswar	Gutka	Paan/ Betel Quid	Tobacco Gum	Mawah	Zarda	Gul	Snus	Nasal Snuff	Khaini	Others
a) Sold here?															
b) Visible															
c) Loosely sold here?															
d) Loose, mixed with other products (custom made)															
e) Product within 1 feet range of confectionaries, toys, candy, gum, slushy/soda machines, or ice cream?															
f) Products within 3 feet range of floor?															
g) Any price promotions/Credit?															

For peer review only

h) Cross-product promotion with tobacco? (Give any other related product free on purchase of the mentioned product)															

13. SALES TO/BY MINORS*

(Yes =1, No = 0)	**Smoking tobacco**	**Smokeless tobacco**
a) Did you observe any sales assistants under the age of 18 years in the POS?		
b) If yes, what are their expected ages? Please list.		
c) Did you observe the vendor was selling any tobacco product to young people under the age of 18?		

If you observe sale of ST to/or by a potential minor please make an effort to ask their age for confirmation.

For peer review only

Appendix 5

POS vendor interview tool

(The products in the columns and their unit of sales will be revised after the inventory study)

**14. PRICES & Sale**		Cigarettes	Cigar/ Cigarillo	Beedhi	Hukaah/sheesha	Naswar	Gutka	Paan/ Betel Quid with tobacco	Tobacco Gum	Mawah	Zarda	Gul	Snus	Nasal Snuff	Khaini	Others
a) Unit price that customer pay (in local currency)																
b) Unit price that retailer pay																
c) Sales Tax (in local currency)																
d) Total number of units sold per day	By Quantity															
	By Weight															
e) f) How was the price obtained																
I. Cashier/ owner/ shop keeper																
II. Advertised price																

15. SUPPORT FOR VIABLE ALTERNATIVES

Why do you sell tobacco products? Tick all that apply. (Options should not be disclosed until the shopkeeper answer. Tick all that apply and then probe with the options remaining)	**Smoking tobacco products**	**Smokeless tobacco products**
a) Good profit margin		
b) Products are in demand		
c) Family business		
d) No other viable alternative		
e) Brings people into my shop to buy other things		
f) Other reasons (please state)		
Would you ever consider stop selling SLT? (Yes = 1, N0 = 2)		
If yes, for which of the following reasons? Tick all that apply (if No skip to 16) (Options should not be disclosed until the shopkeeper answer. Tick all that apply and then probe with the options remaining)		
I. A reduction in profit margin		
II. A reduction in demand		
III. Stricter regulations and inspections		
IV. Limited supply		
V. A viable alternative		
VI. Awareness about its harms		
VII. Other reasons (please state)		

For peer review only

16. SUPPLIES

Where do you get your supplies? (Tick all that apply)	Cigarettes	Cigar/ Cigarillo	Beedhi	Hukaah/sheesh	Naswar	Gutka	Paan/ Betel	Tobacco Gum	Zarda	Mawah	Gul	Snus	Nasal Snuff	Khaini	Others
a) Wholesalers (Cash and carry)															
b) Manufacturers															
c) Importers															
d) Unknown source															
e) Another shop/ store															
f) Others (Please state)															

Why do you buy this product from a particular supplier? (Tick all that apply)	Cigarettes	Cigar/ Cigarillo	Beedhi	Hukaah/sheesha	Naswar	Gutka	Paan/ Betel Quid	Tobacco Gum	Mawah	Zarda	Gul	Snus	Nasal Snuff	Khaini	Others
a) They are the only supplier															
b) They can supply the required amount															
c) They are cheap															
d) They are nearby															
e) They are a friend or family member															
f) They sell products or brands my customers want															
g) They give me incentives															
h) Another reason (please state)															

What is the nature of the incentive by a particular supplier? (Tick all that apply)	Cigarettes	Cigar/ Cigarillo	Beedhi	Hukaah/sheesha	Naswar	Gutka	Paan/ Betel Quid	Tobacco Gum	Mawah	Zarda	Gul	Snus	Nasal Snuff	Khaini	Others
I. Discount															

For peer review only

II. Promotional Goods															
III. Point-of-sale displays															
IV. Free sample															
V. Others (please state)															

Where are your suppliers based (Tick all that apply)	Cigarettes	Cigar/ Cigarillo	Beedhi	Hukaah/sheesha	Naswar	Gutka	Paan/ Betel Quid	Tobacco Gum	Mawah	Zarda	Gul	Snus	Nasal Snuff	Khaini	Others
a) Local															
b) Abroad															
c) If Abroad, Where?															
d) Do you have contact details of the supplier?															
e) If "d" Yes, Can you Provide the contact details															
f) If "e" Yes, Contact Details of suppliers	**(Type of Product)**														
	**(Type of Product)**														
	**(Type of Product)**														
	**(Type of Product)**														

For peer review only

17. TOBACCO ADVERTISING, PROMOTION, AND SPONSORSHIP

(Yes =1, No = 0)

How do you (retailer) try to retain your tobacco customers? Tick all that apply.	**Smoking tobacco**	**Smokeless tobacco**
a) Offer discounts		
b) Offer promotional goods		
c) Offer credit lines		
d) Good customer relationships		
e) Other ways (please state)		

18. SALES TO/BY MINORS

(Yes =1, No = 0)	**Smoking tobacco**	**Smokeless tobacco**
d) Do you have any sales assistants under the age of 18 years?		
e) If yes, what are their ages? Please list.		
f) Are they allowed to sell tobacco products?		
g) Do you ever sell any tobacco products to young people under the age of 18?		
h) If no, why not?		

**PROFIT**	Amount in local currency
a) Roughly what amount of profit do you make from daily sales (Overall)	
b) What amount of profit comes from daily cigarettes sale	
c) What amount of profit comes from daily smokeless tobacco sale	

19. EDUCATION AND PUBLIC AWARENESS

(Yes =1, No = 0)

Are you aware of any laws that impact your business of selling tobacco products?	**Smoking tobacco**	**Smokeless tobacco**
a) If yes, please state which law(s) and how it affects your business		
Are you visited by any inspector or regulator to inspect and/or test your tobacco products?	**Smoking tobacco**	**Smokeless tobacco**
a) Inspect products		
b) Test products		
c) Check your license or registration		
d) How many visits did you have in the last 3 years?		
e) What happens after the inspection?		
I. If found not in compliance, nothing happens		
II. If found not in compliance, there is a penalty		
III. If found in compliance, nothing happens		
IV. If found in compliance, you are given a certificate		
V. Any other consequences?		
Do you have to disclose the contents of smokeless tobacco products to any statutory body?		
Do you have to send the products to a laboratory, for testing after Inspection?		

Signature of the interviewer___________________________

For peer review only

Appendix 6

Details of in-country ethical approvals

S.No	Country	Name of the Ethics Committee	Date of approval	Reference of approval
1	Bangladesh	The Bangladesh Medical Research Council's National Research Ethics Committee	03/01/2019	BMCRC/NREC/2016-2019/961
2	India	Health Ministry's Screening Committee (HMSC)	13/03/2019	HMSC/2018-2675
3	Pakistan	National Bioethics Committee of Pakistan	28/10/2018	4-87/NBC-355/19/1695

For peer review only
